# The Proteome of the Isolated *Chlamydia trachomatis* Containing Vacuole Reveals a Complex Trafficking Platform Enriched for Retromer Components

**DOI:** 10.1371/journal.ppat.1004883

**Published:** 2015-06-04

**Authors:** Lukas Aeberhard, Sebastian Banhart, Martina Fischer, Nico Jehmlich, Laura Rose, Sophia Koch, Michael Laue, Bernhard Y. Renard, Frank Schmidt, Dagmar Heuer

**Affiliations:** 1 Junior Research Group `Sexually Transmitted Bacterial Pathogens´, Robert Koch Institute, Berlin, Germany; 2 Junior Research Group `Bioinformatics´, Robert Koch Institute, Berlin, Germany; 3 Interfaculty Institute for Genetics and Functional Genomics, Ernst-Moritz-Arndt-University, Greifswald, Germany; 4 Advanced Light and Electron Microscopy (ZBS 4), Robert Koch Institute, Berlin, Germany; 5 ZIK-FunGene Junior Research Group `Applied Proteomics´, Interfaculty Institute for Genetics and Functional Genomics, Ernst-Moritz-Arndt-University, Greifswald, Germany; Harvard Medical School, UNITED STATES

## Abstract

*Chlamydia trachomatis* is an important human pathogen that replicates inside the infected host cell in a unique vacuole, the inclusion. The formation of this intracellular bacterial niche is essential for productive *Chlamydia* infections. Despite its importance for *Chlamydia* biology, a holistic view on the protein composition of the inclusion, including its membrane, is currently missing. Here we describe the host cell-derived proteome of isolated *C*. *trachomatis* inclusions by quantitative proteomics. Computational analysis indicated that the inclusion is a complex intracellular trafficking platform that interacts with host cells’ antero- and retrograde trafficking pathways. Furthermore, the inclusion is highly enriched for sorting nexins of the SNX-BAR retromer, a complex essential for retrograde trafficking. Functional studies showed that in particular, SNX5 controls the *C*. *trachomatis* infection and that retrograde trafficking is essential for infectious progeny formation. In summary, these findings suggest that *C*. *trachomatis* hijacks retrograde pathways for effective infection.

## Author Summary

The important human pathogen *Chlamydia trachomatis* causes 100 million new infections each year world-wide. It replicates inside the infected host cell in a unique vacuole, the inclusion. Currently, the nature, and specifically the protein composition of the inclusion, is poorly defined. Here, we described the host cell-derived inclusion proteome by quantitative proteomics using a newly established method to purify inclusions from infected epithelial cells. We showed that the inclusion is a complex intracellular trafficking platform that is well embedded into the organellar network and interacts with host cells’ antero- and retrograde trafficking pathways. Particularly, SNX1, 2, 5 and 6, components of the retromer, are recruited to the inclusion and seem to control the infection. We found also that retrograde trafficking is essential for *Chlamydia* progeny formation. Our study provides new insights into how the obligate intracellular bacterium *C*. *trachomatis* interacts with the eukaryotic host cell and subverts host cell functions for productive infection.

## Introduction

With 100 million new infections per year, *Chlamydia trachomatis* is the most frequently sexually transmitted bacterial pathogen world-wide [1]. *C*. *trachomatis* replicates inside a membrane-bound vacuole, the inclusion, and has a unique cycle of development, alternating between two distinct bacterial forms. The elementary body (EB) is spore-like, infectious but non-dividing. In contrast, the reticulate body (RB) is non-infectious but replicative. After internalization of the EB, the bacteria are found inside the inclusion, which is segregated from the lysosomal degradation pathway. EBs then differentiate into RBs, which replicate inside the growing inclusion. At mid-infection time points the inclusion is packed with replicating RBs that start to re-differentiate into EBs [2]. The surrounding inclusion membrane is the interface between the bacteria and the host cell. This membrane is actively modified by insertion of bacterial proteins and is not permissive for diffusion of molecules of 520 Da and larger [3]. It contains classical bacterial inclusion proteins of the Inc-protein family as well as non-classical Inc proteins [4]. Furthermore, a growing number of cellular proteins have been described to associate with the *Chlamydia* inclusion, but a global picture of proteins contributing to the inclusion is currently missing.

Membranes compartmentalize the eukaryotic cell into different organelles, including those of the secretory pathway and the endo-lysosomal system. In the secretory pathway, cargo is modified to address it to and then to transport it to its designated destination. The endo-lysosomal system functions in internalization of molecules from the plasma membrane (PM) or the extracellular space, followed by sorting of these molecules either for degradation in the lysosomes or for retrograde transport to different organelles, including the Golgi apparatus (GA). The human retromer is a multi-protein complex essential for recycling of cargo receptors into the tubular endosomal network and transports them to the trans-Golgi network (TGN) [5]. In human cells, the retromer consists of a membrane-deforming and a cargo recognition subcomplex, which are composed of the sorting nexins (SNX) 1, 2, 5, 6 and the vacuolar protein sorting-associated proteins (VPS) 26, 29, 35, respectively [6]. On endosomes, SNX dimers bind to phosphatidylinositol phosphates (PIPs) via their phox homology (PX)-domains. Additionally, these SNXs contain a Bin-Amphiphysin-Rvs (BAR) domain that recognizes membranes with high curvature and induces membrane tubulation, which is thought to support sorting of retrograde receptors out of the endo-lysosomal pathway [7]. Interaction with the cargo recognition subcomplex eventually leads to vesicle formation and the enclosed cargo is transported along microtubules to the TGN [8,9].

Proteomic studies of phagosomes isolated using latex-beads have greatly increased our knowledge about the biogenesis and function of these organelles [10–12]. Furthermore, the biochemical purification of vacuoles containing *Salmonella enterica*, *Mycobacterium avium*, *Rhodococcus equi* and *Legionella pneumophila* also fostered our understanding of the host cell protein composition of these unique intracellular compartments [13–17].

Here, we describe a two-step protocol for the isolation of high purity *C*. *trachomatis* serovar L2 inclusions at mid-cycle. Using LC-MS/MS based proteomics combined with ss isotope labeling by amino acids in cell culture (SILAC), we identified 351 host cell proteins that are significantly enriched in the proteome of isolated inclusions, representing the host cell-derived *Chlamydia* inclusion proteome. Enrichment analysis of this data showed that the *C*. *trachomatis* inclusion is a complex intracellular compartment that interacts with components of the retromer. Confocal studies confirmed the recruitment of SNX1, 2, 5 and 6 to the inclusion and further suggested that the retromer subcomplexes are at least partially separated at the inclusion membrane. Functional analyses of the retromer by RNA interference and by treatment with Retro-2, an inhibitor of retrograde transport of toxins and viruses, revealed that knockdown of SNX5 resulted in an increase in infectious progeny whereas Retro-2 treatment inhibited the formation of infectious bacteria. Taken together, these results show a previously unknown association of SNXs with *C*. *trachomatis* inclusions and provide evidence for a new role of SNXs during bacterial infections that appears to be independent of the classical SNX-BAR retromer complex.

## Results

### Isolation of *C*. *trachomatis* inclusions from HeLa cells 24 h p.i.

**Fig 1:**
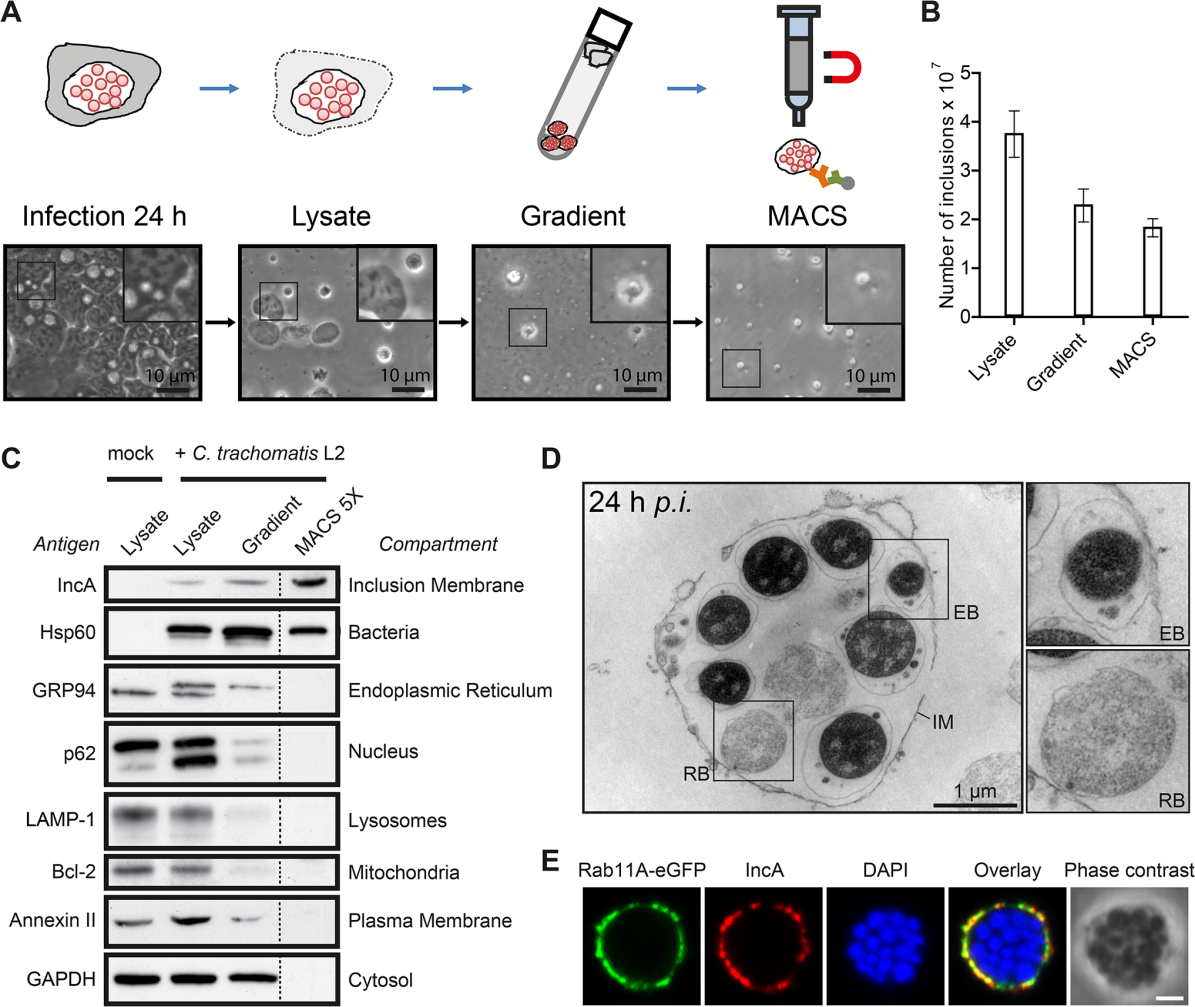
Isolation of *C*. *trachomatis* inclusions from HeLa cells at 24 h p.i. A) Workflow of the MACS purification procedure. Inclusions were isolated from *C*. *trachomatis* L2 infected cells. Different fractions of the purification procedure were analyzed by phase contrast microscopy. B) Visually intact inclusions were counted in different fractions (n = 3; error bars, SD). C) Immunoblot analysis of different steps of the inclusion purification procedure. In the first three fractions, equal volumes of approximately equal protein amounts were loaded, the MACS 5X lane contains 5X the number of inclusions contained in the infected cell lysate lane. The indicated proteins were detected with specific antibodies. The subcellular compartments indicate the primary location of the probed proteins. D) TEM of gradient purified and washed inclusions. IM, inclusion membrane; EB, elementary body; RB, reticulate body. E) Inclusions were gradient purified from *C*. *trachomatis* L2 infected HeLa cells transiently expressing Rab11A-eGFP, fixed, immunostained with IncA antibodies (red) and analyzed using LSCM. The DNA was stained with DAPI (blue). Scale bar, 2 μm; n = 2. See also S1 Fig.

We established an isolation method for *C*. *trachomatis* inclusions at mid-infection time points, based on a two-step protocol originally described for the isolation of *Legionella*-containing vacuoles from amoebae (Fig 1A) [16]. Infected HeLa cells were lysed and the obtained cell lysate containing inclusions was separated on a self-forming Percoll gradient. Gradient fractions were taken and analyzed for presence of bacterial and cellular proteins by immunoblotting and for presence of intact inclusions by phase contrast microscopy (S1A and S1B Fig). The high density fractions harboring intact inclusions (S1A and S1B Fig) were collected, pooled and further purified by magnet assisted cell sorting (MACS) using an antibody specific for IncA, a bacterial transmembrane protein located in the inclusion membrane [18]. Presence and numbers of inclusions were monitored by phase contrast microscopy (Fig 1A and 1B). Counting of visually intact inclusions at each purification step showed that ~50% of *C*. *trachomatis* inclusions present in the cell lysate could be isolated (Fig 1B). The purity of the different fractions was assessed by immunoblotting, using antibodies specific for marker proteins of different cellular compartments and for chlamydial proteins (Fig 1C). Lysate of infected and uninfected HeLa cells showed presence of organelles such as the nucleus, endoplasmic reticulum (ER), lysosomes, mitochondria, cytosol and the PM (Fig 1C). After separation by Percoll gradient, inclusions were enriched as indicated by an increase in IncA and Hsp60 signals, accompanied by a decrease in signals for cellular compartments. MACS purification resulted in a fraction that contained chlamydial inclusions that were nearly completely devoid of cellular contaminants as monitored by immunoblotting (Fig 1C). Obtained inclusion fractions were then analyzed by electron and fluorescence microcopy (Fig 1D and 1E). Transmission electron microscopy (TEM) demonstrated the presence of inclusions that contained both bacterial forms surrounded by the inclusion membrane (Fig 1D). To validate the presence of cellular proteins in the isolated inclusion fraction, inclusions were purified from cells expressing a Rab11A-eGFP fusion protein that is known to be associated with *C*. *trachomatis* inclusions [19]. Immunofluorescence (IF) staining and confocal microscopy of isolated inclusions revealed that Rab11A-eGFP signal co-localized with IncA in a rim-like pattern (Fig 1E). In summary, these data show that we are able to isolate *C*. *trachomatis* inclusions at mid-infection time points.

### Identification of inclusion-associated, cellular proteins by quantitative proteomics

**Fig 2:**
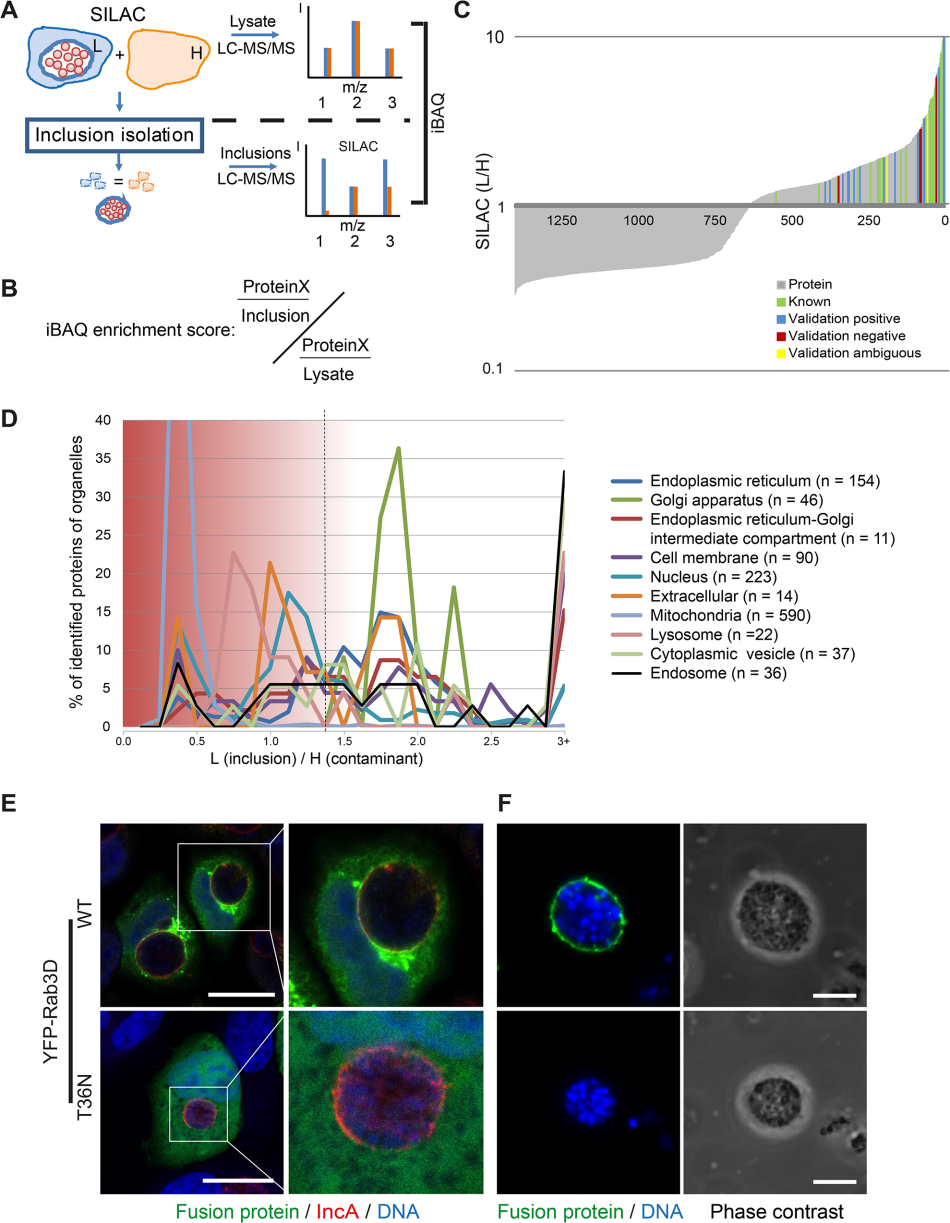
Quantitative proteomics of isolated *C*. *trachomatis* inclusions. A) Workflow of inclusion host proteome analysis. The scheme of three different peptides (1–3) measured by LC-MS/MS shows idealized SILAC ratios for peptides derived from different classes of proteins. (1) Ideal inclusion associated protein. Contaminations are expected to be identical for H and L labeling, corresponding to peptide (2). Proteins that are in part contaminations as well as inclusion associated have a lower L/H ratio (3). The enrichment of proteins in the inclusion fraction compared to the total lysate is calculated based on intensity based absolute quantification (iBAQ). B) The iBAQ enrichment score was calculated by dividing the proportional amount of protein specifically associated with the inclusion by the amount of this protein in the total lysate fraction. For more details see main text and S1 Text. C) Each protein identified and quantified in triplicate was plotted with its SILAC ratio (grey lines, lin/log plot). Known (green lines) and newly validated proteins (blue, yellow and red lines) are indicated. D) Distribution of annotated organellar proteins along the SILAC ratios. Mean SILAC ratios of proteins were pooled into bins of 0.125 (n = 1400). The total number of proteins mapping to the subcellular localization term was determined and the percentage in each bin plotted along against the SILAC ratios. Data points were connected for better visibility. SILAC ratios of 3 and above were pooled in the 3+ bin. Dashed line, approximate cutoff for enrichment. E) Validation of inclusion associated proteins using fluorescent fusion proteins. Confocal IF images showing HeLa cells expressing the indicated fluorescent fusion proteins (green), infected with *C*. *trachomatis* L2 (MOI 2). Cells were fixed 24 h p.i. and stained for inclusion membrane (IncA red) and DNA (DAPI, blue). Scale bar, 20 μm. F) Validation by purified inclusions. Inclusions were gradient purified from cells expressing the indicated fusion protein using a small scale protocol and analyzed by LSCM, DNA was stained with DAPI. Scale bar, 5 μm. For supplemental data see S2–S4 Figs.

To identify host cell proteins specifically associated with isolated *C*. *trachomatis* inclusions, SILAC was applied [20]. Using this method, we were able to control for non-specific, co-purifying proteins during the isolation procedure (Fig 2A). The proteins that are *bona fide* constituents of the inclusion were expected to have a high ratio of L label vs. H label (SILAC ratio) of one peptide species, whereas contaminants were expected to have SILAC ratios close to 1 in the inclusion fraction (Fig 2A). The abundance of inclusion-associated proteins in enriched fractions and proteins in total cell lysates was calculated using iBAQ (intensity based absolute quantification) which estimates the abundance of proteins based on the sum of peak intensities of all peptides matching to a specific protein, divided by the number of theoretically observable peptides [21]. Despite limited accuracy, this method provides additional information especially for highly abundant proteins in addition to the SILAC based exclusion approach. Based on this method, we quantified the relative contribution of each protein to the total proteome of the lysate and the inclusion using sum total normalization for the proteins in each fraction. Only proteins that passed the SILAC exclusion approach were considered for the inclusion proteome. The quotient of the values for the inclusion and the lysate resulted in the enrichment score for proteins which were overlapping in the two datasets (iBAQ enrichment score) (Fig 2B and S1 Text). For proteins that were not found in our lysate proteome, we used a recently published very high coverage dataset of the HeLa proteome [22] for approximation of the protein abundance in the cell lysate. We performed experiments in three biological replicates. Analysis of the raw data by MaxQuant resulted in the identification of 1400 host cell proteins in the inclusion fraction (Fig 2C) and 2002 host cell proteins in the cell lysate. To characterize potential organellar contaminants, subcellular localization data of all proteins in the inclusion fraction was retrieved from UniprotKB [23] and annotations were plotted according to their SILAC ratios (Fig 2D). This data clearly showed that proteins from mitochondria, the nucleus and the PM appeared at SILAC ratios of 1 and lower, and therefore are most likely contaminants of the inclusion fraction. The majority of proteins annotated with the terms cytoplasmic vesicle, ER, ER-Golgi intermediate compartment (ERGIC), GA and lysosome were separated from the contaminants with a SILAC ratio above 1.5, demonstrating an enrichment of these proteins in the inclusion isolation procedure of infected cells vs. uninfected cells (Fig 2D). Statistical testing based on the SILAC ratio distribution in the lysate and in the inclusion fractions revealed 351 host proteins that were significantly enriched in the inclusion fraction, of which 253 were highly reliable due to the presence of high ratios in all three replicates, resulting in small multiplicity adjusted p values of below 0.01 (S2A Fig). An additional 98 proteins were qualified as enriched with reduced statistical confidence (multiplicity adjusted p value < 0.05, S2B Fig). These 351 host proteins are thus considered to be inclusion associated (S1 Table). Of the approximately 50 host proteins known to be recruited to *Chlamydia* inclusions, 23 were identified in our analysis (S2 Table). These proteins included 14-3-3 ß, CERT, VAP-A, VAP-B, Rab1, Rab6A, Rab11A and Rab14 [19,24–27]. These known inclusion-associated proteins were distributed across the SILAC ratios, further increasing our confidence in the generated inclusion proteome data set (Fig 2C).

We next validated the obtained data by confocal microscopy. To this end, 26 newly found inclusion-associated proteins with different SILAC ratios were chosen. Proteins of interest were either detected after ectopic expression of tagged fusion proteins or by visualizing endogenous proteins using specific antibodies (S3 and S4 Figs). Non-fused eGFP was used as control. Localization of these proteins in infected cells was assessed after IF staining counterstained with an IncA-specific antibody to visualize the inclusion membrane and were then analyzed by laser scanning confocal microscopy (LSCM) (Figs 2E and S3 and S4). To confirm the presence of the fluorescently tagged proteins in the inclusion fraction, inclusions were also isolated from cells transiently expressing the respective fusion proteins (Figs 2E and S3). In total, 26 proteins were included in the validation process. From these 26 proteins, 19 proteins were validated positively, either by inclusion isolation or by immunofluorescence microscopy. Among these positive hits were YFP-RAB3D wild-type, VCP-eGFP, eGFP-SYNGR2, eGFP-Rab8A, GFP-Syntaxin 7, STIM1 and Sec22b. As expected, no co-localization of eGFP was observed (S3 Fig). Five proteins were evaluated as false-positive including eGFP-Cofilin-1, Sequestosome-1 and Arginase-1 (S3 and S4 Figs). For two proteins the localization to the inclusion as monitored by fluorescence microscopy was ambiguous (S3 and S4 Figs). Furthermore, recruitment of Rab3D appears to be an active process, as the dominant negative form of Rab3D (YFP-RAB3D T36N) was not found at the inclusion (Fig 2E). Taken together, we have identified 351 host cell proteins that are significantly enriched in the isolated inclusion fraction and thus contribute to the host cell-derived inclusion proteome.

### Global analysis of the host cell-derived inclusion proteome reveals a complex intracellular compartment enriched for retromer components

**Fig 3:**
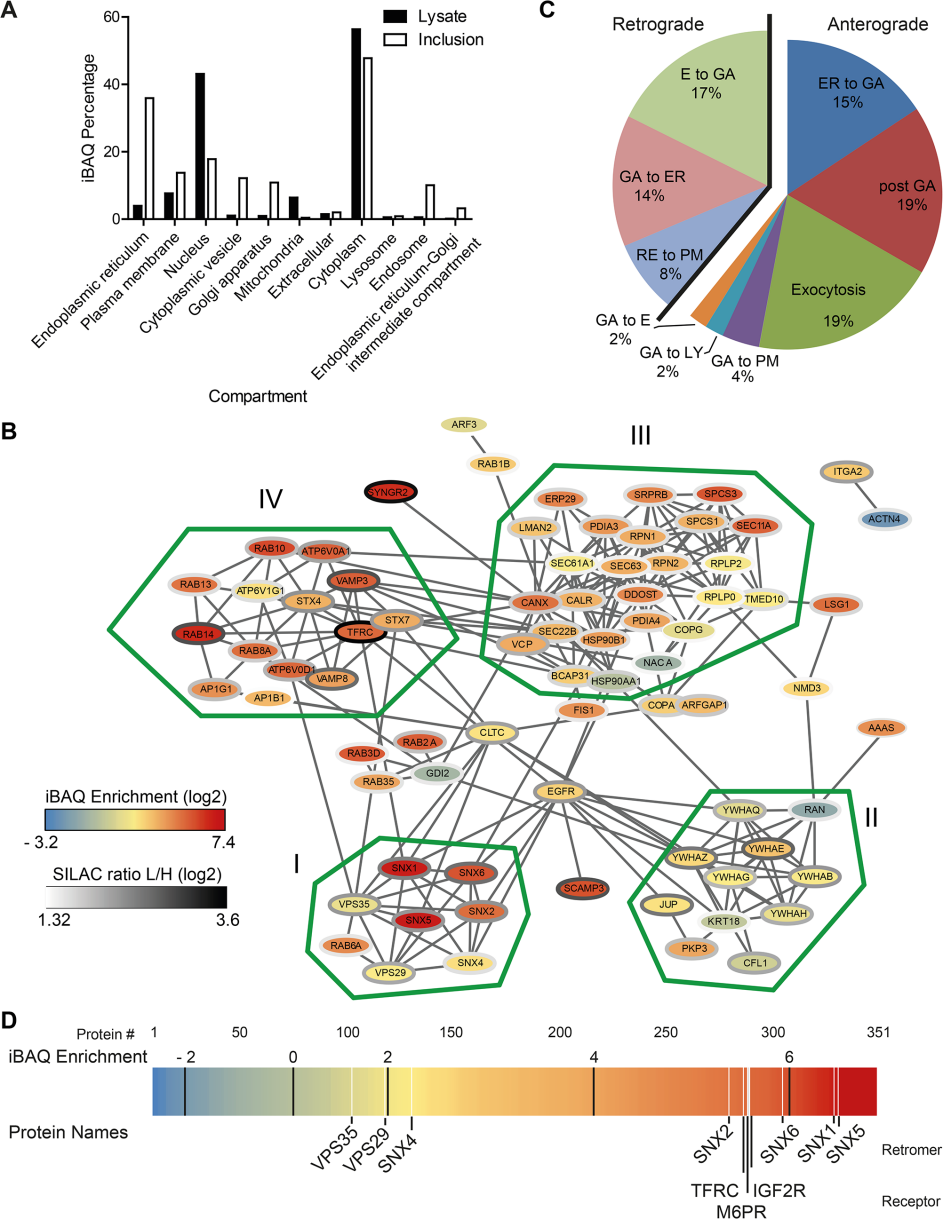
Global analysis of the host cell derived inclusion proteome. A) Proteins that were reliably found and quantified in the inclusion proteome or the total cell lysate were annotated with subcellular localization data from UniprotKB. Proteins were quantified according to their iBAQ intensity and the abundance of proteins annotated with the indicated term was summed. One protein can have annotations for several categories and organelles. B) Protein-protein interactions of inclusion associated proteins annotated with the highly enriched GO term `establishment of protein localization´. Connecting lines indicate interactions as reported by STRING database in standard settings. Color of the node represents the enrichment score, the color of border of the nodes are colored according to the SILAC ratio. Main clusters of interacting proteins are encircled with a green line and labeled I-IV. C) Proteins annotated with the GO term `vesicle mediated transport´ were further classified as involved in retrograde or anterograde transport (n = 35) and respective subcategories. Three proteins with incomplete GO annotation were added manually (Rab1B, Rab12, VPS29). Five proteins were annotated with both retrograde and anterograde transport pathways (LMAN1, Rab11B, Rab14, TMED10 and VAMP3). Two additional proteins were annotated with two subcategories (Stx7, VAMP8, both anterograde). E = endosome, ER = endoplasmic reticulum, GA = Golgi apparatus, EX = exocytosis, PM = plasma membrane, LY = lysosome, RE = recycling endosome. D) Enrichment of proteins at the inclusion calculated using iBAQ. Each protein is represented by a line colored according to its enrichment score. Black lines indicate the ranges of log2 transformed fold enrichment. Proteins of interest, white lines. For supplemental data see S15 Fig.

Based on this core host cell-derived inclusion proteome, we analyzed the contribution of cellular organelles to the proteome of isolated inclusions. Subcellular localization data of the identified proteins was retrieved from UniprotKB to calculate the relative contribution of different organelle types to the obtained proteomes. We observed a clear enrichment of proteins annotated as components of the ER, the PM, the ERGIC, the GA, endosomes and cytoplasmic vesicles (Fig 3A). As expected, relative depletion was seen for proteins annotated as nuclear and mitochondrial (Fig 3A).

Next, we performed a gene ontology (GO) enrichment analysis based on GO of biological processes (GOBP) (S3 Table). The most highly enriched single term apart from ER-specific processes was `establishment of protein localization´ (GO:0045184) with a p value of 3.94 x 10^–13^ and a total of 86 proteins contributing to this category. Proteins from this term were analyzed for specific complexes of interacting proteins using STRING 9.1 [28]. This interaction map revealed four clusters of highly interacting proteins including a cluster composed of the SNX-BAR retromer, a complex involved in retrograde trafficking from endosomes to the TGN (Fig 3B). The most granular (i.e. highly resolved) GO term apart from ER-related processes was `vesicle-mediated transport´ (p = 1.66 x 10^–10^, GO:0016192, n = 58; n = 72 including child terms). To further characterize these trafficking pathways that are putatively involved in the function of the inclusion, we analyzed the contribution of proteins involved in anterograde and retrograde transport to the proteome (Fig 3C). Proteins involved in retrograde trafficking constitute 39% of these proteins, with retrograde transport from endosomes to the GA being the largest group within the retrograde trafficking group (17% of total). Strikingly, components of the human retromer were highly enriched in the host cell-derived inclusion proteome compared to total cell lysates, including proteins of the SNX family and the retrograde-transport cargo protein Ci-M6PR, which are among the 25% most highly enriched proteins (Fig 3D). In summary, the host cell-derived proteome of *C*. *trachomatis* inclusions reveals a complex intracellular compartment enriched for SNX-BAR retromer and suggests that the inclusion interacts with multiple cellular trafficking pathways, including this retrograde transport pathway.

### SNXs are recruited to the *C*. *trachomatis* inclusion

**Fig 4:**
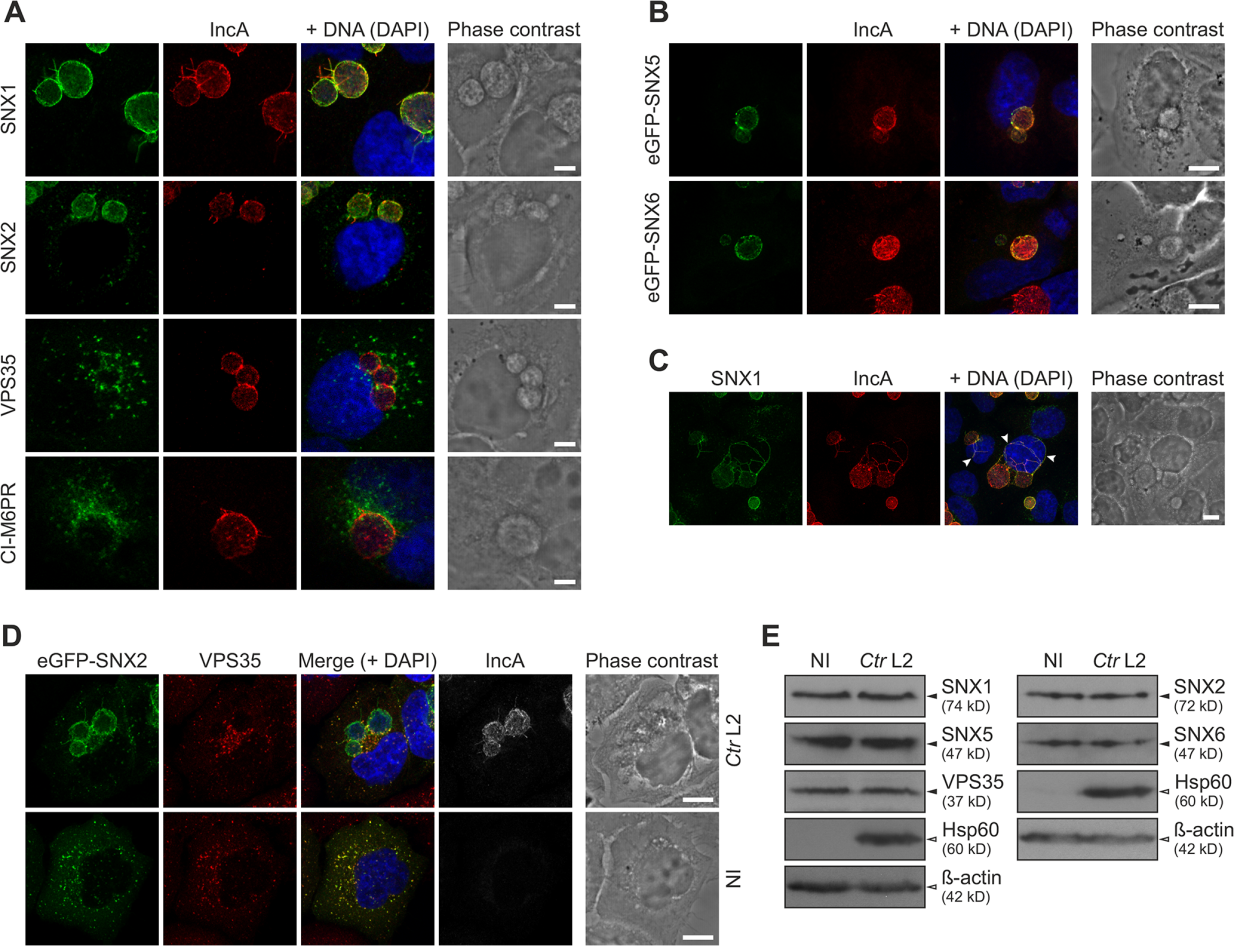
*C*. *trachomatis* infection shows SNX recruitment to the inclusion and leads to partial separation of the retromer subcomplexes. A) Confocal IF images showing localization of retromer components during *C*. *trachomatis* L2 infection (MOI 1). HeLa cells were fixed 24 h p.i. and stained with indicated antibodies; DNA was stained with DAPI (blue). Scale bar, 5 μm; n = 3. B) Confocal IF images showing localization of eGFP fusion proteins of SNX5 and SNX6 recruited to the inclusion. HeLa cells were infected with *C*. *trachomatis* L2 (MOI 2) 4 h prior to transfection, fixed at 24 h p.i. and stained with indicated antibodies; DNA was stained with DAPI (blue). Scale bar, 10 μm; n = 2. C) Confocal IF images showing fibers positive for SNX1 and IncA in *C*. *trachomatis* L2 infected HeLa cells (MOI 2). Cells were fixed at 24 h p.i. and stained with indicated antibodies; DNA was stained with DAPI (blue). Images show a maximum intensity projection of a z-stack, arrows indicate SNX1/IncA fibers. Scale bar, 10 μm; n = 3. D) Confocal IF images showing co-localization of eGFP-SNX2 fusion protein with endogenous VPS35 in *C*. *trachomatis* L2 infected (*Ctr* L2, MOI 2) and uninfected (NI) HeLa cells. HeLa cells were infected 4 h prior to transfection, fixed at 24 h p.i. and stained with indicated antibodies; DNA was stained with DAPI (blue). Scale bar, 10 μm; n = 2. E) Immunoblots of retromer components during *C*. *trachomatis* L2 infection (MOI 2) at 24 h p.i. n = 2. For supplemental data see S5–S7 Figs.

Based on the high enrichment of retromer components on *C*. *trachomatis* inclusions, we performed IF studies using antibodies specific for SNX1, SNX2, VPS35 and Ci-M6PR to confirm localization of these proteins to the inclusion using LSCM (Figs 4A and S5A). SNX5 and SNX6 localizations were analyzed after ectopic expression of eGFP-SNX fusion proteins (Figs 4B and S5B). In uninfected HeLa cells, signals for SNX1 and SNX2, were found in punctuated structures in the cytosol consistent with the reported endosomal localization of these SNXs (S5A Fig). In contrast, in *C*. *trachomatis-*infected HeLa cells, SNX1, SNX2, eGFP-SNX5 and eGFP-SNX6 were detected as a rim-like staining pattern that partially co-localized with the bacterial inclusion marker, IncA (Fig 4A and 4B). Recruitment of these SNXs was specific, as other members of the SNX family (SNX3 and SNX12) did not co-localize with the inclusion membrane (S6 Fig). Furthermore, these SNXs were also found in IncA-positive fibers emanating from the inclusion body (Fig 4C). Interestingly, VPS35 and Ci-M6PR did not show a rim-like inclusion-staining pattern, but rather were depicted as small punctuated structures adjacent to the inclusion, suggesting that the membrane-deforming and receptor-recognition subcomplex of the human retromer are at least partially disconnected at the inclusion (Fig 4A). To confirm the separation of these two subcomplexes, SNX2 and VPS35 were simultaneously localized in infected and uninfected cells (Figs 4D and S7). Interestingly, at the inclusion, a separation of the two signals was observed. Co-localization of the two signals in defined punctuated structures at the inclusion was rarely seen (Fig 4D). In contrast, in uninfected cells, signals for both subcomplexes were clearly co-localized (S7 Fig). Pearson's correlation coefficient also suggested only a moderate co-localization of the two signals at the inclusion, whereas a strong correlation was detected in punctuate-structures in the cytoplasm of either infected or uninfected cells (S7 Fig). To avoid artifacts due to overexpression of eGFP-SNX2, we also performed experiments in cells expressing eGFP-VPS35 and stained for endogenous SNX2 (S7B Fig), confirming that the retromer subcomplexes do not co-localize at the inclusion, indicating separation or dissociation of the retromer complex. No difference in protein abundance for all tested retromer components was detected in *C*. *trachomatis*-infected cells compared to control cells (Fig 4E). These observations demonstrate that during *C*. *trachomatis* infection SNX-BAR proteins become recruited to the inclusion and the localization of the two retromer subcomplexes is dramatically changed.

### Silencing of SNX5 promotes *C*. *trachomatis* infectious progeny formation

**Fig 5:**
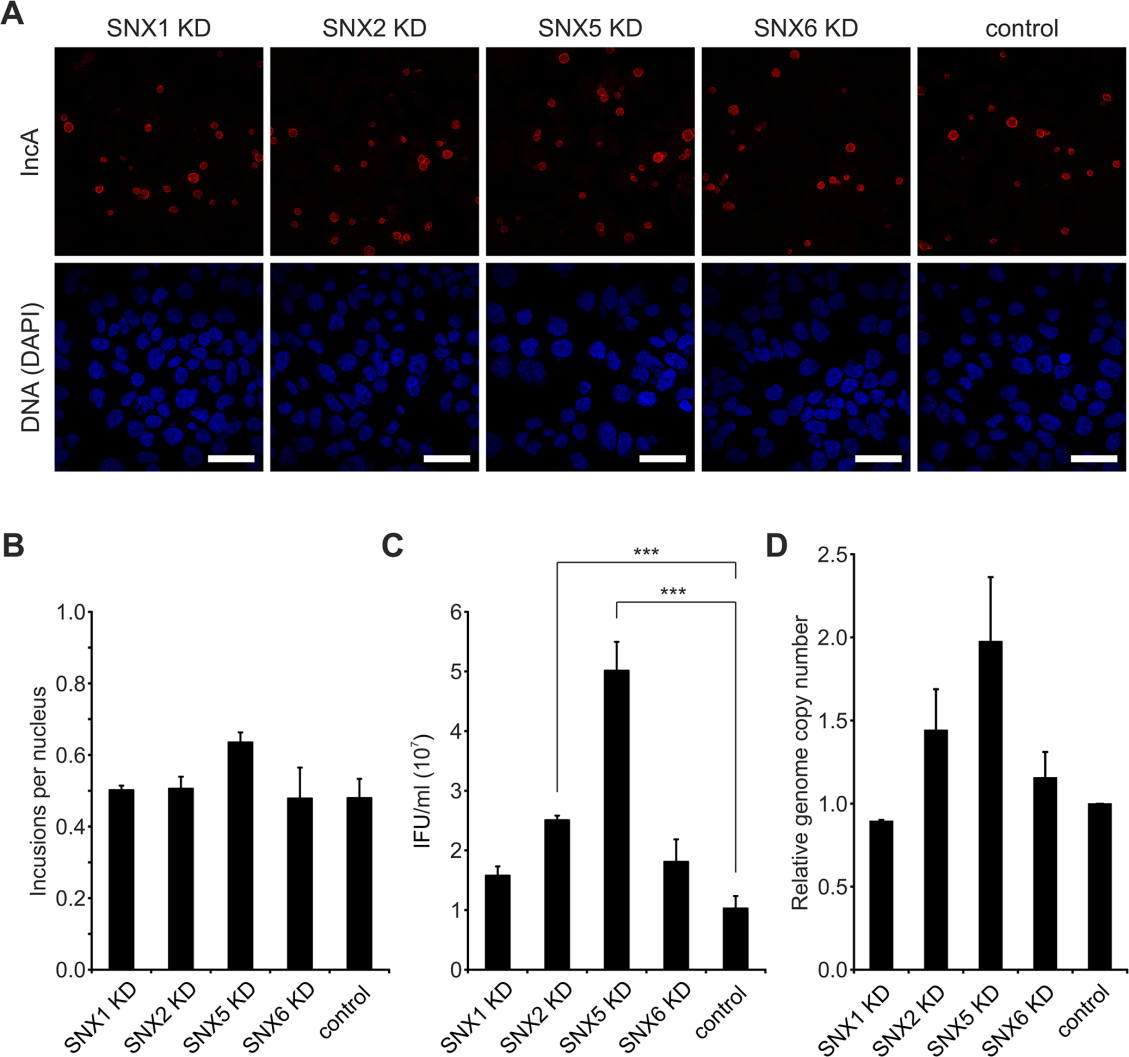
Silencing of SNX5 promotes *C*. *trachomatis* infectious progeny formation. A) Confocal IF images showing *C*. *trachomatis* L2 infected (MOI 0.5), siRNA-treated HeLa cells. Cells were transfected with siRNA pools against indicated genes or control siRNA (AllStars), fixed at 24 h p.i. and stained with indicated antibodies; DNA was stained with DAPI (blue). Scale bar, 50 μm; n = 2. B) Analysis of the primary infection in siRNA-treated HeLa cells from panel A. Inclusions and nuclei were microscopically counted in 5 fields of view and ratio was calculated (n = 2; error bars, SE). C) Reinfection assay assessing the effect of SNXs knockdown on infectious progeny formation 48 h p.i. n = 3; error bars, SE; *** indicates p value < 0.005. D) Graph showing the effect of SNXs knockdown on relative bacterial genome copy number in *C*. *trachomatis* L2 infected (*Ctr* L2, MOI 0.5) HeLa cells at 48 h p.i. Genome copy number was determined by qRT-PCR (n = 2; error bars, SE). For supplementary data see S8 Fig.

Given that SNX-BAR proteins of the retromer are recruited to the *C*. *trachomatis* inclusion at 24 h p.i., we tested whether knockdown of retromer components by RNA interference (RNAi) affects *C*. *trachomatis* infection including inclusion formation and development of infectious EBs. We used pools of small-interfering RNAs (siRNAs) to target SNX1, 2, 5 and 6. Silencing of these proteins did not affect the formation of inclusions as analyzed by inclusion size and numbers (Fig 5A and 5B). Interestingly, silencing of SNX5 resulted in a clear increase in infectious EBs compared to control transfections (Fig 5C). SNX1, 2 and 6 knockdown also increased infectious progeny, albeit only marginally (Fig 5C). Genome copy numbers upon silencing of the different SNX proteins were slightly affected, showing the strongest increase in genome copy numbers in SNX5 knockdown cells (Fig 5D). Immunoblotting confirmed that upon knockdown, the targeted SNX-BAR proteins were drastically reduced compared to control treated cells (S8A Fig). We confirmed published data that silencing of SNX5 also resulted in a decrease in protein level of SNX1 (S8A Fig). To elucidate if the observed increase in infectious progeny in SNX5 knockdown cells is dependent on co-regulating the abundance of the other SNX proteins, we silenced SNX5 in combination with SNX1, 2 or 6 and measured infectious progeny formation (S8B Fig). Number of infectious bacteria was increased under all combinational knockdown conditions compared to control, suggesting that other SNX proteins do not contribute to the observed increase in infectious progeny formation in SNX5 knockdown cells. Knockdown efficiencies in these double knockdown cells were confirmed by immunoblotting (S8A and S8C Fig). Taken together, these results suggest that individual SNX-BAR proteins might have distinct functions in addition to controlling the retrograde transport of specific receptors. SNX5 in particular might be a rate-limiting factor and involved in intracellular replication of *C*. *trachomatis*, most likely independently of the other SNX-BAR retromer components.

### Retro-2 treatment reduces infectious progeny formation of *C*. *trachomatis*

Retro-2 was identified in a high-throughput screen for small molecules that inhibit the toxicity of the plant toxin ricin in cell culture and was additionally found to efficiently protect cells from secreted bacterial toxins, including Shiga-like toxin and cholera toxin by inhibiting retrograde trafficking of these toxic agents from the endosomes to the GA or the ER without affecting trafficking of endogenous cellular retrograde-transport cargo proteins including Ci-M6PR [29].

**Fig 6:**
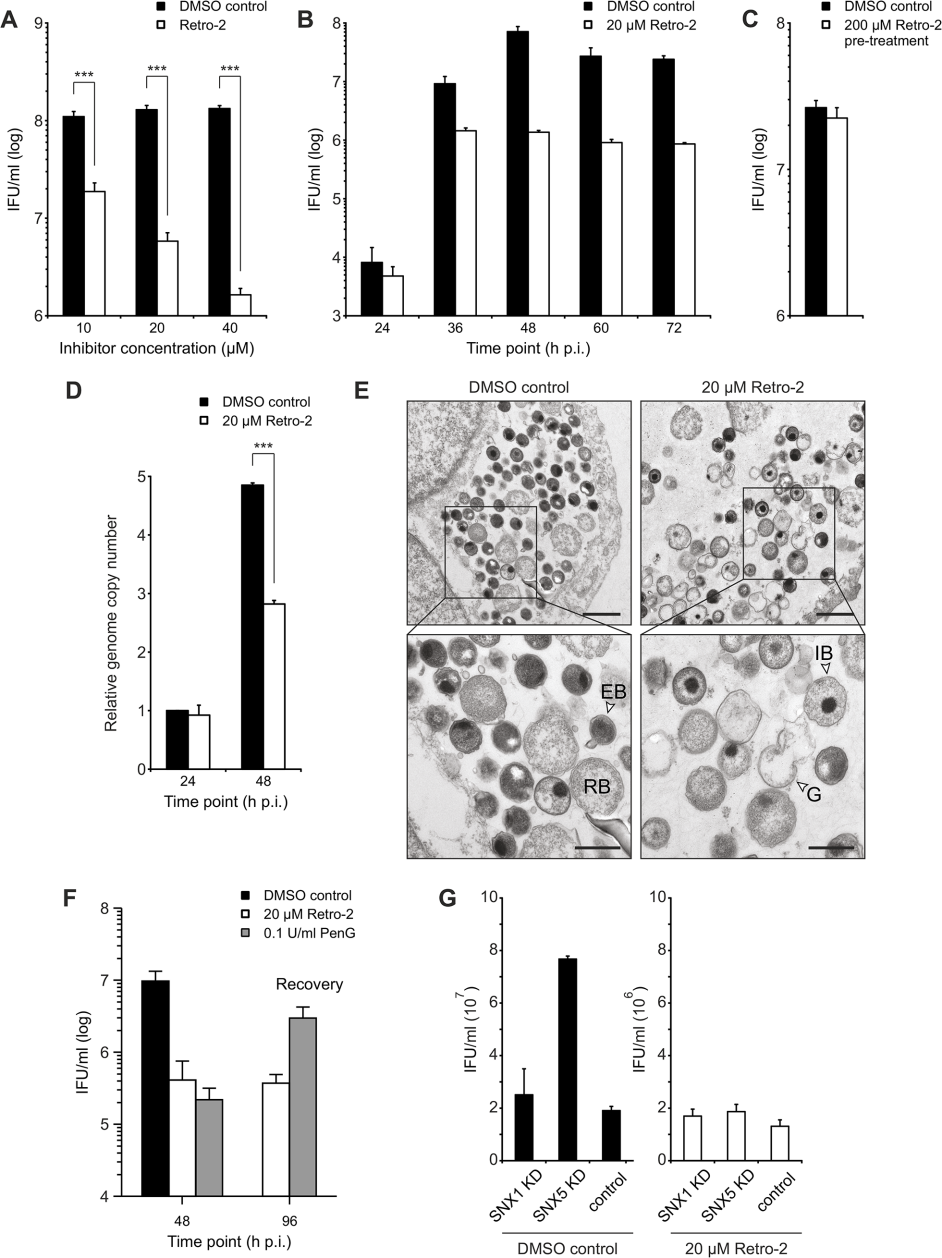
Retro-2 reduces infectious progeny formation of *C*. *trachomatis*. A) to C) Reinfection assays assessing the effect of Retro-2 on infectious progeny formation. HeLa cells were infected with *C*. *trachomatis* L2 (MOI 2) and, at 8 h p.i., cells were either treated with indicated concentrations of Retro-2 or DMSO. Cells were harvested and inclusion forming units (IFU) per ml were determined A) at 48 h p.i. (n = 3; error bars, SE; *** indicates p value < 0.005) or B) at different time points after infection (n = 3; error bars, SE). C) *C*. *trachomatis* EBs were treated with 200 μM Retro-2 or DMSO as solvent control for 30 minutes at room temperature before pelleting, followed by washing and infection of HeLa cells at MOI 2 for 48 h (n = 4; error bars, SE). D) Graph showing the effect of Retro-2 on relative bacterial genome copy number in *C*. *trachomatis* L2 infected (MOI 2) HeLa cells at 24 h and 48 h p.i. Cells were infected and, at 8 h p.i., either treated with indicated concentrations of Retro-2 or DMSO as solvent control. Genome copy number was determined by qRT-PCR (n = 4; error bars, SE; *** indicates p value < 0.005). E) TEM of *C*. *trachomatis* L2 infected (*Ctr* L2, MOI 2) HeLa cells at 48 h p.i. treated with indicated concentrations of Retro-2 or DMSO. G = ghost, IB = intermediate body, RB = reticulate body, EB = elementary body. Scale bar, 1 μm (upper micrographs) and 500 nm (lower micrographs). F) Reinfection assay demonstrating the recovery of infectious progeny after treatment with Retro-2 and PenG. HeLa cells were infected and treated with the indicated compound from 8 h p.i (Retro-2) or 24 h (PenG) until 48 h p.i. Cells were washed with fresh infection medium and incubated until 96 h p.i. to allow for recovery of the bacteria (n = 3, error bars = SE). G) Reinfection assay assessing the effect of Retro-2 and SNX knockdown on infectious progeny formation. HeLa cells were transfected with siRNA pools against indicated genes or control siRNA (against Luciferase), infected with *C*. *trachomatis* L2 (MOI 2), treated with Retro-2 at 8 h p.i., harvested at 48 h p.i. for reinfection assay (n = 2; error bars, SE).

SNX1, SNX2 and eGFP-SNX5 recruitment to the inclusion was detected starting from 12 h p.i. Interestingly, association of eGFP-SNX6 with the inclusion was detected slightly later (S9 Fig). At 16 h p.i. all inclusions were positive for the four different SNX proteins, coinciding with the expansion of the inclusion (S9 Fig). Taking this into account, we treated cells prior to SNX recruitment (8 h p.i.) with different concentrations of Retro-2 and assessed the formation of infectious EBs by re-titration at 48 h p.i. Treatment of *C*. *trachomatis*-infected cells with Retro-2 resulted in a dose-dependent decrease by more than one order of magnitude in EB numbers compared to the vehicle control (Fig 6A). Reducing the treatment duration from 40 h to 28 h by shifting the time point of Retro-2 addition to 20 h p.i. still showed a decrease in infectious progeny formation albeit to a much lesser extent (S10 Fig). The progression of the chlamydial developmental cycle was not affected as EB formation peaked at 48 h p.i. under both conditions, even though fewer EBs were recovered from the Retro-2 treated sample (Fig 6B). Retro-2 treatment reduced the size of *C*. *trachomatis* inclusions at 24 h and 48 h p.i. by about 40% without changing the shape of the inclusions (S11 Fig). Pretreatment of EBs with high Retro-2 concentrations (200 μM) before infection did not reduce infectious progeny compared to vehicle control (Fig 6C) and numbers of bacterial genomes were only slightly affected by the inhibitor (Fig 6D). To elucidate the effect of Retro-2 treatment on induction of chlamydial persistence, the ultrastructure of Retro-2 treated and control infected cells were determined by electron microscopy (Fig 6E). No signs of persistence in Retro-2-treated infections, as characterized by the appearance of larger aberrant *Chlamydia* forms were observed. Quantification of bacterial numbers confirmed that Retro-2 treatment affects replication of the bacteria which is in agreement with Retro-2 effects on genome copy numbers (S12 Fig and Fig 6D). Interestingly, we also detected a slight increase in numbers of intermediate bodies and ghosts in *C*. *trachomatis* inclusion grown in Retro-2 treated cell cultures compared to solvent control (S12 Fig).

A recovery assay in which infected cells were treated with Retro-2 from 8–48 h p.i., followed by removal of the inhibitor and additional incubation for 48 h in the absence of the inhibitor, confirmed that Retro-2 does not induce chlamydial persistence (Fig 6F). These experiments demonstrated that treatment of *C*. *trachomatis* infected cultures with Retro-2 strongly reduced the number of infectious bacteria at 48 h p.i. and upon removal the number of infectious bacteria remained on a low level. In contrast, the bacteria nearly completely recovered after removal of the well-known persistence inducer, penicillin G (Fig 6F).

In summary, our data show that *C*. *trachomatis* infections are Retro-2 sensitive resulting in smaller inclusions with slightly less bacteria inside, but with a strong defect in the generation of infectious EBs without induction of persistence.

We have shown that SNX5 and Retro-2 act on *C*. *trachomatis* infections, albeit with opposite effects on the bacteria. To further determine which effect is dominant, cells were treated with siRNA pools specific for SNX5, SNX1 and luciferase. Luciferase was used as non-targeting control while SNX1 knockdown served as additional control, as it did not significantly increase the EB numbers (Fig 5C). Infected knockdown cells were either treated with a single dose of Retro-2 at 8 h p.i. or mock-treated. Infectious progeny number was determined 48 h p.i. (Fig 6G). As expected, in vehicle-treated SNX5 knockdown cells, the characteristic increase in EB numbers upon knockdown of SNX5 was observed (Fig 6G). Interestingly, this increase in EB numbers in comparison to SNX1 knockdown and non-targeting control was lost upon Retro-2 treatment (Fig 6G). To assess whether Retro-2-sensitive retrograde transport is involved in recruiting SNX proteins to the inclusions, the localization of SNX proteins after Retro-2 treatment was analyzed at 12 h, 16 h and 24 h p.i. by confocal microscopy. In these imaging studies, no change in SNX localization was observed (S13 Fig). These data show that the increase in numbers of infectious EB after the silencing of SNX5 is Retro-2 sensitive whereas recruitment of SNX proteins to the inclusion appears to be Retro-2 insensitive.

## Discussion

The previous inability to isolate *Chlamydia* inclusions enforced severe experimental constraints and impeded progression in our comprehension of virulence mechanisms and the development of novel anti-chlamydial therapies. For example, recruitment of cellular proteins to the inclusion could only be addressed by microscopy. Direct biochemical evidence for the association of these factors with the inclusion membrane was therefore missing. To overcome this limitation, we established a method to isolate *C*. *trachomatis* inclusions at 24 h p.i. and analyzed isolated inclusions using a quantitative proteomics approach to decipher the host-derived *C*. *trachomatis* inclusion proteome.

We used the recently described protocol for the isolation of LCV from *D*. *discoideum* [16] as a starting point, but due to the fragile nature of the *C*. *trachomatis* inclusion, this protocol was heavily modified. As a result, we retained a two-step protocol but started with a Percoll-based gradient followed by immuno-magnetic separation using an IncA-specific antibody. One of the critical steps in the isolation protocol was the lysis of the infected host cells. We carefully tested different buffer and infection conditions, but the majority of inclusions were ruptured at this step resulting in a maximum recovery of 15% of the calculated initial numbers of inclusions. The yield in the following steps (gradient and MACS) was about ~50% amounting to a total recovery rate of about 8%. This recovery rate is in the range or even slightly higher than the yields obtained for *Legionella* containing vacuole isolations [16,30]. The second challenge was to find an optimal strategy for initial purification of the visually intact inclusions from cellular debris. We used isopycnic density gradient centrifugation to separate inclusions from host cell debris. We recovered the majority of inclusions in solution by fractionation of the gradient, but apparently the buoyant density of inclusions is very diverse, distributed across the range of densities of intracellular organelles, thus a subpopulation escaped our analysis which was distributed over the whole gradient without apparent peaks. It seems likely that these are inclusions that either contained large amounts of glycogen [31] or lipid droplets which are known to be translocated into the lumen of inclusions [32]. This translocation could have a considerable effect on their overall density. This speculation is supported by the absence of markers for lipid droplets in our proteome analysis. Moreover, we detected inclusions ranging in size from 3 μm up to 10 μm, representing the majority of expected inclusion sizes, possibly with a slight bias towards smaller inclusions, which could result from an increased fragility of larger inclusions.

The high sensitivity of modern LC-MS/MS-based proteomics demands an experimental design which includes a strategy to distinguish between *bona fide* components of the isolated compartment as well as co-purified contaminations. To this end, we used a SILAC-based exclusion approach in combination with label-free absolute quantification. A similar method was successfully used in a recent study to identify contaminants in purified latex bead-containing phagosome preparations [33].

Underlining the success of the purification and SILAC exclusion approach, we found a significant proportion of previously reported inclusion-associated proteins in our dataset. To further investigate the sensitivity of our assay, we ranked the proteins detected in a deep proteome of HeLa cells [22] by the iBAQ value of tryptic peptides, to see if highly abundant proteins are over-represented in the overlap with previously known inclusion-associated proteins (S14 Fig). Our limit for reliable detection of proteins with more than one peptide is slightly above the median iBAQ intensity in the HeLa cell lysate (S14 Fig). This is satisfying, considering the technical difficulties due to massive amounts of bacterial peptides present in our samples. However, based on these data, the true number of inclusion-associated proteins might be significantly higher than what we report here, probably around two times greater than the reported number based on known host proteins associated with inclusions. Furthermore, the SILAC exclusion approach has also some limitations, for example with proteins that have a high dissociation constant, which reduces the SILAC ratio due to exchange of L- for H-labeled proteins during the extended incubation time in cell lysate before MACS separation, thereby increasing the number of false negative classifications. These factors influence the number of reported proteins, but are all likely to reduce the reported number rather than to lead to false positives.

Whereas originally the inclusion was thought to be a separated compartment that acts as a niche devoid of host proteins [34], this picture has changed dramatically in recent years as indicated by the extensive interaction with cellular organelles and recruitment of specific proteins, often mediated by bacterial effectors, which was first described for 14-3-3 ß [27]. Interestingly, proteins annotated as nuclear, mitochondrial and lysosomal were significantly depleted in the *Chlamydia* inclusion proteome. Proteins assigned to other cellular organelles contributed significantly to the inclusion proteome, suggesting the inclusion is embedded in the intracellular trafficking network of the host cell. This conclusion supports the view that the *C*. *trachomatis* inclusion is a complex intracellular trafficking platform that exploits different pathways to foster optimal intracellular growth, rather than that of an isolated niche. For an obligate intracellular pathogen that lacks a number of genes for the biosynthesis of essential nutrients, this integration into the host cell organellar network seems reasonable to secure intracellular survival [35]. We noted redundancy in interactions which could reflect robustness of the intracellular lifestyle, which is further supported by the fact that *C*. *trachomatis* can infect and grow in an array of different cell types.

Detailed analysis of the host cell-derived inclusion proteome showed that *C*. *trachomatis* inclusions interact with the retromer, an important complex regulating retrograde transport of different cellular receptors and a pathway also hijacked by bacterial and plant toxins and distinct viruses to intoxicate and infect cells [6,36–38]. In *Chlamydia*-infected cells, the SNX-BAR proteins SNX1, 2, 5 and 6, are recruited to the inclusions decorating the inclusion in a rim-like staining pattern and are additionally found on IncA-laden fibers emanating from the inclusion body. In this context, it is interesting to note that *Salmonella enterica* serovar Typhimurium acquire SNX1 and SNX3, and SNX1 is found on spacious vacuole-associated tubules early in the infection process [39,40]. In uninfected cells, the PX and BAR domains of SNX-BAR proteins target these proteins to phosphoinositide-enriched, high-curvature membranes [41,42]. Phosphatidylinositol-4-phosphate (PI4P) has also been detected in the inclusion membrane by expression PIP-sensitive reporter proteins [43]. Whether the detected PI4P or additional bacterial proteins such as Inc proteins that are present in the inclusion membrane are involved in recruiting the SNX-BAR proteins to the inclusions is currently not known. Interestingly, the cargo recognition subcomplex of retromer showed only a punctual localization at the inclusion membrane. Consequently, there is partial separation of the two retromer subcomplexes at the inclusion membrane but not in other locations of infected cells. These observations support recent findings on the structure and function of the cellular retromer. Firstly, whereas the retromer complex is a stable hetero-pentamer in yeast cells, this association is much more transient in mammalian cells [44] and secondly, the two subcomplexes and the individual SNX-BAR proteins are involved independently of each other in trafficking of distinct cargo [45–47]. Functional analysis of SNX-BAR proteins using RNAi showed that in particular SNX5 knockdown resulted in an increase in infectious progeny. This may indicate that SNXs, and in particular SNX5, become segregated by recruitment to the *C*. *trachomatis* inclusion, thereby affecting the cellular retrograde trafficking pathways. The activity of the retromer complex has often been linked to processes controlling the sorting of cellular receptors including the epidermal growth factor receptor (EGFR) and M6PR [48,49]. SNX5 in particular has been implicated in EGFR trafficking and signaling in uninfected cells [48]. For *C*. *trachomatis* infections it has recently been demonstrated that EGFR activity is important for maturation of the inclusion by controlling calcium signaling and actin remodeling [50]. In light of these and our findings it is tempting to speculate that SNX5 recruitment to the inclusion alters e.g. EGFR transport and signaling inside the cells which in turn triggers calcium release and F-actin rearrangements. These changes then support the development of a proper *C*. *trachomatis* inclusion and are thus important for a successful infection. Alternatively, distinct SNX-BAR proteins control a currently not well-defined Retro-2-sensitive retrograde trafficking pathway that delivers distinct nutrients to the bacteria or alternatively could be related to factors controlling innate immunity. The idea of an innate immunity-related function of the retromer is further supported by the recently published observation in *Drosophila* that retromer can also control the Toll pathway [51].

The observed sensitivity towards the retrograde inhibitor Retro-2 also supports the view that retrograde transport is important for *C*. *trachomatis* progeny formation. The molecular target of Retro-2 is currently unknown but treatment results in displacement of the three t-SNAREs syntaxin (Stx) 5, 6 and 16 from membranes of the Golgi apparatus. These t-SNAREs are essential for retrograde transport of different cargo molecules to the TGN [52]. Interestingly, the localization of Stx6 to the inclusion has also been documented using microscopy and lack of Stx6 slightly but significantly reduced *C*. *trachomatis* infectious progeny [53,54]. Whether the strong inhibitory effect of Retro-2 treatment on *C*. *trachomatis* growth and infectious progeny formation is a result of mislocalization of different t-SNAREs from the inclusion or if additional proteins are also targeted by the treatment remains to be determined. Experiments are in progress to address Retro-2 dependent changes on a global level to determine these factors, which will potentially identify the molecular target of Retro-2 and might also uncover novel functions of the evolutionarily highly conserved retromer complex.

In summary, we have deciphered the core host cell-derived proteome of the *C*. *trachomatis* inclusion 24 h p.i. by quantitative proteomics of isolated inclusions. This data set describes the inclusion as a highly complex and interactive compartment that amongst others recruits proteins normally forming the membrane-binding subcomplex of the cellular SNX-BAR retromer. Of the subset of SNX-BAR proteins, SNX5 controlled the formation of infectious *Chlamydia* progeny in a Retro-2 sensitive pathway highlighting the importance of distinct SNX-BAR proteins and the retrograde transport for *C*. *trachomatis* infections. Thus, the development of a technique to isolate *Chlamydia* inclusions fosters our understanding of the inclusion composition, the contribution of cellular factors to inclusion formation and maintenance. This may pave the way for the development of axenic culture conditions and novel anti-chlamydial strategies.

## Materials and Methods

### Cell culture, infections and infectious progeny formation

HeLa cells were grown in Roswell Park Memorial Institute medium (RPMI, Gibco) 1640 supplemented with 10% fetal calf serum (FCS, Biochrom) at 37°C and 5% CO_2_ in a humidified incubator. The cells were routinely tested for *Mycoplasma* contamination via polymerase chain reaction (PCR) using the VenorGeM kit (Biochrom) according to manufacturer’s instructions. *C*. *trachomatis* L2 lymphatic isolate 434 Bu (ATCC: VR-902B) was propagated in HeLa cells. For more details on infections, determination of infectious progeny formation, the quantification of relative bacterial genome copy number, infection recovery assay, bacterial morphology assay and measurement of inclusion size, see S1 Text.

### Plasmid and siRNA transfections

For plasmid transfections, HeLa cells were grown to 80% confluency and transfected with Lipofectamine 2000 reagent (Invitrogen) according to manufacturer’s instructions. For knockdown of target host cell proteins, HeLa cells were transfected with pools of target specific siRNAs as described in S1 Text.

### Standard procedures and reagents

For the standard procedures TEM, IF, SDS-PAGE, immunoblotting, molecular cloning as well as used reagents, plasmids and oligonucleotides, see S1 Text.

### Gradient and MACS purification of inclusions

HeLa cells were infected with *C*. *trachomatis* (MOI 4) at 70–90% confluence. For standard isolations, 6 x 10^7^ cells were used. All steps were done on ice or in a cold room at 4°C. Cells were washed once with PBS and subsequently with ice cold HSMG buffer (20 mM HEPES, 250 mM sucrose, 1.5 mM MgCl_2_, 0.5 mM EGTA, pH 7.4). Cells were scraped into 6 ml lysis buffer (33% Percoll solution (Sigma), HSMG) supplemented with cOmplete EDTA free protease inhibitors (Roche). Lysis was performed by repeated passage through a ball homogenizer (Isobiotech) using 16 μm clearance and 11–13 passages.

The lysate was then separated on a self-forming Percoll gradient in a total volume of 16 ml by centrifugation at 35’000 x *g* for 30 minutes at 4°C (Beckmann RC-6 with Thermo Scientific F21-8x50y rotor). The lower 6 ml of the gradient were either used for MACS purification or crude inclusions were diluted six fold in HSMG and pelleted at 1500 x *g* for 10 minutes, followed by another wash and centrifugation at 1200 x *g* for 10 minutes.

For MACS separation, crude inclusions were incubated with rabbit αIncA (1:1000) antibody [55] for 1.5 h at 4°C, followed by incubation with MACS secondary goat anti-rabbit antibody (1:100, Miltenyi) for another 1.5 hours. Inclusions were mixed gently by inversion every 30 minutes. The crude inclusions were loaded on a MACS LS separation column (Miltenyi) column in steps of 2 ml and washed with three times the input volume of HSMG buffer. Inclusions were then eluted with 3 ml HSMG buffer after removal of the magnet, aided by gentle pushing using the supplied plunger. Counting of inclusions, the small scale isolation procedure for validation and processing of inclusions for IF and TEM are described in supporting information (S1 Text).

### SILAC experiments

For SILAC experiments, cells were grown in SILAC DMEM (PAA) containing dialyzed FCS (Biochrom), supplemented with H labeled L-arginine(^13^C_6_^15^N_4_) and L-lysine (^13^C_6_^15^N_2_) (Silantes) or non-labeled amino acids (L). Inclusions were isolated as described above but H labeled mock infected cells were mixed with equal amounts with L labeled infected cells prior to cell lysis. Inclusion samples were prepared for LC-MS/MS. 10% of the sample was used for direct injection after desalting. The remaining peptides were separated by strong anion exchange chromatography into 6 fractions before desalting, followed by LC-MS/MS. Lysate samples were prepared for LC-MS/MS without pre-fractionation. For more details, see S1 Text.

### Proteome data analysis

Tryptic peptides were analyzed using a data dependent method on a Q Exactive mass spectrometer (Thermo) coupled to a Ultimate 3000 nHPLC (Dionex) for separation by reverse phase chromatography. The resulting. raw files were analyzed in MaxQuant 1.3.0.5 [56]. Protein groups that had less than two unique + razor peptides in at least one experiment were filtered. See S1 Text for more details on SILAC enrichment analyses, abundance analyses using iBAQ and further bioinformatics analyses.

## Supporting Information

S1 Fig The inclusion membrane marker IncA sediments to high density fractions in a Percoll gradient. A) 6 x 10^7^ HeLa cells were infected with *C*. *trachomatis* L2 for 24 h (MOI 2). Cells were lysed in a ball homogenizer (16 μm clearance, 13 strokes) and subsequently fractionated on an in-situ formed 33% Percoll gradient in HSMG buffer. The gradient was fractionated into 16 fractions of equal volume (fraction 1: bottom, fraction 16: top). Fractions 1–4 were pooled, diluted in HSMG and washed twice (P). Equal volumes of each fraction (1–16) or concentrated washed inclusions (P) were prepared, separated on a 10% SDS-PAGE gel, western blotted and probed with specific antibodies against the indicated proteins. B) The majority of intact inclusions sediments to high density fractions in a Percoll gradient. Intact inclusions were counted for each fraction of a gradient prepared as described in A) but MOI 3, and the percentage was plotted. Error bars indicate standard deviation of three independent replicates.
(TIF)

S2 Fig Statistical test for enrichment in the inclusion fraction/ SILAC exclusion approach. Proteins were tested for enrichment in the inclusion fraction. The graph shows a bar diagram with the empirical distribution of the logarithm of the SILAC ratios of proteins that were found in both the inclusion and lysate fraction. The grey bars indicate the SILAC ratios of proteins found in the lysate which overlap with inclusion proteins, blue bars show proteins that are differentially enriched in the inclusion fraction. Red bars show proteins which were only found in inclusion dataset. Proteins enriched in the inclusion fraction are expected to have positive (L/H) SILAC ratios. A) Proteins of the inclusion fraction which show three SILAC ratios (blue and red) B) Proteins of the inclusion fraction which only show two SILAC ratios (blue and red). The highest bar was capped at 500. More proteins were used for the empirical lysate distribution compared to A, because the overlap for both proteins with two and three SILAC ratios was used.
(TIF)

S3 Fig Validation of inclusion associated proteins using fluorescent fusion proteins. A) IF images showing HeLa cells expressing the indicated fluorescent fusion proteins (green), infected with *C*. *trachomatis* L2 (MOI 2). Cells were fixed 24 h p.i. with 2% PFA and stained for IncA (inclusion membrane, red) and DNA (DAPI, blue). Scale bar, 20 μm. B) Validation by purified inclusions in live cell microscopy. Inclusions were gradient purified from cells expressing the indicated fusion protein using a small scale protocol and analyzed by LSCM, DNA was stained with DAPI. Scale bar, 5 μm. The “results” column indicates whether a protein was considered to be positively validated (+), not inclusion associated (-) or ambiguous (+-).
(TIF)

S4 Fig Validation of inclusion associated proteins using immunofluorescence. A) Confocal immunofluorescence images showing localization of ectopically expressed epitope tagged proteins in *C*. *trachomatis* L2 infected cells. HeLa cells were transfected, infected with MOI 2 and fixed at 24 h *p*.*i*. with 2% PFA in PBS, except for FLII-HA which was fixed with ice cold methanol. B) Confocal immunofluorescence images showing localization of endogenous proteins of interest. HeLa cells were infected with MOI 2 and fixed at 24 h *p*.*i*. with 2% PFA in PBS. A) and B) Cells were stained with indicated antibodies; DNA was stained with DAPI (blue). The results column indicates whether a protein was considered to be positively validated (+), not inclusion associated (-) or ambiguous (+-). Scale bar, 20 μm.
(TIF)

S5 Fig Localization of retromer components in uninfected cells. A) Confocal immunofluorescence images showing localization of retromer components in uninfected control cells. HeLa cells were fixed and stained with indicated antibodies; DNA was stained with DAPI (blue). Scale bar, 5 μm; n = 3. B) Confocal immunofluorescence images showing localization of eGFP fusion proteins of human SNX5 and SNX6 in uninfected control cells. HeLa cells were transfected, fixed and stained with indicated antibodies; DNA was stained with DAPI (blue). Scale bar, 10 μm; n = 2.
(TIF)

S6 Fig Co-localization of SNX3/VPS35 and SNX12/VPS25. Confocal immunofluorescence images showing co-localization of A) eGFP-SNX3 with endogenous VPS35 and B) eGFP-SNX12 with endogenous VPS35 in *C*. *trachomatis* L2 infected (*Ctr* L2, MOI 2) and uninfected (NI) HeLa cells. HeLa cells were infected 4 h prior to transfection, fixed at 24 h p.i. and stained with indicated antibodies; DNA was stained with DAPI (blue). Scale bar, 10 μm; n = 2.
(TIF)

S7 Fig Co-localization of SNX2/VPS35. A) Quantification of SNX2/VPS35 co-localization indicating Pearson’s correlation coefficient (*r*) in *C*. *trachomatis* L2 infected (*Ctr* L2, MOI 2) infected and uninfected cells either expressing eGFP-SNX2 or VPS35-eGFP. Correlation of the two signals was analyzed and quantified using ZEN 2010 software (Zeiss) in either the complete cell area (total), the cytoplasmic area of infected cells excluding the inclusion (cytoplasm) or directly at the inclusion (inclusion) (n = 2; error bars, SE). B) Confocal immunofluorescence images showing co-localization of eGFP-VPS35 fusion protein with endogenous SNX2 in *C*. *trachomatis* L2 infected (*Ctr* L2, MOI 2) and uninfected (NI) HeLa cells. HeLa cells were infected 4 h prior to transfection, fixed at 24 h p.i. and stained with indicated antibodies; DNA was stained with DAPI (blue). Scale bar, 10 μm; n = 2.
(TIF)

S8 Fig siRNA knockdown control. A) Immunoblot analysis of single siRNA knockdowns of retromer components in *C*. *trachomatis* L2 infected (*Ctr* L2, MOI 0.5) HeLa cells. n = 3. B) Reinfection assay assessing the effect of combinational SNXs knockdown on infectious progeny formation 48 h p.i. (n = 3; error bars, SE). C) Western blot analysis of combinational siRNA knockdowns of retromer components in *C*. *trachomatis* L2 infected (*Ctr* L2, MOI 0.5) HeLa cells. β-actin was used as loading control; n = 3.
(TIF)

S9 Fig Recruitment of SNX1, SNX2, eGFP-SNX5 and eGFP-SNX6 to the inclusion. Confocal IF images showing localization of SNX1, SNX2, eGFP-SNX5 and eGFP-SNX6 during *C*. *trachomatis* L2 infection (MOI 2) at 8 h, 12 h, 16 h, 20 h and 24 h p.i. and in uninfected HeLa cells. Cells were stained with indicated antibodies; DNA was stained with DAPI (blue). Images show maximum intensity projections of z-stacks. Scale bar, 10 μm; n = 2.
(TIF)

S10 Fig Retro-2 treatment at 20 h p.i. reduces infectious progeny formation. Reinfection assays assessing the effect of Retro-2 on infectious progeny formation. HeLa cells were infected with *C*. *trachomatis* L2 (MOI 2) and, at 8 h p.i., or 24 h p.i., cells were either treated with indicated concentrations of Retro-2 or DMSO. Cells were harvested and inclusion forming units (IFU) per ml were determined at 48 h p.i. (n = 3; error bars, SE; *** indicates p value < 0.005).
(TIF)

S11 Fig Retro-2 treatment results in smaller inclusions with regular shape. The size and number of inclusions was assessed by immunofluorescence microscopy. HeLa cells were infected with *C*. *trachomatis* (MOI 0.5). Retro-2 or DMSO was added at 8 h p.i. Cells were fixed with 2% PFA in PBS at the indicated time point. Immunostaining was performed against bacterial Hsp60 and epifluorescence microscopy pictures were randomly taken at an AxioVert40 inverted microscope. A) A script in ImageJ software was used to count the relative numbers and measure the area of inclusions. Asterisk indicates a p value below 0.05. B) Representative image of Retro-2 and DMSO treated cells at 24 h *p*.*i*. Scale bar = 100 μm.
(TIF)

S12 Fig Morphology analysis of Retro-2 treated HeLa cells. HeLa cells were infected for 48 h with *C*. *trachomatis* L2 (MOI 2). The cells were treated with 20 μM Retro-2 at 8 h p.i. or mock treated with DMSO. Cells were pelleted and fixed with glutaraldehyde before processing for TEM. Randomized images were taken from slices and the images were analyzed by eye for the distribution of different morphologies of *C*. *trachomatis*. G = ghost, IB = intermediate body, RB = reticulate body, EB = elementary body; n = 3; bars indicate SE.
(TIF)

S13 Fig Effect of Retro-2 treatment on the localization of SNX1, SNX2, eGFP-SNX5 and eGFP-SNX6 during *C*. *trachomatis* infection. Confocal IF images showing localization of SNX1, SNX2, eGFP-SNX5 and eGFP-SNX6 during *C*. *trachomatis* L2 infection (MOI 2) at 12 h (SNX1, SNX2 and eGFP-SNX5), 16 h (eGFP-SNX6) and 24 h p.i. treated with indicated concentrations of Retro-2 or DMSO as solvent control. Cells were stained with indicated antibodies; DNA was stained with DAPI (blue). Images show maximum intensity projections of z-stacks. Scale bar, 10 μm; n = 2.
(TIF)

S14 Fig Known inclusion associated proteins by abundance in cell lysate. Previously reported inclusion associated proteins were ranked by their abundance in HeLa cell lysates based on iBAQ intensity of tryptic peptides [22]. Proteins that were not found in the lysate are on the detection limit (rank 8604, n = 5). Positive = proteins that passed the SILAC exclusion approach, Excluded = did not pass the SILAC exclusion approach or were removed by initial filtering of common contaminants. Not in triplicate = proteins detected in the inclusion fraction but not in all experiments. Not found = proteins that were never detected in the inclusion fraction. Too few peptides = proteins that were identified in all three experiments but with only one peptide.
(TIF)

S15 Fig Distribution of protein annotation terms in lysate and inclusion proteomes. Proteins that were reliably found and quantified in the inclusion and the total cell lysate (Lysate: n = 2002; Inclusion: n = 351) were annotated with subcellular localization data from UniprotKB. The percentage of proteins annotated with the indicated term is shown. One protein can have annotations for several organelles.
(TIF)

S1 Text Supplemental experimental procedures with references. (DOCX)

S1 Table Inclusion associated host proteins. Protein ID = Uniprot ID of the first protein of the Majority protein column of a protein group. Majority protein groups identified in Lysates and Inclusion fractions are listed in the respective columns. Relative SD = standard deviation of the indicated percentage/ratio divided by mean (coefficient of variation). PEP Score = Posterior Error Probability as reported by MaxQuant. Inclusion SILAC L/H AVG = average of SILAC ratios in the inclusion fraction. SILAC enrichment analysis set = test performed with three (1) or two (2) SILAC ratios. Log2(iBAQ Enrichment) = log2 transformed iBAQ enrichment score as described in S1 Text; calculation of the relative abundance of the protein in the inclusion fraction relative to its relative abundance in the total cell lysate. Peptides = number of Razor + Unique peptides identified in each experiment. Inclusion Quantification Qualifier = 1 if relative SD below 0.5. Lysate Quantification Qualifier: 0 = quantified on the basis of one experiment; 1 = quantified in all three experiments with two or more peptides and relative SD below 0.5; as (1) but less than two peptides in one experiment; 3 = as 2 but only in found in two of three experiments;— = relative SD above 0.5; N = quantified based on dataset from Nagaraj et al. 2011 [31].
(XLSX)

S2 Table Overview of proteins that associate with the inclusion of *C*. *trachomatis* at 24 h p.i. with references. Trivial names were used as reported in the cited manuscripts. The Uniprot identifier (ID) of the reviewed human protein is shown for each protein except for actin where the exact proteins were not defined. ISO indicates if a highly homologous variant was found.
(DOCX)

S3 Table Gene Ontology enrichment analysis of biological processes. Output of enrichment analysis as obtained from GOrilla [57]. Table legend as supplied by GOrilla: 'P-value' is the enrichment p value computed according to the mHG or HG model. This p value is not corrected for multiple testing of 9401 GO terms. 'FDR q-value' is the correction of the above p value for multiple testing using the Benjamini and Hochberg method. Namely, for the i^th^ term (ranked according to p value) the FDR q-value is (p value * number of GO terms) / i. Enrichment (N, B, n, b) is defined as follows: N—is the total number of genes B—is the total number of genes associated with a specific GO term n—is the number of genes in the top of the user's input list or in the target set when appropriate b—is the number of genes in the intersection. Enrichment = (b/n) / (B/N). Genes: For each GO term you can see the list of associated genes that appear in the optimal top of the list. Each gene name is specified by gene symbol followed by a short description of the gene.
(XLSX)

## References

[1] Senior K (2012) Chlamydia: a much underestimated STI. Lancet Infect Dis 12: 517–518. 22930827 10.1016/s1473-3099(12)70161-5

[2] Moulder JW (1991) Interaction of chlamydiae and host cells in vitro. Microbiol Rev 55: 143–190. 2030670 10.1128/mr.55.1.143-190.1991 PMC372804

[3] Heinzen RA, Hackstadt T (1997) The Chlamydia trachomatis parasitophorous vacuolar membrane is not passively permeable to low-molecular-weight compounds. Infect Immun 65: 1088–1094. 9038320 10.1128/iai.65.3.1088-1094.1997 PMC175092

[4] Saka HA, Thompson JW, Chen YS, Kumar Y, Dubois LG, et al (2011) Quantitative proteomics reveals metabolic and pathogenic properties of Chlamydia trachomatis developmental forms. Mol Microbiol 82: 1185–1203. 10.1111/j.1365-2958.2011.07877.x 22014092 PMC3225693

[5] Seaman MN (2012) The retromer complex—endosomal protein recycling and beyond. J Cell Sci 125: 4693–4702. 10.1242/jcs.103440 23148298 PMC3517092

[6] Cullen PJ, Korswagen HC (2012) Sorting nexins provide diversity for retromer-dependent trafficking events. Nat Cell Biol 14: 29–37. 10.1038/ncb2374 22193161 PMC3613977

[7] Worby CA, Dixon JE (2002) Sorting out the cellular functions of sorting nexins. Nat Rev Mol Cell Biol 3: 919–931. 12461558 10.1038/nrm974

[8] Niu Y, Zhang C, Sun Z, Hong Z, Li K, et al (2013) PtdIns(4)P regulates retromer-motor interaction to facilitate dynein-cargo dissociation at the trans-Golgi network. Nat Cell Biol 15: 417–429. 10.1038/ncb2710 23524952

[9] Hong Z, Yang Y, Zhang C, Niu Y, Li K, et al (2009) The retromer component SNX6 interacts with dynactin p150(Glued) and mediates endosome-to-TGN transport. Cell Res 19: 1334–1349. 10.1038/cr.2009.130 19935774

[10] Garin J, Diez R, Kieffer S, Dermine JF, Duclos S, et al (2001) The phagosome proteome: insight into phagosome functions. J Cell Biol 152: 165–180. 11149929 10.1083/jcb.152.1.165 PMC2193653

[11] Gotthardt D, Warnatz HJ, Henschel O, Bruckert F, Schleicher M, et al (2002) High-resolution dissection of phagosome maturation reveals distinct membrane trafficking phases. Mol Biol Cell 13: 3508–3520. 12388753 10.1091/mbc.E02-04-0206 PMC129962

[12] Gotthardt D, Blancheteau V, Bosserhoff A, Ruppert T, Delorenzi M, et al (2006) Proteomics fingerprinting of phagosome maturation and evidence for the role of a Galpha during uptake. Mol Cell Proteomics 5: 2228–2243. 16926386 10.1074/mcp.M600113-MCP200

[13] Sturgill-Koszycki S, Haddix PL, Russell DG (1997) The interaction between Mycobacterium and the macrophage analyzed by two-dimensional polyacrylamide gel electrophoresis. Electrophoresis 18: 2558–2565. 9527485 10.1002/elps.1150181411

[14] Mills SD, Finlay BB (1998) Isolation and characterization of Salmonella typhimurium and Yersinia pseudotuberculosis-containing phagosomes from infected mouse macrophages: Y. pseudotuberculosis traffics to terminal lysosomes where they are degraded. Eur J Cell Biol 77: 35–47. 9808287 10.1016/S0171-9335(98)80100-3

[15] Luhrmann A, Haas A (2000) A method to purify bacteria-containing phagosomes from infected macrophages. Methods Cell Sci 22: 329–341. 11549946 10.1023/a:1017963401560

[16] Urwyler S, Nyfeler Y, Ragaz C, Lee H, Mueller LN, et al (2009) Proteome analysis of Legionella vacuoles purified by magnetic immunoseparation reveals secretory and endosomal GTPases. Traffic 10: 76–87. 10.1111/j.1600-0854.2008.00851.x 18980612

[17] Shevchuk O, Batzilla C, Hagele S, Kusch H, Engelmann S, et al (2009) Proteomic analysis of Legionella-containing phagosomes isolated from Dictyostelium. Int J Med Microbiol 299: 489–508. 10.1016/j.ijmm.2009.03.006 19482547

[18] Rockey DD, Heinzen RA, Hackstadt T (1995) Cloning and characterization of a Chlamydia psittaci gene coding for a protein localized in the inclusion membrane of infected cells. Mol Microbiol 15: 617–626. 7783634 10.1111/j.1365-2958.1995.tb02371.x

[19] Rzomp KA, Scholtes LD, Briggs BJ, Whittaker GR, Scidmore MA (2003) Rab GTPases are recruited to chlamydial inclusions in both a species-dependent and species-independent manner. Infect Immun 71: 5855–5870. 14500507 10.1128/IAI.71.10.5855-5870.2003 PMC201052

[20] Ong SE, Blagoev B, Kratchmarova I, Kristensen DB, Steen H, et al (2002) Stable isotope labeling by amino acids in cell culture, SILAC, as a simple and accurate approach to expression proteomics. Mol Cell Proteomics 1: 376–386. 12118079 10.1074/mcp.m200025-mcp200

[21] Schwanhausser B, Busse D, Li N, Dittmar G, Schuchhardt J, et al (2011) Global quantification of mammalian gene expression control. Nature 473: 337–342. 10.1038/nature10098 21593866

[22] Nagaraj N, Wisniewski JR, Geiger T, Cox J, Kircher M, et al (2011) Deep proteome and transcriptome mapping of a human cancer cell line. Mol Syst Biol 7: 548 10.1038/msb.2011.81 22068331 PMC3261714

[23] UniProt C (2014) Activities at the Universal Protein Resource (UniProt). Nucleic Acids Res 42: D191–198. 10.1093/nar/gkt1140 24253303 PMC3965022

[24] Capmany A, Damiani MT (2010) Chlamydia trachomatis intercepts Golgi-derived sphingolipids through a Rab14-mediated transport required for bacterial development and replication. PLoS One 5: e14084 10.1371/journal.pone.0014084 21124879 PMC2989924

[25] Derre I, Swiss R, Agaisse H (2011) The lipid transfer protein CERT interacts with the Chlamydia inclusion protein IncD and participates to ER-Chlamydia inclusion membrane contact sites. PLoS Pathog 7: e1002092 10.1371/journal.ppat.1002092 21731489 PMC3121800

[26] Elwell CA, Jiang S, Kim JH, Lee A, Wittmann T, et al (2011) Chlamydia trachomatis co-opts GBF1 and CERT to acquire host sphingomyelin for distinct roles during intracellular development. PLoS Pathog 7: e1002198 10.1371/journal.ppat.1002198 21909260 PMC3164637

[27] Scidmore MA, Hackstadt T (2001) Mammalian 14-3-3beta associates with the Chlamydia trachomatis inclusion membrane via its interaction with IncG. Mol Microbiol 39: 1638–1650. 11260479 10.1046/j.1365-2958.2001.02355.x

[28] Franceschini A, Szklarczyk D, Frankild S, Kuhn M, Simonovic M, et al (2013) STRING v9.1: protein-protein interaction networks, with increased coverage and integration. Nucleic Acids Res 41: D808–815. 10.1093/nar/gks1094 23203871 PMC3531103

[29] Stechmann B, Bai SK, Gobbo E, Lopez R, Merer G, et al (2010) Inhibition of retrograde transport protects mice from lethal ricin challenge. Cell 141: 231–242. 10.1016/j.cell.2010.01.043 20403321

[30] Hoffmann C, Finsel I, Otto A, Pfaffinger G, Rothmeier E, et al. (2013) Functional analysis of novel Rab GTPases identified in the proteome of purified Legionella-containing vacuoles from macrophages. Cell Microbiol. 10.1111/cmi.12256 24373249

[31] Chiappino ML, Dawson C, Schachter J, Nichols BA (1995) Cytochemical localization of glycogen in Chlamydia trachomatis inclusions. J Bacteriol 177: 5358–5363. 7545158 10.1128/jb.177.18.5358-5363.1995 PMC177334

[32] Cocchiaro JL, Kumar Y, Fischer ER, Hackstadt T, Valdivia RH (2008) Cytoplasmic lipid droplets are translocated into the lumen of the Chlamydia trachomatis parasitophorous vacuole. Proc Natl Acad Sci U S A 105: 9379–9384. 10.1073/pnas.0712241105 18591669 PMC2453745

[33] Campbell-Valois FX, Trost M, Chemali M, Dill BD, Laplante A, et al (2012) Quantitative proteomics reveals that only a subset of the endoplasmic reticulum contributes to the phagosome. Mol Cell Proteomics 11: M111 016378. 10.1074/mcp.M111.016378 PMC3394953 22427703

[34] Fields KA, Hackstadt T (2002) The chlamydial inclusion: escape from the endocytic pathway. Annu Rev Cell Dev Biol 18: 221–245. 12142274 10.1146/annurev.cellbio.18.012502.105845

[35] Stephens RS, Kalman S, Lammel C, Fan J, Marathe R, et al (1998) Genome sequence of an obligate intracellular pathogen of humans: Chlamydia trachomatis. Science 282: 754–759. 9784136 10.1126/science.282.5389.754

[36] Nelson CD, Carney DW, Derdowski A, Lipovsky A, Gee GV, et al (2013) A retrograde trafficking inhibitor of ricin and Shiga-like toxins inhibits infection of cells by human and monkey polyomaviruses. MBio 4: e00729–00713. 10.1128/mBio.00729-13 24222489 PMC3892778

[37] Lipovsky A, Popa A, Pimienta G, Wyler M, Bhan A, et al (2013) Genome-wide siRNA screen identifies the retromer as a cellular entry factor for human papillomavirus. Proc Natl Acad Sci U S A 110: 7452–7457. 10.1073/pnas.1302164110 23569269 PMC3645514

[38] Sandvig K, Garred O, Prydz K, Kozlov JV, Hansen SH, et al (1992) Retrograde transport of endocytosed Shiga toxin to the endoplasmic reticulum. Nature 358: 510–512. 1641040 10.1038/358510a0

[39] Bujny MV, Ewels PA, Humphrey S, Attar N, Jepson MA, et al (2008) Sorting nexin-1 defines an early phase of Salmonella-containing vacuole-remodeling during Salmonella infection. J Cell Sci 121: 2027–2036. 10.1242/jcs.018432 18505799

[40] Braun V, Wong A, Landekic M, Hong WJ, Grinstein S, et al (2010) Sorting nexin 3 (SNX3) is a component of a tubular endosomal network induced by Salmonella and involved in maturation of the Salmonella-containing vacuole. Cell Microbiol 12: 1352–1367. 10.1111/j.1462-5822.2010.01476.x 20482551

[41] Peter BJ, Kent HM, Mills IG, Vallis Y, Butler PJ, et al (2004) BAR domains as sensors of membrane curvature: the amphiphysin BAR structure. Science 303: 495–499. 14645856 10.1126/science.1092586

[42] Carlton J, Bujny M, Peter BJ, Oorschot VM, Rutherford A, et al (2004) Sorting nexin-1 mediates tubular endosome-to-TGN transport through coincidence sensing of high- curvature membranes and 3-phosphoinositides. Curr Biol 14: 1791–1800. 15498486 10.1016/j.cub.2004.09.077

[43] Moorhead AM, Jung JY, Smirnov A, Kaufer S, Scidmore MA (2010) Multiple host proteins that function in phosphatidylinositol-4-phosphate metabolism are recruited to the chlamydial inclusion. Infect Immun 78: 1990–2007. 10.1128/IAI.01340-09 20231409 PMC2863499

[44] Swarbrick JD, Shaw DJ, Chhabra S, Ghai R, Valkov E, et al (2011) VPS29 is not an active metallo-phosphatase but is a rigid scaffold required for retromer interaction with accessory proteins. PLoS One 6: e20420 10.1371/journal.pone.0020420 21629666 PMC3101248

[45] Nisar S, Kelly E, Cullen PJ, Mundell SJ (2010) Regulation of P2Y1 receptor traffic by sorting Nexin 1 is retromer independent. Traffic 11: 508–519. 10.1111/j.1600-0854.2010.01035.x 20070609

[46] Prosser DC, Tran D, Schooley A, Wendland B, Ngsee JK (2010) A novel, retromer-independent role for sorting nexins 1 and 2 in RhoG-dependent membrane remodeling. Traffic 11: 1347–1362. 10.1111/j.1600-0854.2010.01100.x 20604901

[47] Chua CE, Lim YS, Lee MG, Tang BL (2012) Non-classical membrane trafficking processes galore. J Cell Physiol 227: 3722–3730. 10.1002/jcp.24082 22378347

[48] Tan X, Sun Y, Thapa N, Liao Y, Hedman AC, et al (2015) LAPTM4B is a PtdIns(4,5)P2 effector that regulates EGFR signaling, lysosomal sorting, and degradation. EMBO J 34: 475–490. 10.15252/embj.201489425 25588945 PMC4331002

[49] Seaman MN (2004) Cargo-selective endosomal sorting for retrieval to the Golgi requires retromer. J Cell Biol 165: 111–122. 15078902 10.1083/jcb.200312034 PMC2172078

[50] Patel AL, Chen X, Wood ST, Stuart ES, Arcaro KF, et al (2014) Activation of epidermal growth factor receptor is required for Chlamydia trachomatis development. BMC Microbiol 14: 277 10.1186/s12866-014-0277-4 25471819 PMC4269859

[51] Zhou B, Yun EY, Ray L, You J, Ip YT, et al (2014) Retromer promotes immune quiescence by suppressing Spatzle-Toll pathway in Drosophila. J Cell Physiol 229: 512–520. 10.1002/jcp.24472 24343480 PMC4346153

[52] Johannes L, Popoff V (2008) Tracing the retrograde route in protein trafficking. Cell 135: 1175–1187. 10.1016/j.cell.2008.12.009 19109890

[53] Moore ER, Mead DJ, Dooley CA, Sager J, Hackstadt T (2011) The trans-Golgi SNARE syntaxin 6 is recruited to the chlamydial inclusion membrane. Microbiology 157: 830–838. 10.1099/mic.0.045856-0 21109560 PMC3081085

[54] Kabeiseman EJ, Cichos K, Hackstadt T, Lucas A, Moore ER (2013) Vesicle-associated membrane protein 4 and syntaxin 6 interactions at the chlamydial inclusion. Infect Immun 81: 3326–3337. 10.1128/IAI.00584-13 23798538 PMC3754233

[55] Banhart S, Saied EM, Martini A, Koch S, Aeberhard L, et al. (2014) Improved plaque assay identifies a novel anti-Chlamydia ceramide derivative with altered intracellular localization. Antimicrob Agents Chemother. 10.1128/AAC.03457-14 PMC4135853 25001308

[56] Cox J, Mann M (2008) MaxQuant enables high peptide identification rates, individualized p.p.b.-range mass accuracies and proteome-wide protein quantification. Nat Biotechnol 26: 1367–1372. 10.1038/nbt.1511 19029910

[57] Eden E, Navon R, Steinfeld I, Lipson D, Yakhini Z (2009) GOrilla: a tool for discovery and visualization of enriched GO terms in ranked gene lists. BMC Bioinformatics 10: 48 10.1186/1471-2105-10-48 19192299 PMC2644678

