# Supplementary figures and images for: The Proteome of the Isolated Chlamydia trachomatis Containing Vacuole Reveals a Complex Trafficking Platform Enriched for Retromer Components
Source: PLoS Pathog. 2015 Jun 4;11(6):e1004883. doi: 10.1371/journal.ppat.1004883 (PMC4456400; doi:10.1371/journal.ppat.1004883)

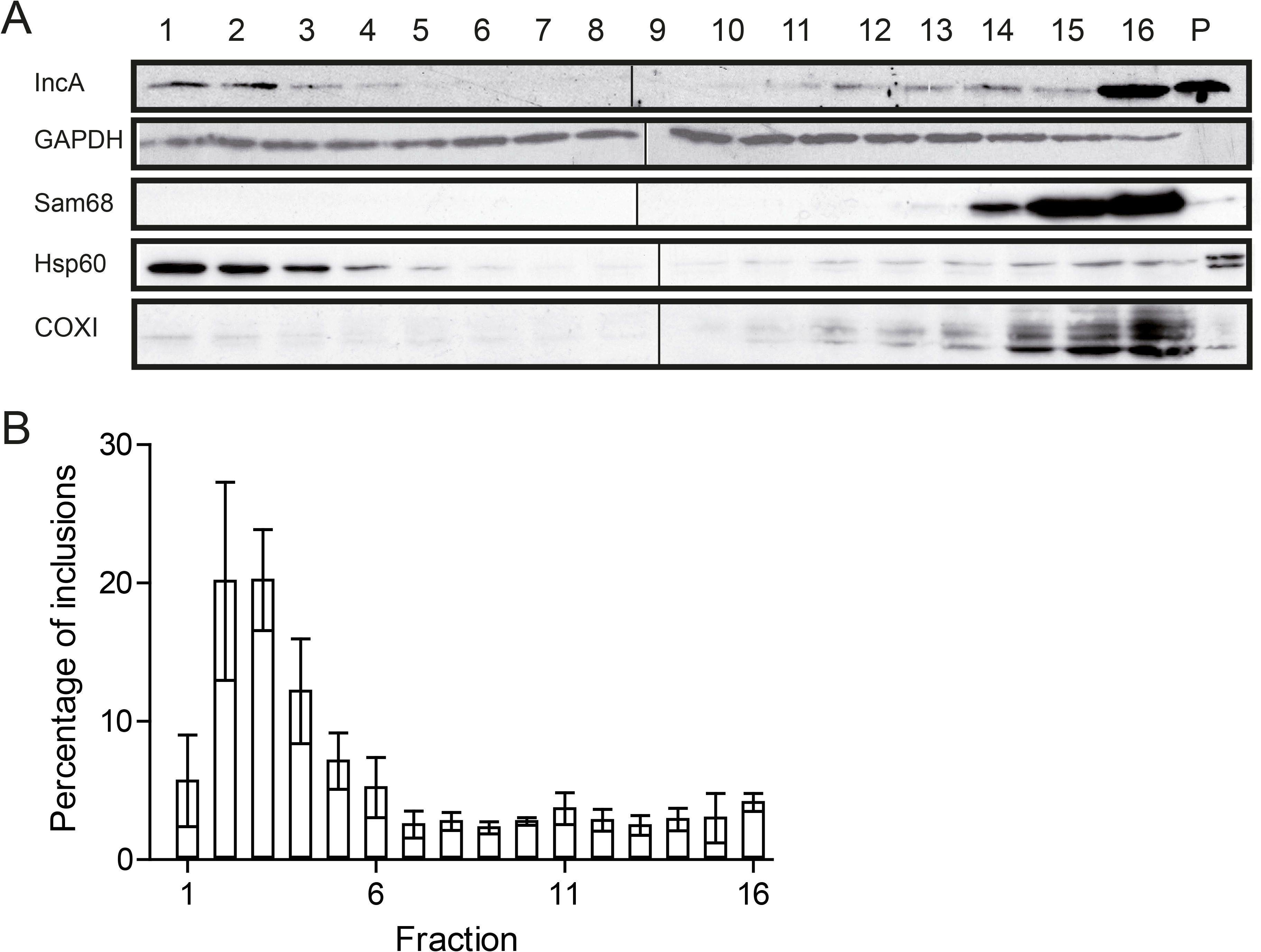

Supplement: S1 Fig — A) 6 x 10^7^ HeLa cells were infected with C. trachomatis L2 for 24 h (MOI 2). Cells were lysed in a ball homogenizer (16 μm clearance, 13 strokes) and subsequently fractionated on an in-situ formed 33% Percoll gradient in HSMG buffer. The gradient was fractionated into 16 fractions of equal volume (fraction 1: bottom, fraction 16: top). Fractions 1–4 were pooled, diluted in HSMG and washed twice (P). Equal volumes of each fraction (1–16) or concentrated washed inclusions (P) were prepared, separated on a 10% SDS-PAGE gel, western blotted and probed with specific antibodies against the indicated proteins. B) The majority of intact inclusions sediments to high density fractions in a Percoll gradient. Intact inclusions were counted for each fraction of a gradient prepared as described in A) but MOI 3, and the percentage was plotted. Error bars indicate standard deviation of three independent replicates. (TIF) [file ppat.1004883.s001.tif]

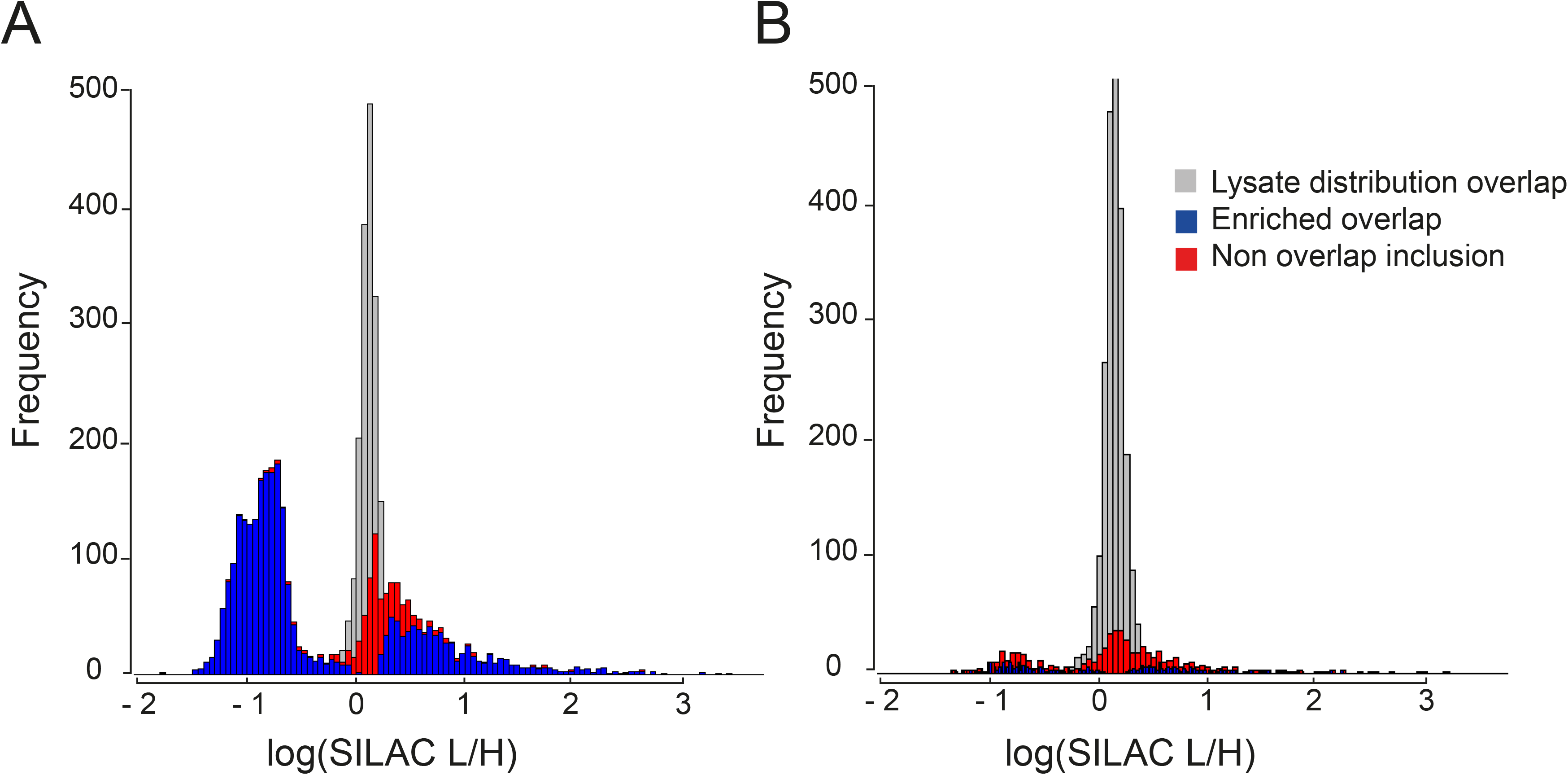

Supplement: S2 Fig — Proteins were tested for enrichment in the inclusion fraction. The graph shows a bar diagram with the empirical distribution of the logarithm of the SILAC ratios of proteins that were found in both the inclusion and lysate fraction. The grey bars indicate the SILAC ratios of proteins found in the lysate which overlap with inclusion proteins, blue bars show proteins that are differentially enriched in the inclusion fraction. Red bars show proteins which were only found in inclusion dataset. Proteins enriched in the inclusion fraction are expected to have positive (L/H) SILAC ratios. A) Proteins of the inclusion fraction which show three SILAC ratios (blue and red) B) Proteins of the inclusion fraction which only show two SILAC ratios (blue and red). The highest bar was capped at 500. More proteins were used for the empirical lysate distribution compared to A, because the overlap for both proteins with two and three SILAC ratios was used. (TIF) [file ppat.1004883.s002.tif]

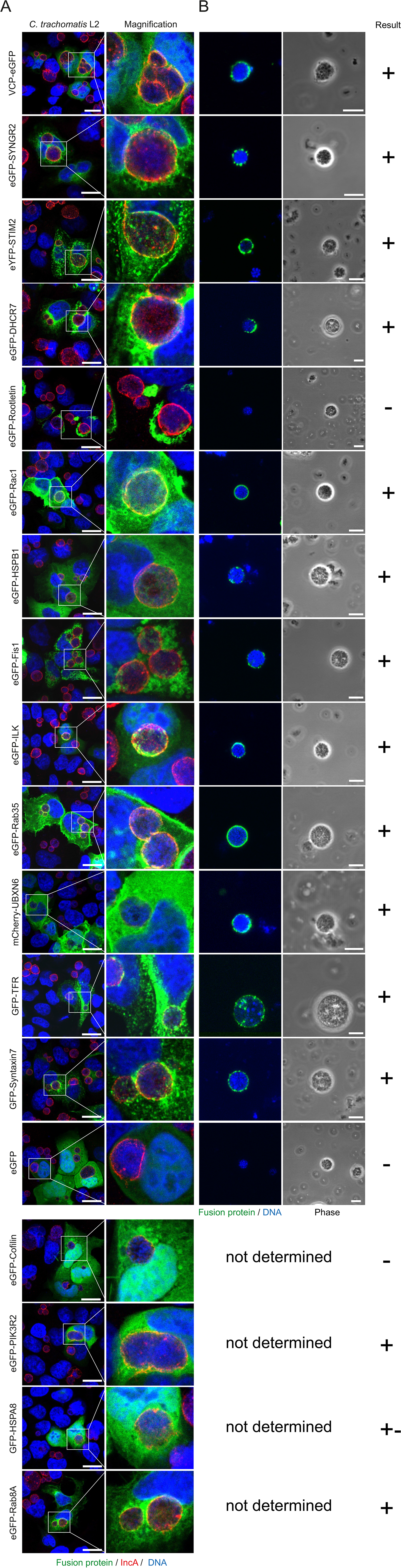

Supplement: S3 Fig — A) IF images showing HeLa cells expressing the indicated fluorescent fusion proteins (green), infected with C. trachomatis L2 (MOI 2). Cells were fixed 24 h p.i. with 2% PFA and stained for IncA (inclusion membrane, red) and DNA (DAPI, blue). Scale bar, 20 μm. B) Validation by purified inclusions in live cell microscopy. Inclusions were gradient purified from cells expressing the indicated fusion protein using a small scale protocol and analyzed by LSCM, DNA was stained with DAPI. Scale bar, 5 μm. The “results” column indicates whether a protein was considered to be positively validated (+), not inclusion associated (-) or ambiguous (+-). (TIF) [file ppat.1004883.s003.tif]

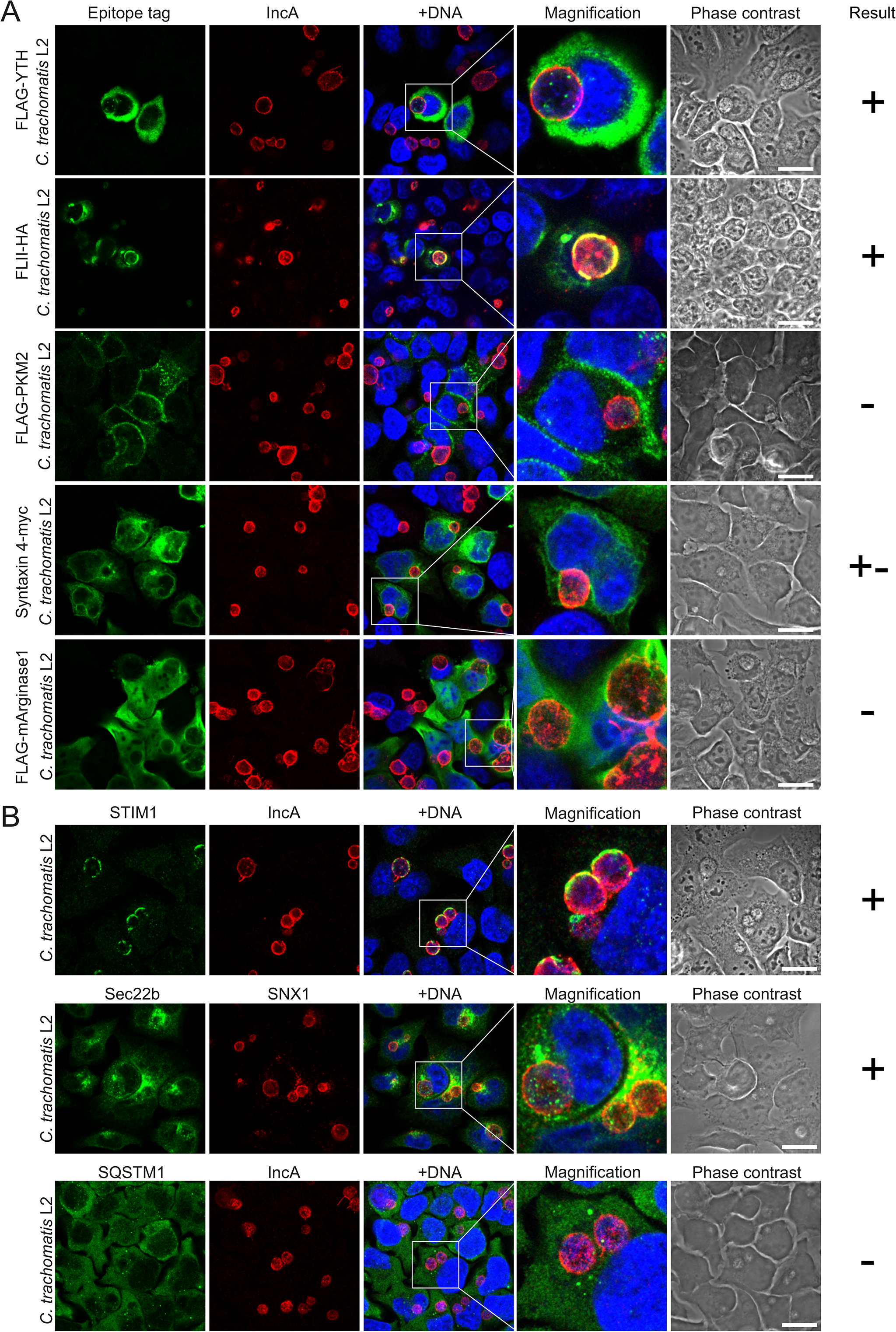

Supplement: S4 Fig — A) Confocal immunofluorescence images showing localization of ectopically expressed epitope tagged proteins in C. trachomatis L2 infected cells. HeLa cells were transfected, infected with MOI 2 and fixed at 24 h p.i. with 2% PFA in PBS, except for FLII-HA which was fixed with ice cold methanol. B) Confocal immunofluorescence images showing localization of endogenous proteins of interest. HeLa cells were infected with MOI 2 and fixed at 24 h p.i. with 2% PFA in PBS. A) and B) Cells were stained with indicated antibodies; DNA was stained with DAPI (blue). The results column indicates whether a protein was considered to be positively validated (+), not inclusion associated (-) or ambiguous (+-). Scale bar, 20 μm. (TIF) [file ppat.1004883.s004.tif]

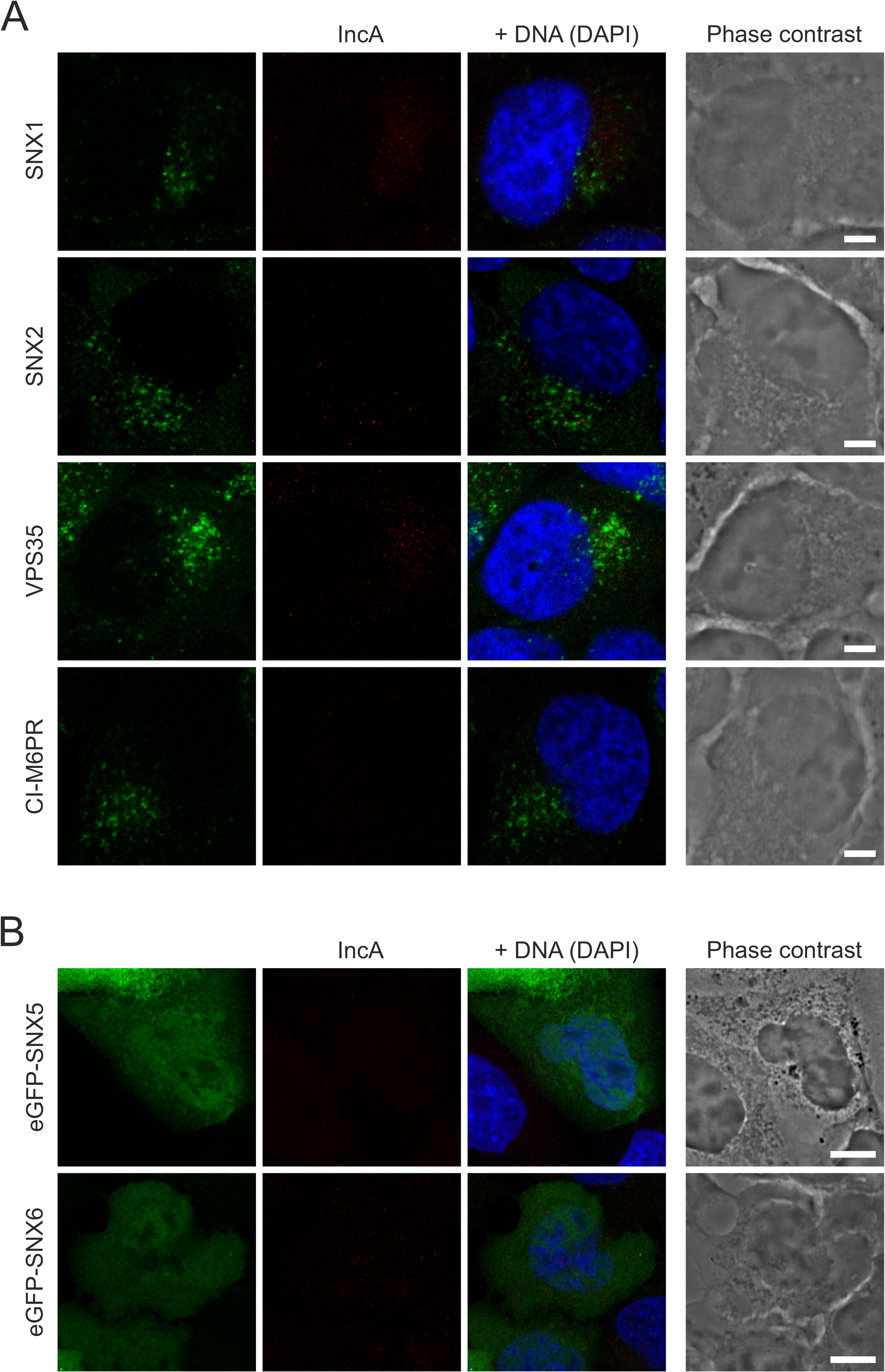

Supplement: S5 Fig — A) Confocal immunofluorescence images showing localization of retromer components in uninfected control cells. HeLa cells were fixed and stained with indicated antibodies; DNA was stained with DAPI (blue). Scale bar, 5 μm; n = 3. B) Confocal immunofluorescence images showing localization of eGFP fusion proteins of human SNX5 and SNX6 in uninfected control cells. HeLa cells were transfected, fixed and stained with indicated antibodies; DNA was stained with DAPI (blue). Scale bar, 10 μm; n = 2. (TIF) [file ppat.1004883.s005.tif]

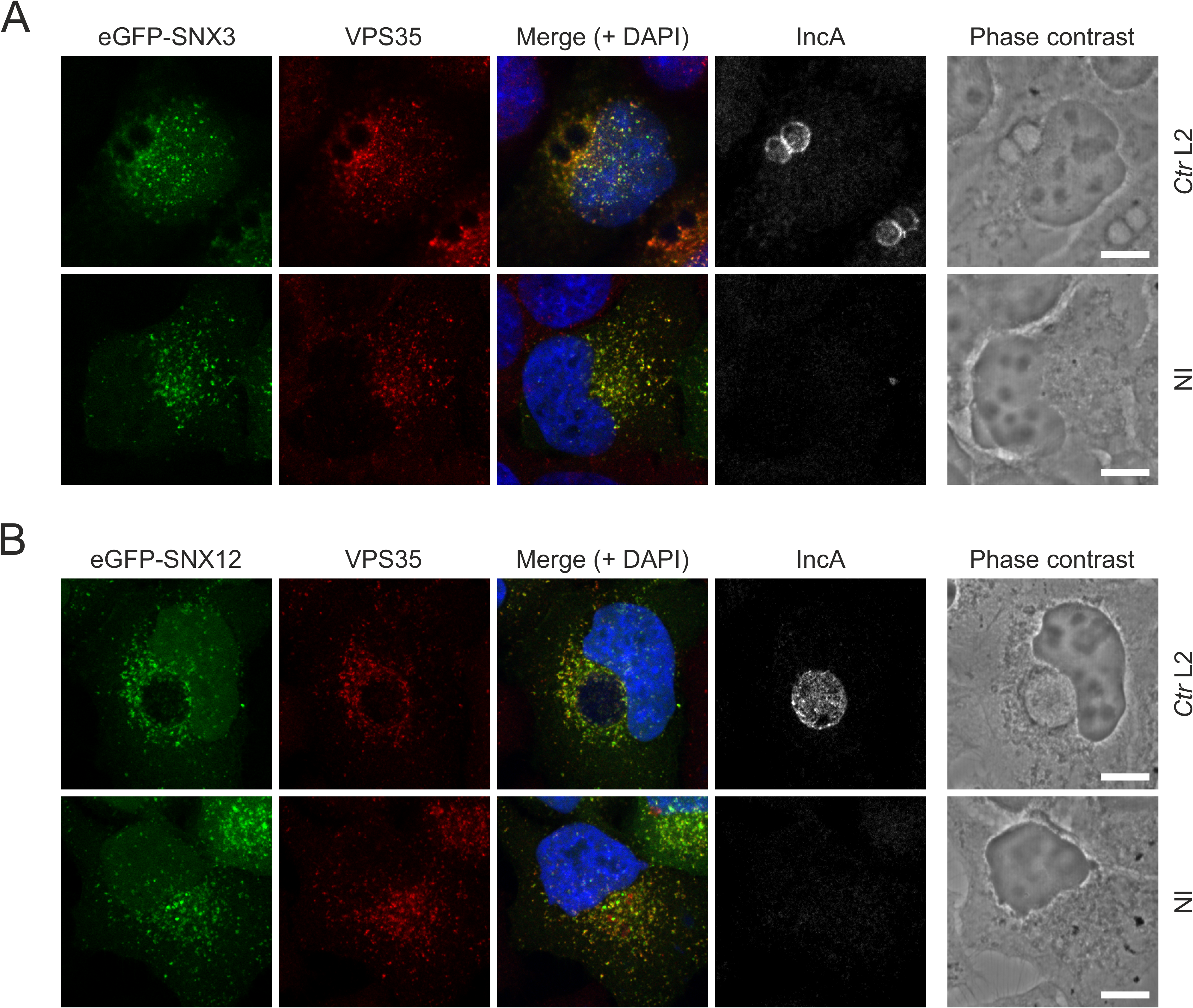

Supplement: S6 Fig — Confocal immunofluorescence images showing co-localization of A) eGFP-SNX3 with endogenous VPS35 and B) eGFP-SNX12 with endogenous VPS35 in C. trachomatis L2 infected (Ctr L2, MOI 2) and uninfected (NI) HeLa cells. HeLa cells were infected 4 h prior to transfection, fixed at 24 h p.i. and stained with indicated antibodies; DNA was stained with DAPI (blue). Scale bar, 10 μm; n = 2. (TIF) [file ppat.1004883.s006.tif]

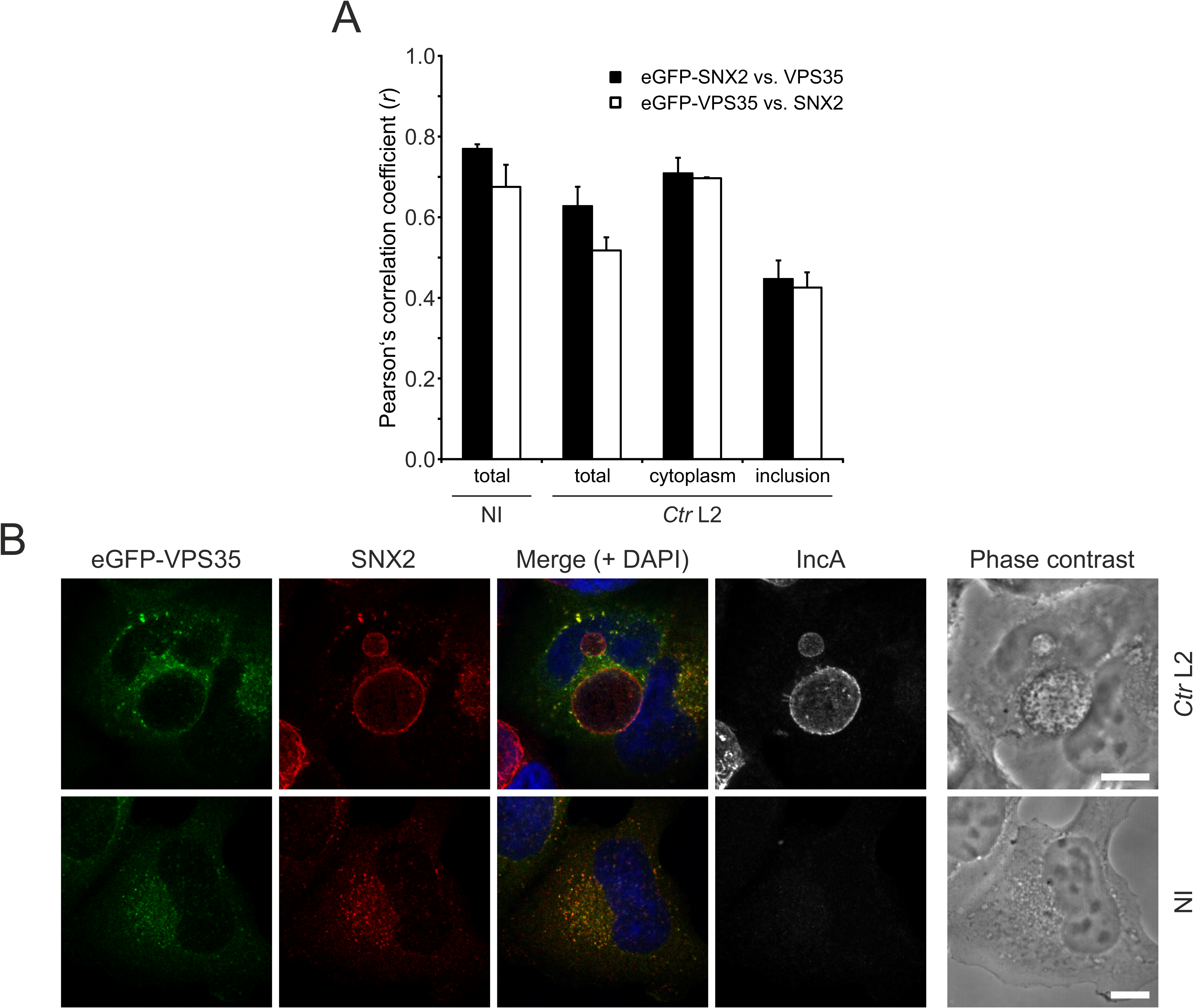

Supplement: S7 Fig — A) Quantification of SNX2/VPS35 co-localization indicating Pearson’s correlation coefficient (r) in C. trachomatis L2 infected (Ctr L2, MOI 2) infected and uninfected cells either expressing eGFP-SNX2 or VPS35-eGFP. Correlation of the two signals was analyzed and quantified using ZEN 2010 software (Zeiss) in either the complete cell area (total), the cytoplasmic area of infected cells excluding the inclusion (cytoplasm) or directly at the inclusion (inclusion) (n = 2; error bars, SE). B) Confocal immunofluorescence images showing co-localization of eGFP-VPS35 fusion protein with endogenous SNX2 in C. trachomatis L2 infected (Ctr L2, MOI 2) and uninfected (NI) HeLa cells. HeLa cells were infected 4 h prior to transfection, fixed at 24 h p.i. and stained with indicated antibodies; DNA was stained with DAPI (blue). Scale bar, 10 μm; n = 2. (TIF) [file ppat.1004883.s007.tif]

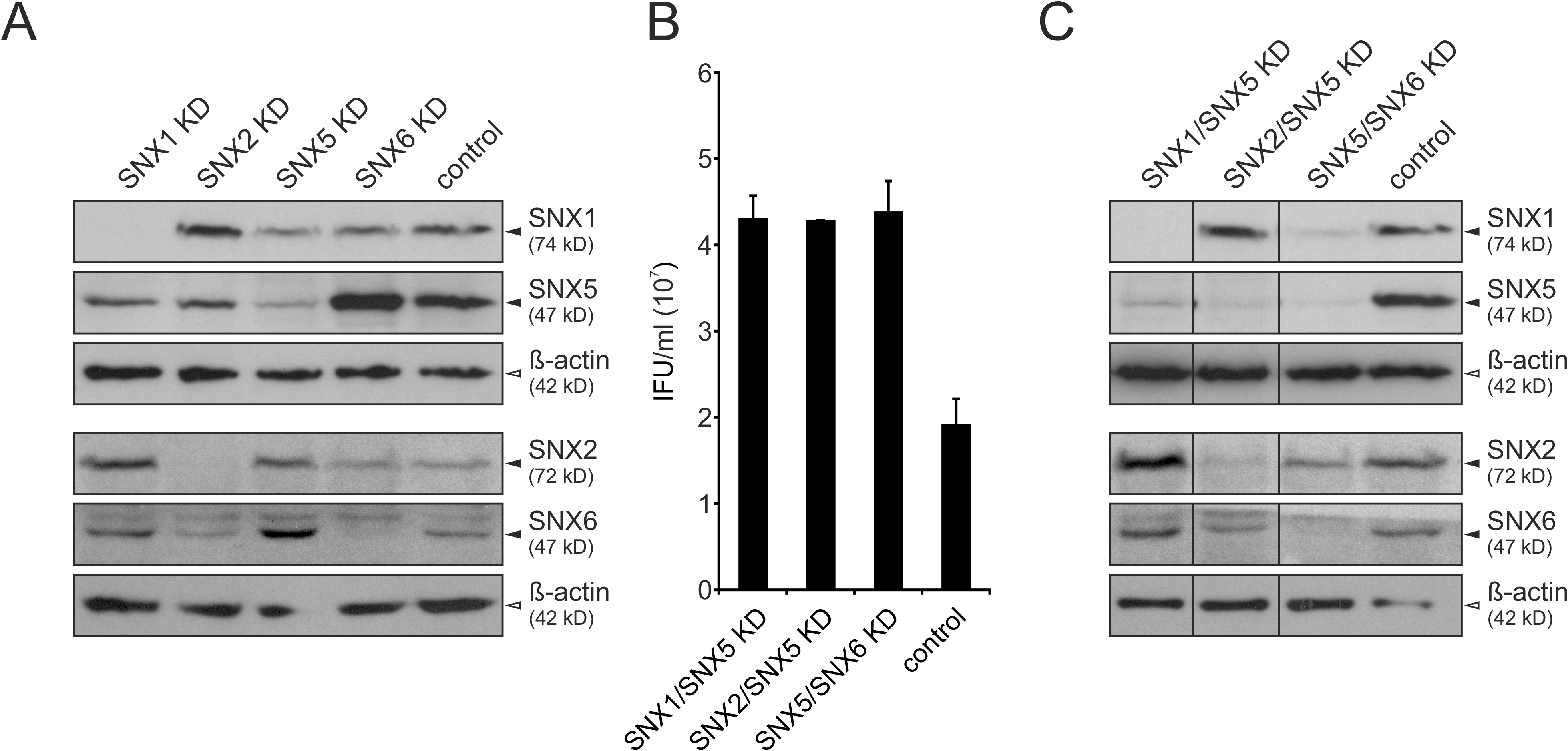

Supplement: S8 Fig — A) Immunoblot analysis of single siRNA knockdowns of retromer components in C. trachomatis L2 infected (Ctr L2, MOI 0.5) HeLa cells. n = 3. B) Reinfection assay assessing the effect of combinational SNXs knockdown on infectious progeny formation 48 h p.i. (n = 3; error bars, SE). C) Western blot analysis of combinational siRNA knockdowns of retromer components in C. trachomatis L2 infected (Ctr L2, MOI 0.5) HeLa cells. β-actin was used as loading control; n = 3. (TIF) [file ppat.1004883.s008.tif]

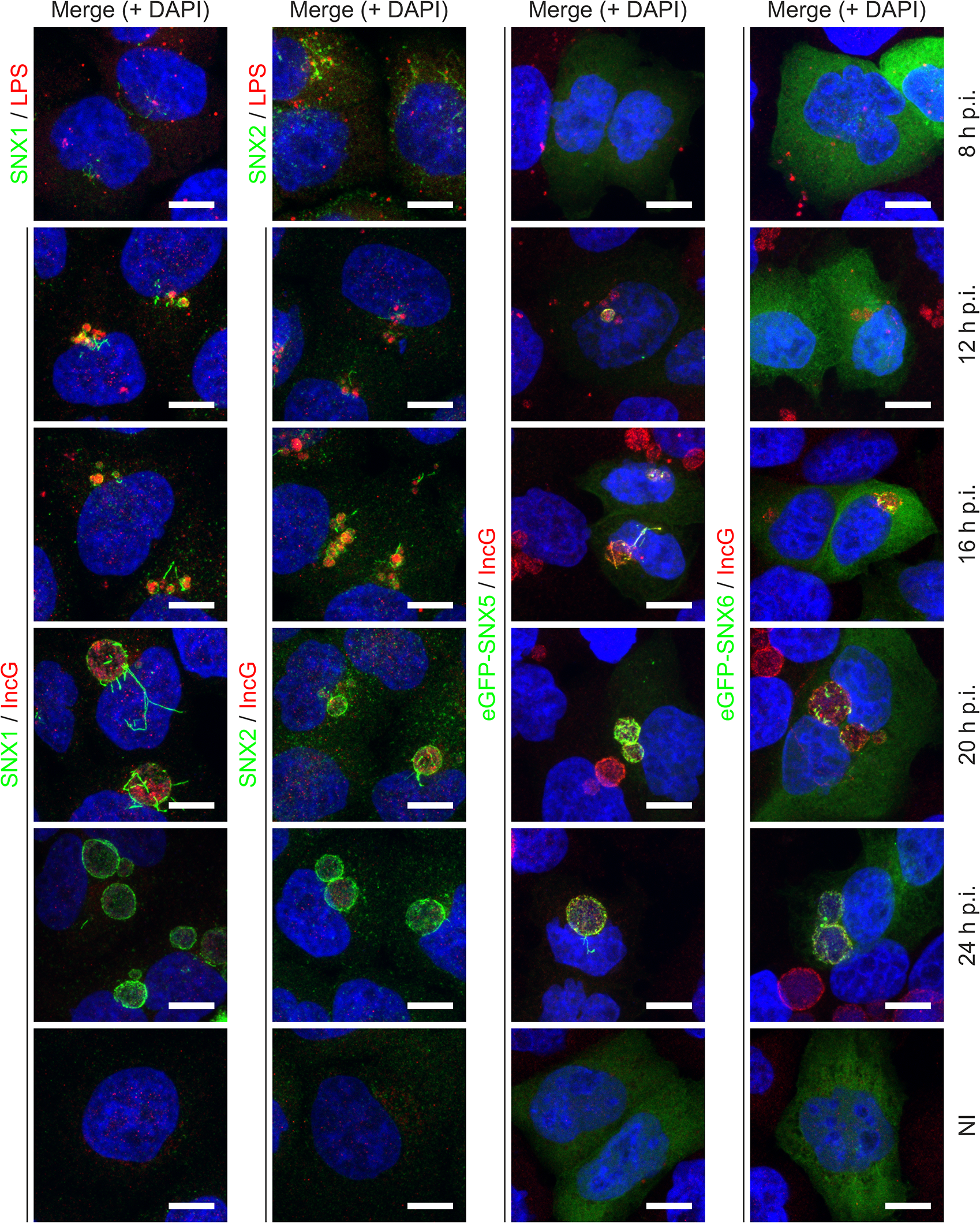

Supplement: S9 Fig — Confocal IF images showing localization of SNX1, SNX2, eGFP-SNX5 and eGFP-SNX6 during C. trachomatis L2 infection (MOI 2) at 8 h, 12 h, 16 h, 20 h and 24 h p.i. and in uninfected HeLa cells. Cells were stained with indicated antibodies; DNA was stained with DAPI (blue). Images show maximum intensity projections of z-stacks. Scale bar, 10 μm; n = 2. (TIF) [file ppat.1004883.s009.tif]

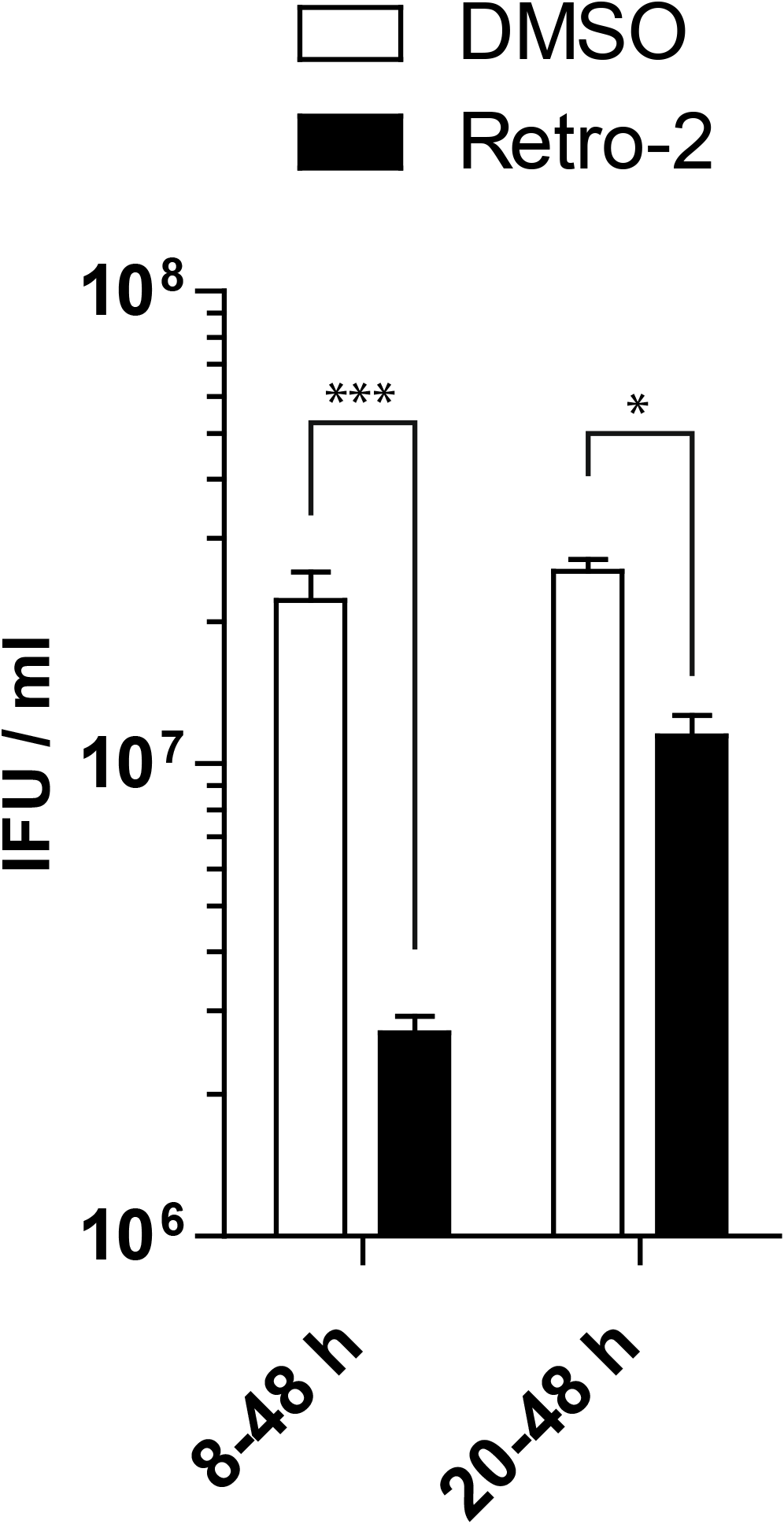

Supplement: S10 Fig — Reinfection assays assessing the effect of Retro-2 on infectious progeny formation. HeLa cells were infected with C. trachomatis L2 (MOI 2) and, at 8 h p.i., or 24 h p.i., cells were either treated with indicated concentrations of Retro-2 or DMSO. Cells were harvested and inclusion forming units (IFU) per ml were determined at 48 h p.i. (n = 3; error bars, SE; *** indicates p value < 0.005). (TIF) [file ppat.1004883.s010.tif]

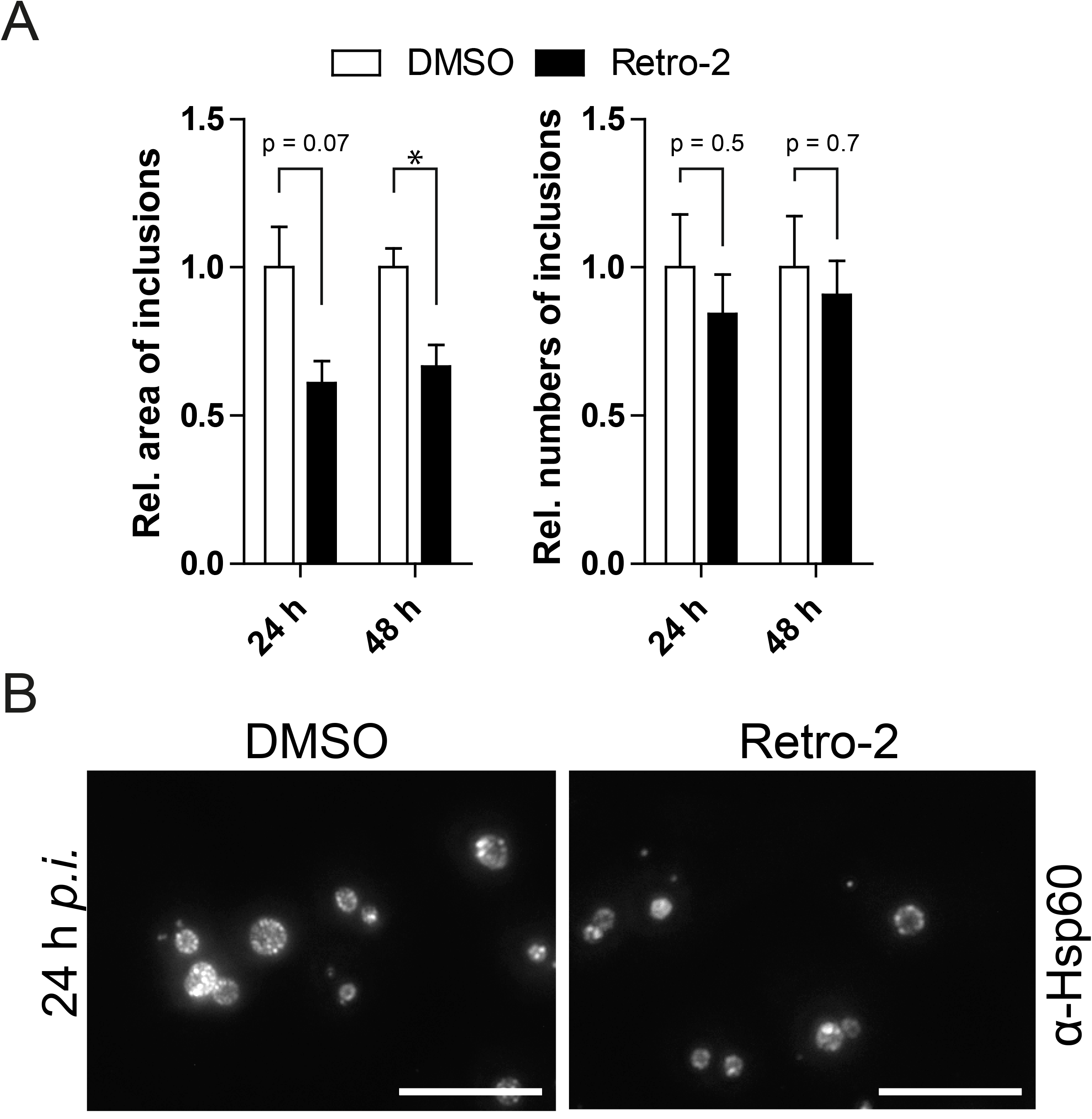

Supplement: S11 Fig — The size and number of inclusions was assessed by immunofluorescence microscopy. HeLa cells were infected with C. trachomatis (MOI 0.5). Retro-2 or DMSO was added at 8 h p.i. Cells were fixed with 2% PFA in PBS at the indicated time point. Immunostaining was performed against bacterial Hsp60 and epifluorescence microscopy pictures were randomly taken at an AxioVert40 inverted microscope. A) A script in ImageJ software was used to count the relative numbers and measure the area of inclusions. Asterisk indicates a p value below 0.05. B) Representative image of Retro-2 and DMSO treated cells at 24 h p.i. Scale bar = 100 μm. (TIF) [file ppat.1004883.s011.tif]

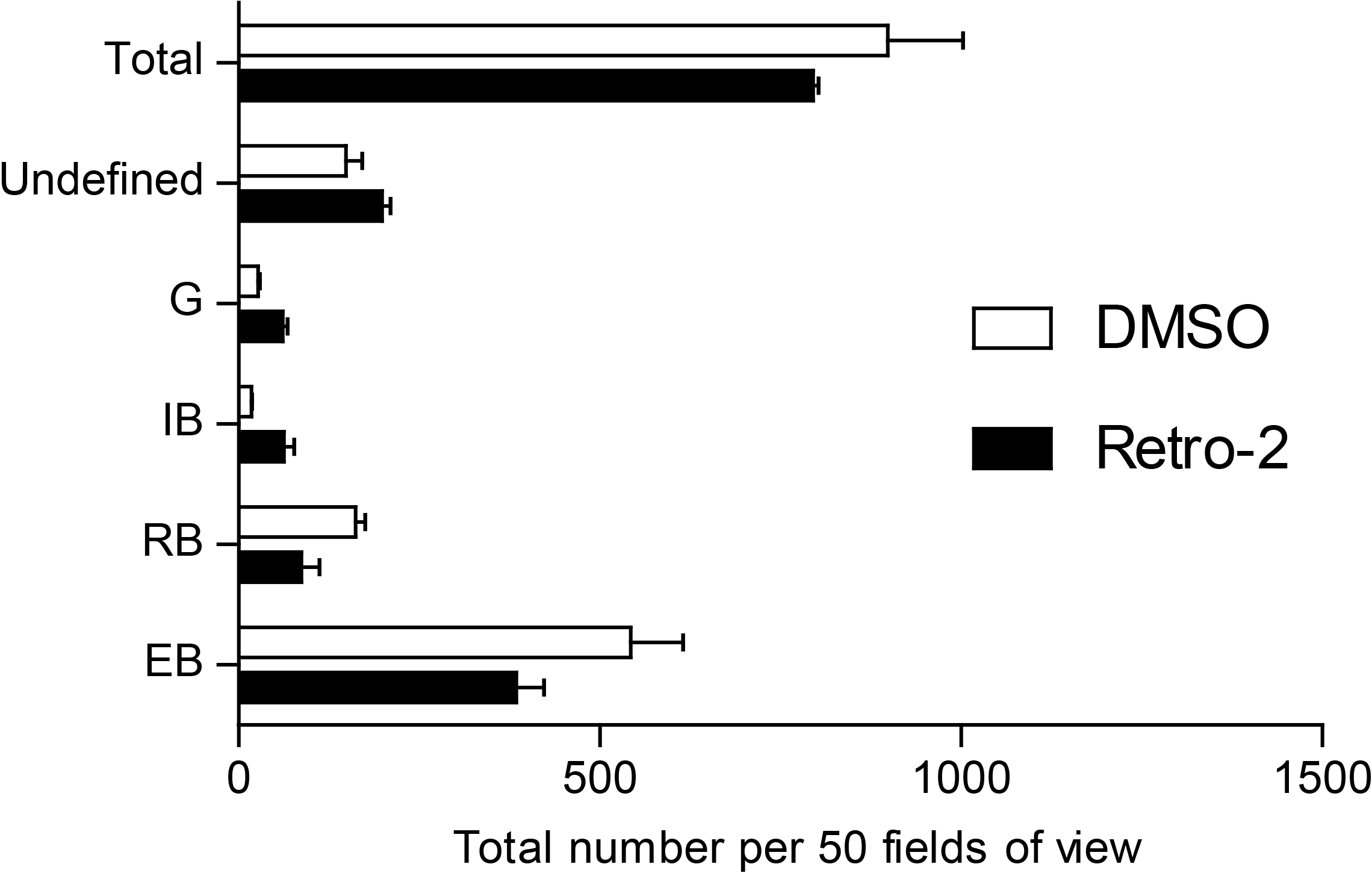

Supplement: S12 Fig — HeLa cells were infected for 48 h with C. trachomatis L2 (MOI 2). The cells were treated with 20 μM Retro-2 at 8 h p.i. or mock treated with DMSO. Cells were pelleted and fixed with glutaraldehyde before processing for TEM. Randomized images were taken from slices and the images were analyzed by eye for the distribution of different morphologies of C. trachomatis. G = ghost, IB = intermediate body, RB = reticulate body, EB = elementary body; n = 3; bars indicate SE. (TIF) [file ppat.1004883.s012.tif]

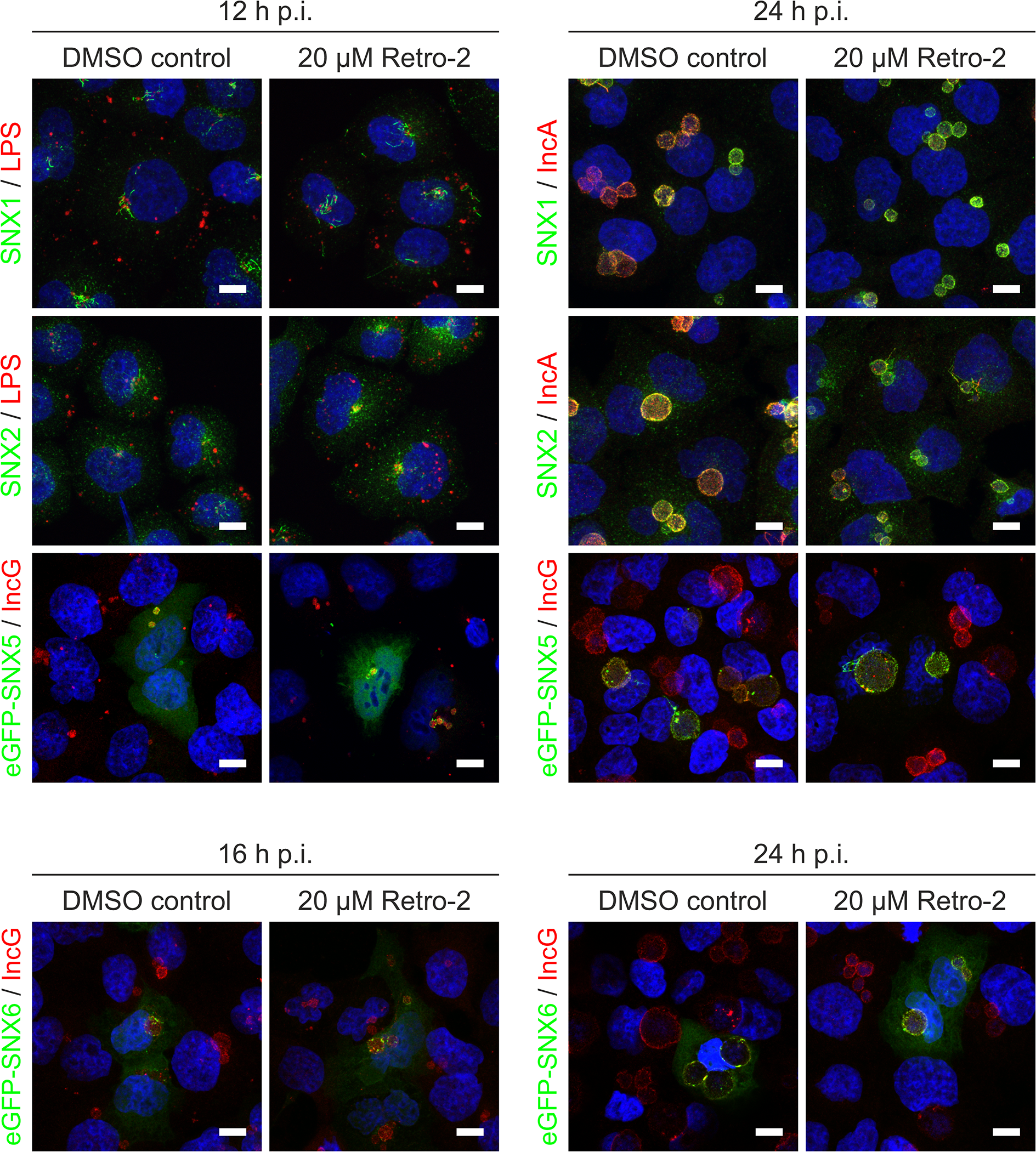

Supplement: S13 Fig — Confocal IF images showing localization of SNX1, SNX2, eGFP-SNX5 and eGFP-SNX6 during C. trachomatis L2 infection (MOI 2) at 12 h (SNX1, SNX2 and eGFP-SNX5), 16 h (eGFP-SNX6) and 24 h p.i. treated with indicated concentrations of Retro-2 or DMSO as solvent control. Cells were stained with indicated antibodies; DNA was stained with DAPI (blue). Images show maximum intensity projections of z-stacks. Scale bar, 10 μm; n = 2. (TIF) [file ppat.1004883.s013.tif]

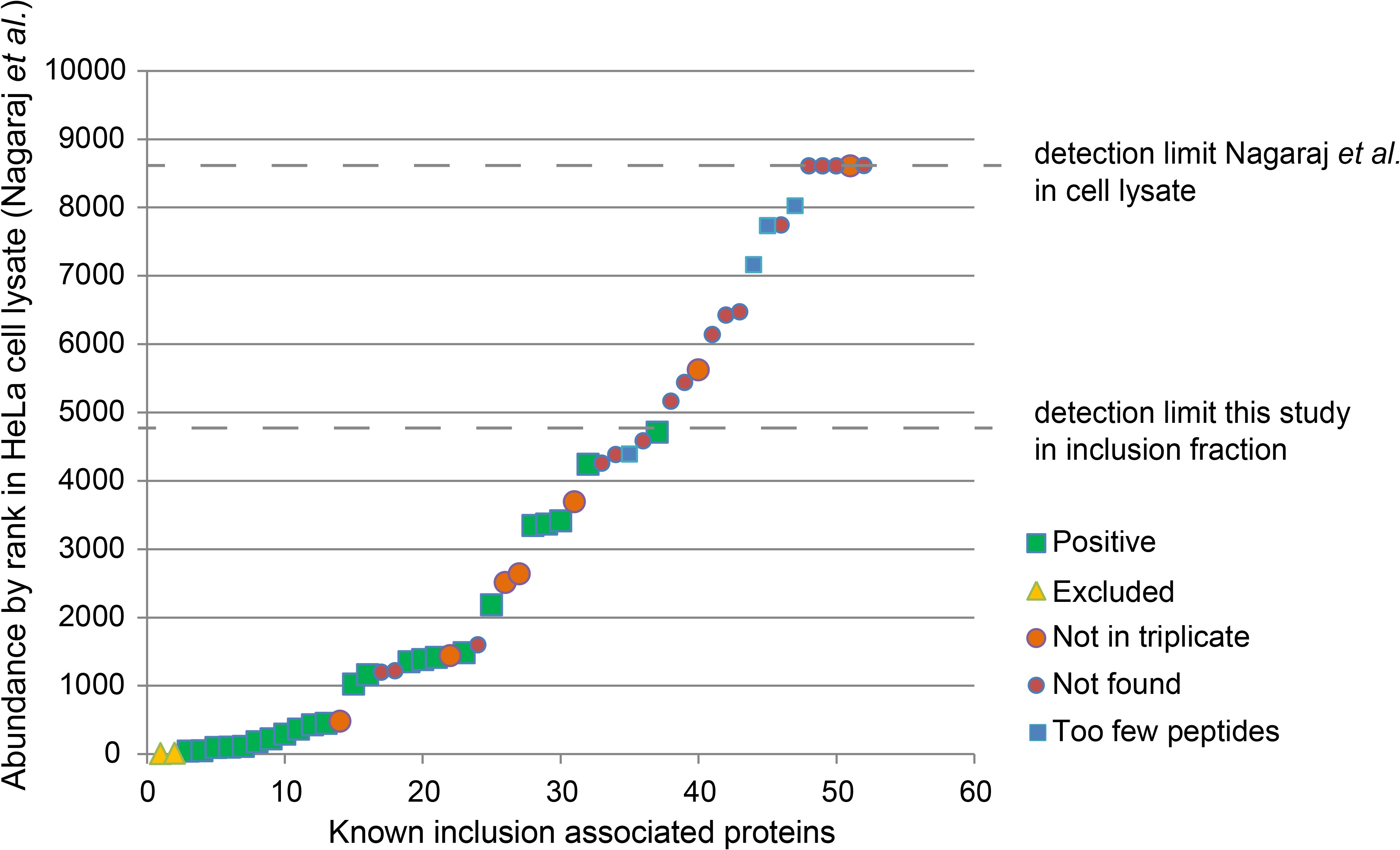

Supplement: S14 Fig — Previously reported inclusion associated proteins were ranked by their abundance in HeLa cell lysates based on iBAQ intensity of tryptic peptides [22]. Proteins that were not found in the lysate are on the detection limit (rank 8604, n = 5). Positive = proteins that passed the SILAC exclusion approach, Excluded = did not pass the SILAC exclusion approach or were removed by initial filtering of common contaminants. Not in triplicate = proteins detected in the inclusion fraction but not in all experiments. Not found = proteins that were never detected in the inclusion fraction. Too few peptides = proteins that were identified in all three experiments but with only one peptide. (TIF) [file ppat.1004883.s014.tif]

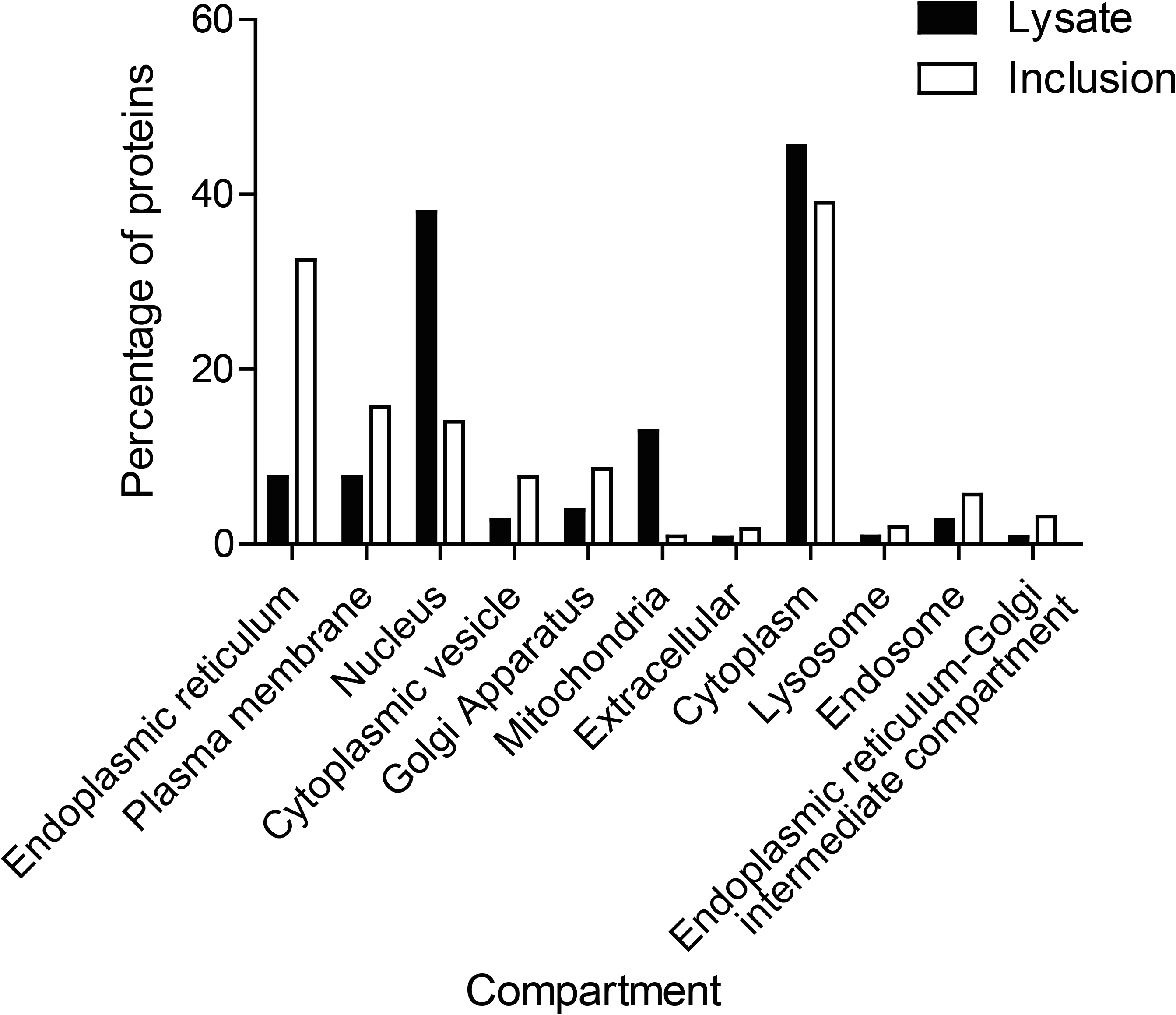

Supplement: S15 Fig — Proteins that were reliably found and quantified in the inclusion and the total cell lysate (Lysate: n = 2002; Inclusion: n = 351) were annotated with subcellular localization data from UniprotKB. The percentage of proteins annotated with the indicated term is shown. One protein can have annotations for several organelles. (TIF) [file ppat.1004883.s015.tif]
