# Supplementary material for: The Proteome of the Isolated Chlamydia trachomatis Containing Vacuole Reveals a Complex Trafficking Platform Enriched for Retromer Components
Source: PLoS Pathog. 2015 Jun 4;11(6):e1004883. doi: 10.1371/journal.ppat.1004883 (PMC4456400; doi:10.1371/journal.ppat.1004883)
Supplement: S1 Text — (DOCX) [file ppat.1004883.s016.docx]

**Supporting Information File:**

**S1 Text: Supplemental Experimental Procedures with references**

**Reagents**

Unless otherwise stated, all reagents were obtained from Sigma Aldrich or Carl Roth. Retro-2 (catalog no. 554715) was purchased from Calbiochem.

**Table: Plasmids**

| **Expressed insert** | **Plasmid name** | **Provider** | **Reference** |
| --- | --- | --- | --- |
| eGFP | peGFP-C1 | Invitrogen |  |
| Rab11A-eGFP | peGFP-Rab11A | Zerial, M. | [1] |
| YFP-Rab3D WT | peYFP-C1-Rab3D | Gerke, V. | [2] |
| YFP-Rab3D T35N | peYFP-C1-Rab3DT35N | Gerke, V. | [2] |
| VCP-eGFP | VCP(wt)-EGFP | Addgene: 23971 | [3] |
| eGFP-SYNGR2 | peGFP- cellugyrin | Kandror, K.V. | [4] |
| YFP-STIM2 | pEX-CMV-SP-YFP-STM2(15-746) | Addgene:18862 | [5] |
| eGFP-DHCR7 | pEYFP-human DHCR7 | Herrmann, H. | [6] |
| eGFP-Rootletin | pEGFP Rootletin | Addgene: 41166 | [7] |
| eGFP-Rac1 | pcDNA3-EGFP-Rac1(wt) | Addgene: 13719 | [8] |
| eGFP-HSPB1 | pEGFP-hsp27 wt FL | Addgene: 17444 | [9] |
| pEGFP-Fis1 | pEGFP-Fis1 | Jendrach, M. | [10] |
| mEmerald-ILK | mEmerald-ILK-C-14 | Addgene: 54126 | - |
| eGFP-Rab35 | GFP-Rab35 WT | Addgene: 47424 | [11] |
| UBXN6-mCherry | pIRESpuro2-UBXD1-mCherry | Addgene: 31835 | - |
| GFP-TFR | pBa.TfR.GFP | Addgene: 45060 | [12] |
| GFP-Syntaxin7 | pMRXIP GFP-Stx7 | Addgene: 45921 | [13] |
| Cofilin-1-eGFP | pEGFP-N1 human cofilin WT | Addgene: 50859 | [14] |
| eYFP-mPIK3R2 | pEYFP-C1-p85beta (mouse) | Addgene: 1408 | - |
| GFP-HSPA8 | pcDNA5/FRT/TO GFP HSPA8 | Addgene: 19487 | [15] |
| Rab8A-eGFP | pEGFP_Rab8a | Addgene: 31803 | [16] |
| HIS-FLAG-HA-YTH | pFRT/TO/HIS/FLAG/HA-YTHDF2 | Addgene: 38089 | [17] |
| mFLII-HA | pcDNA-Flightless-1-LRR-HA (mouse) | Addgene: 21151 | [18] |
| FLAG-PKM2 | pWZL Neo Myr Flag PKM2 | Addgene: 20585 | [19] |
| Syntaxin4-MYC | Syntaxin4-myc-myc-His | Addgene: 12377 | [20] |
| FLAG-mArginase1 | pcDNA3.1-mArg1-Flag | Addgene: 34574 | - |
| eGFP-VPS35 | pEGFP-C1-VPS35 | Seaman, M. N. | [21] |
| eGFP-SNX3 | pEGFP-C1-SNX3 | Seaman, M.N | [22] |
| eGFP-SNX2 | pEGFP-C1-SNX2 | see molecular cloning | |
| eGFP-SNX5 | pEGFP-C1-SNX5 | see molecular cloning | |
| eGFP-SNX6 | pEGFP-C1-SNX6 | see molecular cloning | |
| eGFP-SNX12 | pEGFP-C1-SNX12 | see molecular cloning | |

**Chlamydial infections and stock solutions**

*Chlamydia* stock solutions were prepared as described previously [23]. All standard infections were done in DMEM (Gibco, 4.5 g/l glucose) supplemented with 1 mM sodium pyruvate containing 5 % FCS (Biochrom) at 35°C, using half the culture volume. Infected cells were washed and incubated with fresh infection medium (standard culture volume) at two hours p.i.

**Reinfection assay**

Numbers of infectious EBs were determined as described before [24]. Treated or mock treated cells were infected with the indicated MOI, the cells lysed after the indicated time point using glass beads and 3 minutes of vortexing in 15 ml plastic tubes. Newly formed IFU were titrated as follows: HeLa cells were seeded into 24 well cell culture plates which were prepared with glass cover slips and grown to 80 % confluency. Cells were infected with serial dilutions of lysates for 24 h, before fixation with 2 % paraformalydehyde (PFA) in PBS for 30 min at RT. Fixed cells were immunostained for bacterial heat shock protein 60 (Hsp60). Inclusions in 10 fields of view (FOV) were counted in the appropriate dilution (10-50 per FOV) with a Zeiss Axiovert 40 inverted microscope using the ID Plan Neofluar 40x/0.6 objective. Inclusions per FOV were transformed into inclusion forming units (IFU) per ml. In time course analyses, samples of different time points were frozen at -80°C before processing for titration in batch. This significantly reduces viable progeny and therefore precludes comparison to directly titered experiments.

**Area and number of inclusions**

Treated or mock treated cells were infected with MOI 0.5. Cells were fixed with 2 % PFA in PBS after the indicated amount of time. Immunostaining was performed against bacterial Hsp60 and epifluorescence images were randomly taken at an AxioVert 40 inverted microscope (Zeiss). A script in ImageJ software was used to count the numbers and measure the area of inclusions. The scripts were slightly different for 24 h and 48 h p.i.

*Script for 24 h inclusions:*

run("Enhance Contrast", "saturated=0.01"); //contrast of the image adjusted to 1% of pixels saturated

run("Gaussian Blur...", "sigma=3"); //Gaussian blur to blur noise

run("8-bit");

run("Subtract Background...", "rolling=50"); //removal of noise using rolling circle

run("Auto Local Threshold...", "method=Bernsen radius=20 parameter_1=0 parameter_2=0 white"); //Local auto threshold to binarily define inclusion area

run("Options...", "iterations=1 count=1 black edm=Overwrite do=Nothing"); //set options for “Fill Holes” function

run("Fill Holes"); //Fill holes

run("Watershed"); //separate partly joined particles

run("Analyze Particles...", "size=50-Infinity pixel circularity=0.5-1.00 show=Outlines display clear summarize in_situ"); //count particles and their area

Script for 48 h inclusions

run("Enhance Contrast", "saturated=0.1"); //contrast of the image adjusted to 1% of pixels saturated

run("Subtract Background...", "rolling=1 create sliding"); //used “Subtract Background” function to create a blurred image instead of Gaussian blur

run("8-bit");

run("Auto Local Threshold...", "method=Bernsen radius=30 parameter_1=0 parameter_2=0 white"); //Local auto threshold to binarily define inclusion area

run("Options...", "iterations=1 count=1 black edm=Overwrite do=Nothing"); //set options for “Fill Holes” function

run("Fill Holes"); //Fill holes

run("Watershed"); //separate partly joined particles

run("Analyze Particles...", "size=400-Infinity pixel circularity=0.6-1.00 show=Outlines display clear summarize in_situ"); //count particles and their area

**Recovery assay**

HeLa cells were infected and treated with the indicated compound from 8 h p.i (Retro-2) or 24 h (Penicillin G sodium salt) until 48 h p.i. Samples for each treatment were taken and stored at -80°C for IFU determination. The cells were then washed three times with fresh infection medium and incubated until 96 h p.i.to allow for recovery of the bacteria before harvest for IFU determination. All samples of a replicate were processed in batch for IFU determination as described.

**Quantitative PCR**

For determination of the relative bacterial genome copy number of Retro-2 and DMSO treated cells by quantitative PCR (qPCR), primers to amplify a 78 bp region of the chromosomal GroEL2 gene were used, similar to what was published earlier [25], but with slightly different primer sequences (forward primer: 5’ CATGCTGTGTGGGATTTTGG 3’, reverse primer: 5’ CTGGCTATATTCTACCGTTCCTGTTT 3’). Identical cell numbers were used for all samples and DNA was extracted using the DNeasy Blood and Tissue kit (Qiagen) according to manufacturer’s instructions.

To quantify the relative bacterial genome copy number in siRNA experiments, primers to amplify a region of the 16S rRNA locus were used as published previously [26] (forward primer : 5’ CCGCCAACACTGGGACT 3’, reverse primer: 5’ GGAGTTAGCCGGTGCTTCTTTAC 3’). The cells were lysed by vortexing 3 min with glass beads and DNA was extracted by boiling at 95°C for 20 minutes, followed by 10 minutes centrifugation at 500 *x g,* 4° C to remove cellular debris.

QPCR was performed on a MX3000 thermocycler (Stratagene) using the QuantiTect SYBR Green (Qiagen) kit according to manufacturer’s instructions. Relative genome copy numbers were calculated by assuming [fold difference =2^ΔCt^].

**siRNA (small interfering RNA) mediated knockdowns**

For specific knockdown of target host cell proteins, HeLa cells were transfected with target specific siRNAs as published [27]. Briefly, cells were grown in 12 well plates to around 80 % confluency and transfected with a total of 80 pmol siRNA (approximately 1 µg). The siRNA was dissolved in 100 µl Qiagen RNAi transfection buffer followed by addition of 6 µl RNAi Fect transfection reagent (Qiagen) and incubation for 20 minutes at room temperature (RT) to allow formation of Liposomes. Cells were washed once and incubated with 600 µl fresh media before dropwise addition of the siRNA transfection mixture. Cells were grown for 72 h before starting experiments and successful knockdown was assessed by western blot. The following pooled siRNAs were used:

**Table: siRNAs**

| **Target** | **Sequence (5’ to 3’)** | **Manufacturer** | **Reference/ Catalog Nr.** |
| --- | --- | --- | --- |
| All Stars | Not publicly available | Qiagen | SI03650318 |
| Luciferase | AACUUACGCUGAGUACUUCGA | Qiagen | [27] |
| SNX1 | CGCGGTGGTCAGTAAACATCA | Qiagen | Hs_SNX1_1, SI00047775 |
| SNX1 | CTCGGGTGACTCAATATGAAA | Qiagen | Hs_SNX1_2, SI00047782 |
| SNX2 | TGCTCCTAGAATTGAATCAAA | Qiagen | Hs_SNX2_6, SI04190907 |
| SNX2 | TAGGTAATTCTGAGGATCATA | Qiagen | Hs_SNX2_7, SI04206475 |
| SNX2 | CTGCCTAGAGCAGTTAATACA | Qiagen | Hs_SNX2_8, SI04258394 |
| SNX5 | ACAGGTATATATGGAAACAAA | Qiagen | Hs_SNX5_1, SI00729015 |
| SNX5 | CCCGACTTTGATGGTCCTCGA | Qiagen | Hs_SNX5_6, SI03184342 |
| SNX5 | CCCGTAGTTCGTCTTTAGTTA | Qiagen | Hs_SNX5_7, SI03186190 |
| SNX6 | GCCACTCTTATTTACCTTTAA | Qiagen | Hs_SNX6_7, SI02644698 |
| SNX6 | CCGAAACTTCCCAACAATTAT | Qiagen | Hs_SNX6_8, SI02644705 |

**Molecular cloning**

Standard molecular cloning techniques were performed. For preparation of cDNA, the QuantiTect Reverse Transcription kit (Qiagen) was used according to manufacturer’s instructions. Transcripts were amplified from cDNA by PCR using Phusion Polymerase (NEB) and specific oligonucleotide primers that contained restriction enzyme cleavage sites. Where necessary, DNA was directly cleaned up or gel purified or using the Wizard SV kit (Promega) according to manufacturer’s instructions. DNA restriction digests were performed according to manufacturer’s instructions with standard digest duration of 3 h at 37 °C for both vector and insert using enzymes from NEB. Ligations using T4 DNA ligase (NEB) were performed for 3 h at 16°C with an equimolar vector to insert ratio and a total amount of 50 ng DNA in 10 µl reaction volume, followed by clean up using the Wizard SV kit (Promega). Plasmid DNA was extracted from transformed *E. coli* using Plasmid Mini kit or Plasmid Midi kit (Qiagen) according to manufacturer’s instructions. All final constructs were verified by Sanger sequencing using Big Dye (Applied Biosciences) according to manufacturer’s instructions by the in house sequencing facility of the Robert Koch Institute (RKI), Berlin. The following primer pairs were used for cloning of the indicated inserts into the peGFP-C1 backbone (Invitrogen):

**Table: Oligonucleotide primers for cloning**

| **Expressed insert** | **Restriction site** | **Primer sequence (5' to 3')** | **Direction** |
| --- | --- | --- | --- |
| eGFP-SNX2 | EcoRI | gaatGAATTCAATGGCGGCCGAGAGGGAAC | Forward |
|  | SalI | gttaGTCGACCTAGGCAATGGCTTTGGCTTC | Reverse |
| eGFP-SNX5 | EcoRI | cttaGAATTCAATGGCCGCGGTTCCCGAG | Forward |
|  | SalI | caatGTCGACTCAGTTATTCTTGAACAAGTCAATACAGC | Reverse |
| eGFP-SNX6 | XhoI | gataCTCGAGGCGCCTGCGCCGGCCCTCGCCTC | Forward |
|  | BamHI | ctatGGATCCATTTAACACTGCCAGGCAGTTC | Reverse |
| eGFP-SNX12 | HindIII | atctaaagcttcgTCGGACACGGCAGTAGCTGATAC | Forward |
|  | Kpn | tatggtaccCTACTGGCGCACCTTCCCCGG | Reverse |

**SDS-PAGE**

Standard discontinuous sodium dodecyl sulfate polyacrylamide gel electrophoresis (SDS-PAGE) was performed according to Lämmli et al. For samples containing Percoll, protein extract was prepared by addition of 6 x SDS-PAGE loading buffer, heating to 95 °C for 5 minutes, followed by pelleting insoluble material and Percoll at 20’000 x *g* for 10 minutes. Whole cell extracts were prepared by washing cells twice in well with cold PBS and direct addition of 100 µl 1 x SDS-PAGE loading buffer per 10^6^ cells. Protein extract was heated to 95°C for 5 minutes and insoluble material pelleted by centrifugation at 20’000 x *g* for 10 minutes. Equal cell numbers corresponding to 15-20 µg protein were used for western blot.

**Western blot and immunodetection**

Proteins separated by SDS-PAGE were transferred to a Immobilon-P polyvinylidene difluoride (PVDF) membrane (Millipore) either by semi dry blot in a Trans-Blot SD semi-dry transfer cell (BioRad) or by wet blot in a Mini-Protean Tetra electrophoresis cell (BioRad) according to manufacturer’s instructions. After transfer, the PVDF membrane was incubated in Tris buffered saline supplemented with tween and 3 % milk (TBST-M) for 1 h at RT, followed by incubation with the primary antibody over night at 4 °C or for 3 h at RT on a rotating mixer. The membrane was washed 3 x 10 min with TBS-T followed by incubation with a species specific horse radish peroxidase linked secondary antibody (Amersham Biosciences) for 1 h at RT. The membrane was washed again (3 x 10 min) with TBS-T before the antibody was detected by enhanced chemiluminescence (ECL) using the ECL Plus kit (Pierce) to expose an X-ray film (Kodak). The film was developed in a Citrix 60 developer (Agfa) and scanned on a Scanjet G4050 flatbed scanner (Hewlett Packard) for digitalization.

Polyclonal mouse or rabbit anti-IncG antibody was produced by immunization of mice or rabbits, respectively. For immunization, the C-terminal cytoplasmic fragment of IncG (V91-F167) fused to *Schistosoma japonicum* glutathione *S*-transferase (GST) was used. All animal handling was performed by Biogenes, Berlin. The antigen was produced in *Escherichia coli* BL21 (Merck) using the pGEX-3X N-terminal GST expression vector (GE Healthcare). Cloning was done by using EcoRI and BamHI restriction sites in *E.coli* DH5α (Invitrogen). The following primers were used: forward: 5’-CGCGGGATCCTAGTTGTAAAAAGAGATCACCTGAAG-3’; reverse: 5’- CCGGGAATTCATGTCATCCTTAGAAGGAG-3’. GST-IncG fusion protein was expressed and purified using Glutathione HiCap Matrix slurry (Qiagen) according to manufacturer’s instructions. IncG-specific antibodies were directly used from animal sera.

| **Table: Primary antibodies** | |  |  |
| --- | --- | --- | --- |
| **Antigen** | **From species** | **Source** | **Catalog Nr.** |
| SNX1 | mouse | BD Bioscience | 611482 |
| SNX2 | mouse | BD Bioscience | 611308 |
| SNX5 | rabbit | Abcam | ab180520 |
| SNX6 | goat | Santa Cruz | sc-8679 |
| VPS35 | goat | Imgenex | IMG-3575 |
| VPS35 | mouse | Abcam | ab57632 |
| CI-M6PR | mouse | AbD seroTec | MCA2048 |
| IncA | rabbit | In house | [28] |
| IncG | rabbit | In house |  |
| IncG | mouse | In house |  |
| GAPDH | mouse | Pierce | MA5-15738 |
| Sam68 | mouse | Santa Cruz | sc-1238 |
| Hsp60 | mouse | Enzo Life Sciences | ALX-804-072 |
| Hsp60 | mouse | Enzo Life Sciences | ALX-804-071 |
| COXI | mouse | Santa Cruz | sc-58347 |
| GRP94 | mouse | GeneTex | 40016 |
| p62 | mouse | BD Biosciences | 610497 |
| LAMP-1 | mouse | BD Biosciences | 611042 |
| Bcl-2 | mouse | BD Biosciences | 610538 |
| Annexin II | mouse | BD Biosciences | 610068 |
| ß-actin | mouse | Sigma | A5441 |
| MOMP | mouse | [29] |  |
| STIM1 | mouse | BD Biosciences | 610954 |
| Sec22b | rabbit | Synaptic Systems | 186 003 |
| SQSTM1 | mouse | abnova | H00008878-M01 |
| HA-probe (Y-11) | mouse | Santa Cruz | sc-805 |
| ANTI-FLAG M2 | mouse | Sigma | F3165 |
| Anti-myc | mouse | Santa Cruz | sc-40 |

| **Table: Secondary antibodies** |  |  |
| --- | --- | --- |
| **Name** | **Use** | **Manufacturer** |
| ECL anti-mouse IgG, HRP conjugated | WB | Amersham Biosciences |
| ECL anti-rabbit IgG, HRP conjugated | WB | Amersham Biosciences |
| Cy2: Goat anti-mouse IgG | IF | Dianova |
| Cy2: Goat anti-rabbit IgG | IF | Dianova |
| Cy3: Goat anti-mouse IgG | IF | Dianova |
| Cy3: Goat anti-rabbit IgG | IF | Dianova |
| Cy5: Goat anti-mouse IgG | IF | Dianova |
| Alexa Fluor 488: goat anti mouse IgG | IF | Dianova |
| Alexa Fluor 647: goat anti rabbit IgG | IF | Dianova |
| MACS goat anti-rabbit IgG micro-beads | MACS | Miltenyi |

**Immunostaining**

Indirect immunofluorescence staining was performed as described [30]. For live cells microscopy, cells were seeded onto glass-bottom live cell dishes (ibidi µ dishes) and treated as indicated before imaging. Immunostained samples and live cell samples were analyzed on a Zeiss LSM 780 LSCM equipped with Zeiss Zen software. Images were processed with Adobe Photoshop CS6 for image corrections (brightness, contrast, intensity) where necessary.

**Inclusion counting**

Inclusions in HSMG lysis buffer were counted by hand on a Zeiss Axiovert 40 phase contrast microscope using KOVA Glasstic 10 slides (Hycor). Visually intact inclusions were identified by eye by a characteristic halo surrounding the spheres in solution. No size exclusion was performed, counted inclusion diameters ranged from approximately 3 to 15 µm. At least five fields were counted for each sample with a minimum of 50 counted inclusions in total.

**Small scale isolations for validations by confocal microscopy**

Two wells of a six well plate (3 x 10^6^ cells) were each transfected with 1 µg of the respective plasmid using Lipofectamine2000 as described above. 4 hours after transfection, cells were infected with MOI = 2 for 24 h. After removal of the media, cells were washed with PBS, followed by a wash with HSMG buffer. The cells were then scraped into 2 ml lysis buffer containing 33 % Percoll and lysed by 13 passages in a ball homogenizer (Isobiotec) using 16 µm clearance. The lysate was split into two 2 ml tubes and each taken to 1.6 ml. The lysate was then centrifuged at 19´000 x *g* for 55 minutes at 4°C in a R5417R centrifuge (Eppendorf). The top 1 ml was removed and the lower fractions pooled, diluted 1:2 with HSMG and spun down at 1500 x *g* for 10 minutes on a Poly-D-Lysine coated live cell dish (ibidi) for microscopic analysis.

**Immunofluorescence of isolated inclusions**

Inclusions were centrifuged (1200 x *g* for 10 minutes) on Poly-D-Lysine coated live cell dishes. The specimen was fixed with 4 % PFA in HSM (HSMG without EGTA) buffer and the dish incubated over night at 4°C. The buffer was exchanged with PBS and IF was performed in well essentially as described for whole cells.

**Transmission electron microscopy (TEM)**

For TEM of isolated inclusions, freshly isolated inclusions were centrifuged (1200 *x g*, 10 minutes) on Poly-D-Lysine coated live cell dishes (ibidi µ-dish). The specimen was fixed with 4 % PFA in HSM buffer and the dish incubated at RT for 1h. The buffer was exchanged with glutaraldehyde fixation solution (2.5 % glutaraldehyde, 50 mM HEPES) and incubated at 4°C overnight. The samples were post-fixed in 1 % osmium tetroxide followed by dehydration through a series of increasing ethanol concentrations. The samples were infiltrated with acetone before embedding in epon. Embedded samples were polymerized for 48 h at 60 °C.

For morphology assays, infected cells were treated as indicated (see Fig 6E) in six well plates and fixed with glutaraldehyde (2.5 % in 50 mM HEPES) for 2 h at room temperature, harvested by scraping and embedded in low-melting point agarose. Samples were post fixed with osmium tetroxide (1% in distilled water) followed by tannic acid (0.1 % in 50 mM HEPES) and block contrasting with uranyl acetate (2% in distilled water). Dehydration was done by incubation in a stepwise-graded ethanol series. Finally, samples were embedded in epon resin which was polymerized for 48 h at 60 °C. Pictures were taken from each sample at randomly selected positions as described below. The morphology of the bacteria was assessed by manual counting of at least 500 bacteria per experimental treatment. Ultra-thin sectioning of epon-embedded samples was performed using a Leica UC7 ultramicrotome and sections were stained with uranyl acetate and lead citrate to increase contrast. The sections were examined using a FEI Tecnai12 transmission electron microscope operated at 120 kV. Digital images were taken with an OSIS Megaview III CCD camera.

**SILAC labeling of HeLa cells**

HeLa cells were labeled by culturing for 6 passages in SILAC DMEM (Dulbecco’s modiﬁed Eagle's medium, PAA) containing either isotope labeled (^13^C_6_^15^N_4_ L- arginine, ^13^C_6_^15^N_2_ L-Lysine ) or unlabeled (^12^C_6_^14^N_4_ L-arginine, ^12^C_6_^14^N_2_ L-Lysine) L-lysine (96 mg/l) and L-arginine (42 mg/l) and 10 % dialyzed FCS (dFCS, Biochrom). To reduce metabolic proline to arginine conversion due to oversupply of arginine in the growth medium [31], the concentration of L-arginine and L-lysine was titrated to conditions where the growth rate of cells was equal to standard DMEM concentrations over 10 passages. Complete incorporation of isotope labeled amino acids was assessed by LC-MS/MS of tryptic peptides. Incorporation of heavy labeled amino acids to peptides was above 98 % (average) with 88 % of peptides appearing only in the heavy labeled state. Complete labeling was therefore assumed for all bioinformatic purposes.

**SILAC MACS inclusion isolation**

SILAC labeled cells were thawed and passaged for a maximum of 4 passages and cultured in the appropriate media containing 10 % dFCS with growth conditions and handling equal to normal cell culture. For infections, cells were washed with SILAC DMEM, and infected with *C. trachomatis* L2 at an MOI of 4 for 24 h at 35 °C in SILAC DMEM supplemented with 5 % non-dialyzed FCS (infection, H label) or 5 % dialyzed FCS (mock controls, L label) and the appropriate amino acids using half the culture volume of media. The inoculum was replaced with fresh infection media (standard volume) after washing once with SILAC DMEM at 2 h p.i.

Inclusions were isolated as described above but 6 x 10^7^ infected cells were mixed with the same amount of mock infected cells before lysis. The protocol was carried out in twice the volume (32 ml total gradient volume) and 2 x 6 ml crude inclusions were loaded on one column before washing with 3 x 12 ml HSMG.

Isolated inclusions were concentrated to 30 µl by spinning down at 1200 x g for 10 minutes. Inclusion samples were prepared for LC-MS/MS by FASP. 10 % of the sample was used for direct injection after desalting by STAGE tip. The remaining peptides were separated by strong anion exchange chromatography into 6 fractions before desalting by STAGE tip followed by LC-MS/MS as described below.

**Sample preparation by FASP and peptide pre-fractionation**

For sample preparation for LC-MS/MS of both whole cell lysates and isolated inclusions the FASP procedure was used essentially as previously described [32]. For tryptic digests, sequencing grade modified trypsin (Promega) was used. For desalting of tryptic peptides Stop and Go extraction (STAGE) were used as previously described [33,34], using Empore C18 disks (3M).

Where indicated, tryptic peptides were fractionated into six fractions using strong anion exchange (SAX) chromatograhy in STAGE tip format as previously described [32,35]. In brief, SAX tips were prepared by stacking six layers of Empore Anion Exchange disks (3M) in a 200 µl pipet tip. The peptide solution was diluted with Britton and Robinson universal buffer (BR, 20 mM acetic acid, 20 mM phosphoric acid, 20 mM boric acid) at pH 11 before assembling the conditioned SAX tip into a freshly conditioned STAGE tip. The peptide solution was then loaded by centrifugation at 3000 x *g* for 1 min followed by a wash with 100 µl BR at pH 11. The STAGE tip was replaced and the SAX fractions were eluted subsequently with BR at pH 8, 6, 5, 4 and 3 into individual STAGE tips. STAGE tips were washed with 50 µl 0.1 % TFA before elution with 60 % ACN directly into glass vials or 96 well plates. Fractionated peptides were dried in a SpeedVac (Eppendorf) and resuspended in 20 µl Buffer A (0.1 % acetic acid, 2 % ACN).

**LC-MS/MS**

Tryptic peptides were analyzed on a Q Exactive mass spectrometer (Thermo) coupled to a TriVersa NanoMate source (Advion). Ten µl of tryptic peptides were separated with an UltiMate 3000 nHPLC (Dionex). Peptides were ionized by ESI. Approximately 1 µg of peptides were loaded on an Acclaim PepMap 100 precolum (Thermo) (0.75 µm inner diameter, packed with 3 µm C_18_ particles). Separation by reversed phase chromatography was achieved on a 25 cm Acclaim PepMap RSLC C_18_ column (Thermo) with 2 µm C_18_ particles using a 120 min linear gradient from 2 % to 25 % Buffer A (0.1 % acetic acid, 2 % ACN) in Buffer B (0.1 % acetic acid in ACN) at a flow rate of 300 nl/ min. The column was heated to 40 °C. The mass spectra were acquired in a data-dependent “top 10” method which dynamically chooses the ten most abundant precursor ions from the survey scan (300-1650 m/z, 70’000 resolution, 120 ms injection time, automatic gain control target value of 3 x 10^6^). Precursors with a charge of ≥2 were isolated within a 3 m/z window and fragmented by higher energy collisional dissociation using normalized collision energy of 27.5 %, automatic gain control target value of 2 x 10^5^ and 17’500 resolution, 100 m/z fixed first mass. Dynamic exclusion was defined by exclusion duration of 30 s.

**Analysis of raw data and initial filtering**

Raw data was analyzed with MaxQuant Version 1.3.0.5 [36] in standard settings with the requantify feature enabled using a false discovery rate (FDR) of < 1%. Quantification by intensity based absolute quantification (iBAQ) was enabled [37], without introduction of external reference peptides (iBAQ logarithmic fit disabled). The human reviewed reference proteome .FASTA file (organism 9606, keyword 1185) and the reference proteome for *C. trachomatis* serovar L2 (strain 434/Bu / ATCC VR-902B) were retrieved from uniprot.org on September 12, 2012 and concatenated. The .raw files for the lysates (3 .raw files) were processed independently of the .raw files of the fractionated and full inclusion measurements. All six SAX fractions plus the overview fraction of each experiment were treated as a single experiment and analyzed together (21 .raw files).

The data obtained from MaxQuant was filtered initially by removing common contaminations included in the common contaminations FASTA file provided by MaxQuant 1.3.0.5 [36], as well as identifications based solely on proteins from the decoy database (reverse database from .FASTA file). Furthermore only proteins that were found in all three experiments of a triplicate were retained.

For determination of the host cell derived proteome of the inclusion, all bacterial proteins were filtered. Protein groups that had less than two unique+ razor peptides in at least one experiment were filtered.

**Statistical test for enrichment (SILAC exclusion approach)**

Each host protein in the inclusion fraction data set that showed three (first test, n = 1095) or two (second test, n = 305) SILAC ratios was tested for a significant shift of its ratios compared to the empirical lysate distribution.

SILAC ratios of all the proteins after filtering were transformed by taking the logarithm. An empirical distribution was calculated based on the lysate SILAC ratios of proteins which were measured in both data sets (lysate and inclusion fractions, each in three replicates). A two-sided Wilcoxon test was applied to determine the differentially enriched proteins in the inclusion fraction data. P-values were adjusted for multiple testing by the Benjamini-Hochberg approach. If proteins were quantified by three SILAC ratios, an adjusted p-value of 0.01 was required, whereas a less stringent adjusted p-value of 0.05 was required for those proteins which only showed a SILAC ratio in two out of three experiments, as the Wilcoxon test statistic is more limited in its number of attainable p-values for this scenario.

For the first test with three SILAC ratios, the overlap of proteins between the data sets corresponded to 724 proteins of 1095 proteins and overall 1882 lysate ratios contributed to the distribution since not all lysate proteins showed a SILAC ratio in all of the 3 replicates. As a result, 892 human proteins were reported to be differentially enriched, among these, 253 were enriched in the inclusion fraction of infected cells with a p-value of below 0.01.

In the second test, the overlap between proteins with either two or three SILAC ratios in the inclusion fraction was used for the empirical lysate distribution, resulting in a total of 903 proteins with 2344 contributing SILAC ratios from the lysate fraction. As a result, an additional 191 proteins were differentially enriched, among these, 98 proteins were enriched in the inclusion fraction with p-values below 0.05.

**Abundance analyses**

For the lysate proteome, the relative abundance of each protein was determined by performing a sumtotal normalization. In short, the iBAQ intensity of a protein was divided by the summed iBAQ intensites of all proteins that were found in all three experiments after initial filtering and had a reported iBAQ intensity (n = 2002).

$$[{{iBAQ}_{N} (x)]}_{Lys}=\frac{[{iBAQ (x)]}_{Lys}}{\sum_{i=1}^{n} [{iBAQ (i)]}_{Lys}} \left( 1 \right)$$

with n = total number of protein groups in the lysate

For the abundance of proteins in the inclusion proteome, only proteins that passed the SILAC based cutoff were considered. We estimate an initial protein abundance factor (ipaf)

$ipaf=\frac{1}{n}\sum_{i=1}^{n} [{SILAC L/H (i)]}_{Lys} (2$)

as the ratio of the average SILAC ratio of the respective experiment in the lysate (after filtering as described above) . We then normalize the L iBAQ intensity by the ipaf to account for difference in the initial protein abundance

$$[{{iBAQ_{L}}_{N*} (x)]}_{Inc}= \frac{[{iBAQ\_L (x)]}_{Inc}}{ipaf} (3)$$

We then further normalize each iBAQ L value to account for the proportion of contaminating protein. Here, the SILAC ratio of the protein in the inclusion fractions serves as an estimate of the proportion of contaminating protein.

$$[{{iBAQ_{L}}_{N} (x)]}_{Inc}= [{{iBAQ_{L}}_{N*} (x)]}_{Inc}\left( 1-\frac{1}{[{SILAC L/H (x)]}_{Inc}} \right) (4)$$

After this, a sumtotal normalization was performed to determine the relative contribution of each protein to the inclusion proteome. To this end the normalized iBAQ intensity of a protein was divided by the summed normalized iBAQ intensity of all protein groups (m = 351).

$$[{{iBAQ}_{N} (x)]}_{Inc}=\frac{[{{iBAQ_{L}}_{N} (x)]}_{Inc}}{\begin{aligned} \sum_{i=1}^{m} [{iBAQ\_L_{N} (i)]}_{Inc} \\ \end{aligned}} \left( 5 \right)$$

with m = total number of proteins in the inclusion

For proteins that had a coefficient of variation above 0.5, the protein group was flagged as only limited quantitative (621 of 2002 in lysates and 145 of 351 in inclusion fractions). For the gene ontology (GO) distribution and enrichment analyses also non-quantitative protein groups were considered as their total percentage was negligible (10 % of total iBAQ intensity).

**iBAQ enrichment score**

The iBAQ enrichment score equals the ratio of the relative contributions of inclusion vs. lysate as described above.

$$iBAQ enrichment score \left( x \right)= \frac{[{{iBAQ}_{N} (x)]}_{Inc}}{[{{iBAQ}_{N} (x)]}_{Lys}} (6)$$

For proteins that were never found in in the lysates, we used a published dataset in which HeLa cells were prepared with the same protocols we used and analyzed with a similar proteomics platform but in more depth, to approximate the relative abundance of these proteins in the samples [23]. Only proteins that were identified based on tryptic peptides were used and these proteins were also flagged. In total, 228 protein groups of the inclusion dataset were found in the lysate in all three experiments with at least 2 unique + razor peptides per experiment. An additional 33 with less than 2 unique + razor peptides per experiment and 44 in less than three experiments. 40 were not quantified nor found in the lysate, all of which were reliably quantified in the inclusion dataset and the published dataset. Three protein groups were neither found in the published dataset (SH3TC1, SH3TC2; PRAMEF4, 5, 6, 9, 20, 23 and DNAH6). In all cases protein groups were matched by their Majority protein identifiers (IDs) among different datasets and, if necessary, matched by the first identifier (10 proteins) or by hand (2 proteins) due to different .FASTA files used for processing. The obtained enrichment scores were capped at an upper limit of 250 fold.

**Organellar contribution analysis and enrichment analysis**

The organellar contribution of proteins from both datasets (inclusion and lysate) was calculated individually. Subcellular localization data was retrieved from UniprotKB [38] for all proteins. The first protein ID was used in the case of protein groups consisting of multiple IDs.

GO enrichment analyses were performed with GOrilla [39], GO data [40] was current as of October 11, 2014. The dataset of the HeLa proteome from Nagaraj *et al.* [41] was used of as background after filtering for 2 or more unique tryptic peptides (n = 6331) of which 5635 proteins were mappable to GO terms, to account for detection bias in mass spectrometric measurements.

**Protein protein interaction analysis**

Protein-protein interactions of inclusion associated proteins annotated with the highly enriched GO term “protein localization” (GO:0008104, n = 86) were analyzed using STRING 9.1 [42] with standard settings in confidence view as of May 25, 2014. The interaction network was imported to Cytoscape 3.1.0 [43] and annotated with quantitative experimental data.

**Statistical analysis**

Data apart from proteome analyses are indicated as mean ± standard deviation (SD) or standard error of the mean (SE). Significant differences between means were determined using Student’s *t* test. P-values below 0.05 were considered to be significant.

**Supplemental Experimental Procedures References**

1. Sonnichsen B, De Renzis S, Nielsen E, Rietdorf J, Zerial M (2000) Distinct membrane domains on endosomes in the recycling pathway visualized by multicolor imaging of Rab4, Rab5, and Rab11. J Cell Biol 149: 901-914.

2. Knop M, Aareskjold E, Bode G, Gerke V (2004) Rab3D and annexin A2 play a role in regulated secretion of vWF, but not tPA, from endothelial cells. EMBO J 23: 2982-2992.

3. Tresse E, Salomons FA, Vesa J, Bott LC, Kimonis V, et al. (2010) VCP/p97 is essential for maturation of ubiquitin-containing autophagosomes and this function is impaired by mutations that cause IBMPFD. Autophagy 6: 217-227.

4. Li LV, Bakirtzi K, Watson RT, Pessin JE, Kandror KV (2009) The C-terminus of GLUT4 targets the transporter to the perinuclear compartment but not to the insulin-responsive vesicles. Biochem J 419: 105-112, 101 p following 112.

5. Brandman O, Liou J, Park WS, Meyer T (2007) STIM2 is a feedback regulator that stabilizes basal cytosolic and endoplasmic reticulum Ca2+ levels. Cell 131: 1327-1339.

6. Zwerger M, Kolb T, Richter K, Karakesisoglou I, Herrmann H (2010) Induction of a massive endoplasmic reticulum and perinuclear space expansion by expression of lamin B receptor mutants and the related sterol reductases TM7SF2 and DHCR7. Mol Biol Cell 21: 354-368.

7. Bahe S, Stierhof YD, Wilkinson CJ, Leiss F, Nigg EA (2005) Rootletin forms centriole-associated filaments and functions in centrosome cohesion. J Cell Biol 171: 27-33.

8. Kraynov VS, Chamberlain C, Bokoch GM, Schwartz MA, Slabaugh S, et al. (2000) Localized Rac activation dynamics visualized in living cells. Science 290: 333-337.

9. Voss OH, Batra S, Kolattukudy SJ, Gonzalez-Mejia ME, Smith JB, et al. (2007) Binding of caspase-3 prodomain to heat shock protein 27 regulates monocyte apoptosis by inhibiting caspase-3 proteolytic activation. J Biol Chem 282: 25088-25099.

10. Mai S, Klinkenberg M, Auburger G, Bereiter-Hahn J, Jendrach M (2010) Decreased expression of Drp1 and Fis1 mediates mitochondrial elongation in senescent cells and enhances resistance to oxidative stress through PINK1. J Cell Sci 123: 917-926.

11. Allaire PD, Seyed Sadr M, Chaineau M, Seyed Sadr E, Konefal S, et al. (2013) Interplay between Rab35 and Arf6 controls cargo recycling to coordinate cell adhesion and migration. J Cell Sci 126: 722-731.

12. Burack MA, Silverman MA, Banker G (2000) The role of selective transport in neuronal protein sorting. Neuron 26: 465-472.

13. Itakura E, Kishi-Itakura C, Mizushima N (2012) The hairpin-type tail-anchored SNARE syntaxin 17 targets to autophagosomes for fusion with endosomes/lysosomes. Cell 151: 1256-1269.

14. Garvalov BK, Flynn KC, Neukirchen D, Meyn L, Teusch N, et al. (2007) Cdc42 regulates cofilin during the establishment of neuronal polarity. J Neurosci 27: 13117-13129.

15. Hageman J, Kampinga HH (2009) Computational analysis of the human HSPH/HSPA/DNAJ family and cloning of a human HSPH/HSPA/DNAJ expression library. Cell Stress Chaperones 14: 1-21.

16. Guizetti J, Schermelleh L, Mantler J, Maar S, Poser I, et al. (2011) Cortical constriction during abscission involves helices of ESCRT-III-dependent filaments. Science 331: 1616-1620.

17. Baltz AG, Munschauer M, Schwanhausser B, Vasile A, Murakawa Y, et al. (2012) The mRNA-bound proteome and its global occupancy profile on protein-coding transcripts. Mol Cell 46: 674-690.

18. Li J, Yin HL, Yuan J (2008) Flightless-I regulates proinflammatory caspases by selectively modulating intracellular localization and caspase activity. J Cell Biol 181: 321-333.

19. Boehm JS, Zhao JJ, Yao J, Kim SY, Firestein R, et al. (2007) Integrative genomic approaches identify IKBKE as a breast cancer oncogene. Cell 129: 1065-1079.

20. Low SH, Vasanji A, Nanduri J, He M, Sharma N, et al. (2006) Syntaxins 3 and 4 are concentrated in separate clusters on the plasma membrane before the establishment of cell polarity. Mol Biol Cell 17: 977-989.

21. Gokool S, Tattersall D, Seaman MN (2007) EHD1 interacts with retromer to stabilize SNX1 tubules and facilitate endosome-to-Golgi retrieval. Traffic 8: 1873-1886.

22. Vardarajan BN, Bruesegem SY, Harbour ME, Inzelberg R, Friedland R, et al. (2012) Identification of Alzheimer disease-associated variants in genes that regulate retromer function. Neurobiol Aging 33: 2231 e2215-2231 e2230.

23. Al-Younes HM, Rudel T, Meyer TF (1999) Characterization and intracellular trafficking pattern of vacuoles containing Chlamydia pneumoniae in human epithelial cells. Cell Microbiol 1: 237-247.

24. Heuer D, Rejman Lipinski A, Machuy N, Karlas A, Wehrens A, et al. (2009) Chlamydia causes fragmentation of the Golgi compartment to ensure reproduction. Nature 457: 731-735.

25. Miyairi I, Mahdi OS, Ouellette SP, Belland RJ, Byrne GI (2006) Different growth rates of Chlamydia trachomatis biovars reflect pathotype. J Infect Dis 194: 350-357.

26. Lienard J, Croxatto A, Aeby S, Jaton K, Posfay-Barbe K, et al. (2011) Development of a new chlamydiales-specific real-time PCR and its application to respiratory clinical samples. J Clin Microbiol 49: 2637-2642.

27. Rejman Lipinski A, Heymann J, Meissner C, Karlas A, Brinkmann V, et al. (2009) Rab6 and Rab11 regulate Chlamydia trachomatis development and golgin-84-dependent Golgi fragmentation. PLoS Pathog 5: e1000615.

28. Banhart S, Saied EM, Martini A, Koch S, Aeberhard L, et al. (2014) Improved plaque assay identifies a novel anti-Chlamydia ceramide derivative with altered intracellular localization. Antimicrob Agents Chemother.

29. Gurumurthy RK, Maurer AP, Machuy N, Hess S, Pleissner KP, et al. (2010) A loss-of-function screen reveals Ras- and Raf-independent MEK-ERK signaling during Chlamydia trachomatis infection. Sci Signal 3: ra21.

30. Heuer D, Brinkmann V, Meyer TF, Szczepek AJ (2003) Expression and translocation of chlamydial protease during acute and persistent infection of the epithelial HEp-2 cells with Chlamydophila (Chlamydia) pneumoniae. Cell Microbiol 5: 315-322.

31. Blagoev B, Mann M (2006) Quantitative proteomics to study mitogen-activated protein kinases. Methods 40: 243-250.

32. Wisniewski JR, Zougman A, Mann M (2009) Combination of FASP and StageTip-based fractionation allows in-depth analysis of the hippocampal membrane proteome. J Proteome Res 8: 5674-5678.

33. Rappsilber J, Ishihama Y, Mann M (2003) Stop and go extraction tips for matrix-assisted laser desorption/ionization, nanoelectrospray, and LC/MS sample pretreatment in proteomics. Anal Chem 75: 663-670.

34. Rappsilber J, Mann M, Ishihama Y (2007) Protocol for micro-purification, enrichment, pre-fractionation and storage of peptides for proteomics using StageTips. Nat Protoc 2: 1896-1906.

35. Ishihama Y, Rappsilber J, Mann M (2006) Modular stop and go extraction tips with stacked disks for parallel and multidimensional Peptide fractionation in proteomics. J Proteome Res 5: 988-994.

36. Cox J, Mann M (2008) MaxQuant enables high peptide identification rates, individualized p.p.b.-range mass accuracies and proteome-wide protein quantification. Nat Biotechnol 26: 1367-1372.

37. Schwanhausser B, Busse D, Li N, Dittmar G, Schuchhardt J, et al. (2011) Global quantification of mammalian gene expression control. Nature 473: 337-342.

38. UniProt C (2014) Activities at the Universal Protein Resource (UniProt). Nucleic Acids Res 42: D191-198.

39. Eden E, Navon R, Steinfeld I, Lipson D, Yakhini Z (2009) GOrilla: a tool for discovery and visualization of enriched GO terms in ranked gene lists. BMC Bioinformatics 10: 48.

40. Ashburner M, Ball CA, Blake JA, Botstein D, Butler H, et al. (2000) Gene ontology: tool for the unification of biology. The Gene Ontology Consortium. Nat Genet 25: 25-29.

41. Nagaraj N, Wisniewski JR, Geiger T, Cox J, Kircher M, et al. (2011) Deep proteome and transcriptome mapping of a human cancer cell line. Mol Syst Biol 7: 548.

42. Franceschini A, Szklarczyk D, Frankild S, Kuhn M, Simonovic M, et al. (2013) STRING v9.1: protein-protein interaction networks, with increased coverage and integration. Nucleic Acids Res 41: D808-815.

43. Shannon P, Markiel A, Ozier O, Baliga NS, Wang JT, et al. (2003) Cytoscape: a software environment for integrated models of biomolecular interaction networks. Genome Res 13: 2498-2504.
