# Supplementary material for: The Proteome of the Isolated Chlamydia trachomatis Containing Vacuole Reveals a Complex Trafficking Platform Enriched for Retromer Components
Source: PLoS Pathog. 2015 Jun 4;11(6):e1004883. doi: 10.1371/journal.ppat.1004883 (PMC4456400; doi:10.1371/journal.ppat.1004883)
Supplement: S2 Table — Trivial names were used as reported in the cited manuscripts. The Uniprot identifier (ID) of the reviewed human protein is shown for each protein except for actin where the exact proteins were not defined. ISO indicates if a highly homologous variant was found. (DOCX) [file ppat.1004883.s018.docx]

S2 Table: Overview of proteins that associate with the inclusion of *C. trachomatis* at 24 h p.i.

| **Trivial Name** | **Uniprot ID** | **Found** | **Reference** |
| --- | --- | --- | --- |
| 14-3-3β | P31946 | YES | [1] |
| ABCA1 | O95477 |  | [2] |
| ACBD6 | Q9BR61 |  | [3] |
| ACSL3 | O95573 | YES | [3] |
| Actin |  |  | [4] |
| ApoA1 | P02647 |  | [2] |
| Arf1 | P84077 | YES | [5] |
| BAD | Q92934 |  | [6] |
| BICD1 | Q96G01 |  | [7] |
| CERT | Q9Y5P4 | YES | [8] |
| Calreticulin | P27797 | YES | [9] |
| CLA1 | Q8WTV0 |  | [2] |
| CD59 | P13987 | YES | [10] |
| CD63 | P08962 |  | [11,12] |
| COG3 | Q96JB2 |  | [13] |
| COG8 | Q96MW5 |  | [13] |
| Cytokeratin 18 | P05783 | YES | [4] |
| Derlin-1 | Q9BUN8 |  | [14] |
| DP-1 (REEP5) | Q00765 | YES | [14] |
| Fyn | P06241 |  | [15] |
| GBP1 | P32455 |  | [8] |
| GS15 | Q9NYM9 |  | [13] |
| IP3-R | Q14643/Q14573 | YES | [9] |
| LPCAT1 | Q8NF37 | YES | [3] |
| MAP1-LC3 | Q9H492 |  | [16] |
| MYPT1 | O14974 | YES | [17] |
| OCRL1 | Q01968 |  | [5] |
| PDI | P07237 | YES | [14] |
| PI4KIIα | Q9BTU6 |  | [5] |
| PKCδ | Q05655 | YES | [18] |
| Rab1A | P62820 | YES (ISO) | [19] |
| Rab11A | P62491 | YES (ISO) | [19] |
| RAB11FIP2 | Q7L804 |  | [20] |
| Rab14 | P61106 | YES | [21] |
| Rab4A | P20338 |  | [19] |
| Rab4B | P61018 |  | [19] |
| Rab6A | P20340 | YES | [19] |
| Rab6B | Q9NRW1 | YES (ISO) | [19] |
| Raf1 | P04049 |  | [22] |
| RTN4 | Q9NQC3 | YES | [14] |
| SMS1 | Q86VZ5 |  | [8] |
| SMS2 | Q8NHU3 |  | [8] |
| SMVT | Q9Y289 |  | [23] |
| Syntaxin 6 | O43752 |  | [24] |
| SERCA2/ATPA2 | P16615 | YES | [9] |
| Src | P12931 |  | [15] |
| VAMP3 | Q15836 | YES | [25] |
| VAMP4 | O75379 |  | [25] |
| VAMP7 | P51809 |  | [25] |
| VAMP8 | Q9BV40 | YES | [25] |
| VAPA | Q9P0L0 | YES | [8] |
| VAPB | O95292 | YES | [26] |
| Vimentin | P08670 |  | [4] |
| ZNF23 | P17027 |  | [3] |

**References to S2 Table:**

1. Scidmore MA, Hackstadt T (2001) Mammalian 14-3-3beta associates with the Chlamydia trachomatis inclusion membrane via its interaction with IncG. Mol Microbiol 39: 1638-1650.

2. Cox JV, Naher N, Abdelrahman YM, Belland RJ (2012) Host HDL biogenesis machinery is recruited to the inclusion of Chlamydia trachomatis-infected cells and regulates chlamydial growth. Cell Microbiol 14: 1497-1512.

3. Soupene E, Rothschild J, Kuypers FA, Dean D (2012) Eukaryotic protein recruitment into the Chlamydia inclusion: implications for survival and growth. PLoS One 7: e36843.

4. Kumar Y, Valdivia RH (2008) Actin and intermediate filaments stabilize the Chlamydia trachomatis vacuole by forming dynamic structural scaffolds. Cell Host Microbe 4: 159-169.

5. Moorhead AM, Jung JY, Smirnov A, Kaufer S, Scidmore MA (2010) Multiple host proteins that function in phosphatidylinositol-4-phosphate metabolism are recruited to the chlamydial inclusion. Infect Immun 78: 1990-2007.

6. Verbeke P, Welter-Stahl L, Ying S, Hansen J, Hacker G, et al. (2006) Recruitment of BAD by the Chlamydia trachomatis vacuole correlates with host-cell survival. PLoS Pathog 2: e45.

7. Moorhead AR, Rzomp KA, Scidmore MA (2007) The Rab6 effector Bicaudal D1 associates with Chlamydia trachomatis inclusions in a biovar-specific manner. Infect Immun 75: 781-791.

8. Elwell CA, Jiang S, Kim JH, Lee A, Wittmann T, et al. (2011) Chlamydia trachomatis co-opts GBF1 and CERT to acquire host sphingomyelin for distinct roles during intracellular development. PLoS Pathog 7: e1002198.

9. Majeed M, Krause KH, Clark RA, Kihlstrom E, Stendahl O (1999) Localization of intracellular Ca2+ stores in HeLa cells during infection with Chlamydia trachomatis. J Cell Sci 112 ( Pt 1): 35-44.

10. Hasegawa A, Sogo LF, Tan M, Sutterlin C (2009) Host complement regulatory protein CD59 is transported to the chlamydial inclusion by a Golgi apparatus-independent pathway. Infect Immun 77: 1285-1292.

11. Beatty WL (2008) Late endocytic multivesicular bodies intersect the chlamydial inclusion in the absence of CD63. Infect Immun 76: 2872-2881.

12. Beatty WL (2006) Trafficking from CD63-positive late endocytic multivesicular bodies is essential for intracellular development of Chlamydia trachomatis. J Cell Sci 119: 350-359.

13. Pokrovskaya ID, Szwedo JW, Goodwin A, Lupashina TV, Nagarajan UM, et al. (2012) Chlamydia trachomatis hijacks intra-Golgi COG complex-dependent vesicle trafficking pathway. Cell Microbiol 14: 656-668.

14. Dumoux M, Clare DK, Saibil HR, Hayward RD (2012) Chlamydiae assemble a pathogen synapse to hijack the host endoplasmic reticulum. Traffic 13: 1612-1627.

15. Mital J, Miller NJ, Fischer ER, Hackstadt T (2010) Specific chlamydial inclusion membrane proteins associate with active Src family kinases in microdomains that interact with the host microtubule network. Cell Microbiol 12: 1235-1249.

16. Al-Younes HM, Al-Zeer MA, Khalil H, Gussmann J, Karlas A, et al. (2011) Autophagy-independent function of MAP-LC3 during intracellular propagation of Chlamydia trachomatis. Autophagy 7: 814-828.

17. Lutter EI, Barger AC, Nair V, Hackstadt T (2013) Chlamydia trachomatis inclusion membrane protein CT228 recruits elements of the myosin phosphatase pathway to regulate release mechanisms. Cell Rep 3: 1921-1931.

18. Tse SM, Mason D, Botelho RJ, Chiu B, Reyland M, et al. (2005) Accumulation of diacylglycerol in the Chlamydia inclusion vacuole: possible role in the inhibition of host cell apoptosis. J Biol Chem 280: 25210-25215.

19. Rzomp KA, Scholtes LD, Briggs BJ, Whittaker GR, Scidmore MA (2003) Rab GTPases are recruited to chlamydial inclusions in both a species-dependent and species-independent manner. Infect Immun 71: 5855-5870.

20. Leiva N, Capmany A, Damiani MT (2013) Rab11-family of interacting protein 2 associates with chlamydial inclusions through its Rab-binding domain and promotes bacterial multiplication. Cell Microbiol 15: 114-129.

21. Capmany A, Leiva N, Damiani MT (2011) Golgi-associated Rab14, a new regulator for Chlamydia trachomatis infection outcome. Commun Integr Biol 4: 590-593.

22. Gurumurthy RK, Maurer AP, Machuy N, Hess S, Pleissner KP, et al. (2010) A loss-of-function screen reveals Ras- and Raf-independent MEK-ERK signaling during Chlamydia trachomatis infection. Sci Signal 3: ra21.

23. Fisher DJ, Fernandez RE, Maurelli AT (2013) Chlamydia trachomatis transports NAD via the Npt1 ATP/ADP translocase. J Bacteriol 195: 3381-3386.

24. Moore ER, Mead DJ, Dooley CA, Sager J, Hackstadt T (2011) The trans-Golgi SNARE syntaxin 6 is recruited to the chlamydial inclusion membrane. Microbiology 157: 830-838.

25. Delevoye C, Nilges M, Dehoux P, Paumet F, Perrinet S, et al. (2008) SNARE protein mimicry by an intracellular bacterium. PLoS Pathog 4: e1000022.

26. Agaisse H, Derre I (2014) The expression of the effector protein IncD in C. trachomatis mediates the recruitment of the lipid transfer protein CERT and the ER-resident protein VAPB to the inclusion membrane. Infect Immun.
